# Machine Learning–Based Suicide Risk Prediction Model for Suicidal Trajectory on Social Media Following Suicidal Mentions: Independent Algorithm Validation

**DOI:** 10.2196/49927

**Published:** 2024-12-05

**Authors:** Zachary Kaminsky, Robyn J McQuaid, Kim GC Hellemans, Zachary R Patterson, Mysa Saad, Robert L Gabrys, Tetyana Kendzerska, Alfonso Abizaid, Rebecca Robillard

**Affiliations:** 1 University of Ottawa Institute of Mental Health Research at The Royal Ottawa, ON Canada; 2 Department of Cellular and Molecular Medicine University of Ottawa Ottawa, ON Canada; 3 Department of Psychiatry and Behavioral Sciences Johns Hopkins University School of Medicine Baltimore, MD United States; 4 Department of Mental Health Johns Hopkins Bloomberg School of Public Health Baltimore, MD United States; 5 Department of Neuroscience Carleton University Ottawa, ON Canada; 6 Faculty of Medicine University of Ottawa Ottawa, ON Canada; 7 The Ottawa Hospital Research Institute University of Ottawa Ottawa, ON Canada; 8 Department of Psychology University of Ottawa Ottawa, ON Canada

**Keywords:** suicide, prediction, social media, machine learning, suicide risk model, validation, prediction, natural language processing, suicide risk, Twitter, suicidal ideation, suicidal mention

## Abstract

**Background:**

Previous efforts to apply machine learning–based natural language processing to longitudinally collected social media data have shown promise in predicting suicide risk.

**Objective:**

Our primary objective was to externally validate our previous machine learning algorithm, the Suicide Artificial Intelligence Prediction Heuristic (SAIPH), against external survey data in 2 independent cohorts. A second objective was to evaluate the efficacy of SAIPH as an indicator of changing suicidal ideation (SI) over time. The tertiary objective was to use SAIPH to evaluate factors important for improving or worsening suicidal trajectory on social media following suicidal mention.

**Methods:**

Twitter (subsequently rebranded as X) timeline data from a student survey cohort and COVID-19 survey cohort were scored using SAIPH and compared to SI questions on the Beck Depression Inventory and the Self-Report version of the Quick Inventory of Depressive Symptomatology in 159 and 307 individuals, respectively. SAIPH was used to evaluate changing SI trajectory following suicidal mentions in 2 cohorts collected using the Twitter application programming interface.

**Results:**

An interaction of the mean SAIPH score derived from 12 days of Twitter data before survey completion and the average number of posts per day was associated with quantitative SI metrics in each cohort (student survey cohort interaction β=.038, SD 0.014; *F*_4,94_=3.3, *P*=.01; and COVID-19 survey cohort interaction β=.0035, SD 0.0016; *F*_4,493_=2.9, *P*=.03). The slope of average daily SAIPH scores was associated with the change in SI scores within longitudinally followed individuals when evaluating periods of 2 weeks or less (ρ=0.27, *P*=.04). Using SAIPH as an indicator of changing SI, we evaluated SI trajectory in 2 cohorts with suicidal mentions, which identified that those with responses within 72 hours exhibit a significant negative association of the SAIPH score with time in the 3 weeks following suicidal mention (ρ=–0.52, *P*=.02).

**Conclusions:**

Taken together, our results not only validate the association of SAIPH with perceived stress, SI, and changing SI over time but also generate novel methods to evaluate the effects of social media interactions on changing suicidal trajectory.

## Introduction

Suicide is a major public health problem that affects individuals and their families. Previous work has demonstrated that youth may disclose suicidal thoughts on social media [1], and in turn, those expressions of suicidality on social media are associated with suicide attempts [2] and death by suicide [3], suggesting the application of machine learning and natural language processing (NLP) tools to social media text may represent promising areas to generate methods for suicide risk prediction [4]. Several methods focusing on the identification of potentially suicidal tweets have been demonstrated in the past, as outlined by Roy et al [5]. Since that publication, Ophir et al [6] demonstrated that a multilayered machine learning model using deep neural nets incorporating known suicide risk factors and Facebook posts detects suicide risk with the area under the receiver operator characteristic curve (AUC) ranging between 0.70 and 0.75. Notably, the authors observed higher performance of their multilayered machine learning model that incorporated suicide theory than their single-task model that predicts risk from social media posts directly. More recently, novel methods have been developed to attempt to optimize risk detection performance. Sawhney et al [7] used adversarial methods for social media text–based suicide risk classification and demonstrated improvements over other deep learning methods such as convolutional neural networks. Naseem et al [8] applied graph-based hierarchical attention networks to a public Reddit dataset to improve performance of suicide risk prediction, and Li et al [9] used Label-Text Correlation and Deviation Punishment methods to penalize false predictions and generate improved performance. Xu et al [10] demonstrated the power of integrating transfer learning in a targeted fusion approach, potentially applicable to any data source including social media, to generate and demonstrate the efficacy of a predictor of youth’s suicide risk in a hospital setting using a clinical database. While recent efforts have demonstrated the efficacy of using hospital electronic health record data in temporally informed, random forest models [11], importantly, few of the methods leveraging social media data prognosticate future risk of suicidal ideation (SI) and behaviors. The ability of these technologies to predict risk in the future, before periods of acute distress, may allow for the rapid delivery of noninvasive psychosocial interventions and may help to improve access by moving care upstream from primary care facilities. Burkhardt et al [12] applied bidirection transfer learning, integrating clinical data with social media data, to generate improved performance on models indicating suicidal messages and subsequently demonstrated the potential of an automated triage approach to improve response time by 15 minutes. In a recent pilot study, the implementation of online screening of at-risk individuals and delivery of information and counseling-based interventions was well tolerated by responsive individuals and yielded an increase in help-seeking behavior in those who previously reported being unmotivated to seek help [13]. This highlights the promise that online screening technologies may have for suicide screening and intervention; however, as with most of the studies above, this study relies on mentions of suicide-related keywords or in-the-moment expressions of suicide-related thought and, as such, will be limited to those already expressing suicidal sentiments online.

Previous work from our group generated an algorithm entitled the Suicide Artificial Intelligence Prediction Heuristic (SAIPH) [5], which is an NLP algorithm designed to predict future risk of suicidal thoughts and behaviors from social media text data. A key feature of SAIPH is that it efficaciously predicts the risk of SI using data collected before the expression of SI. Briefly, SAIPH converts text-based communications such as tweets into scores for psychological constructs that can be associated with suicide, including depression, anxiety, stress, loneliness, perceived burdensomeness, and hopelessness, among others, and subsequently generates a risk score through the integration of these metrics by a random forest model trained on those with SI mentions (those expressing their SI on Twitter [subsequently rebranded as X]) against controls. Our previous work demonstrated that SAIPH accurately predicted an independent cohort of Twitter users with SI mentions from those without such mentions with an AUC of 0.87 using data before SI mentions, and generated an AUC of 0.70 for identifying those with a history of suicide attempts or plans from those with SI. While promising, these early results were based solely on publicly available SI mentions on Twitter and require external validation in independent datasets of consenting individuals who have answered standardized questions about levels of SI. The primary objective of this study was to validate the efficacy of SAIPH for the prediction of suicidal phenotypes assessed through psychometrically validated scales and to assess the temporal variation of SAIPH scores.

A second important question is not only if our algorithm can prognosticate risk but also if it can act as an indicator of changing SI. Therefore, our secondary objective was to assess the temporal variation of SAIPH scores with changing suicidality in a longitudinal cohort.

Having a proxy indicator of changing SI based on social media will allow for a longitudinal assessment of factors influencing changing SI trajectory, such as the pandemic or being responded to after a suicidal mention. As a third objective, we sought to use SAIPH to evaluate factors important for improving or worsening suicidal trajectory on social media following suicidal mention.

## Methods

### Study Design

This study consisted of 4 cohorts, including 2 recruited through independent online survey efforts and 2 random samplings of individual Twitter users collated with the Twitter application programming interface (API) through *tweepy* (version 3.8.0). Online survey cohort data were derived from the Cannabis Use, Social Media, and Mental Health During the COVID-19 Pandemic Study—a study of undergraduate students at Carleton University in Ottawa (student survey cohort)—and a COVID-19 survey (COVID-19 survey cohort) [14,15]. Both datasets contained basic age and sex demographics, as well as various psychological scales. From these, mood and SI scores were derived as outlined below. Only individuals with active Twitter accounts (those accounts with posts present during this study’s period) who consented to the analysis of tweet data were included in this report. No overlap was present in participants across cohorts.

### Student Survey Cohort

Carleton University students were recruited through the university’s online research system. Participants completed this study during the COVID-19 pandemic, between January and December 2020. Throughout most of this study recruitment (March to December), classes were all being offered online, campuses were closed, and several social restrictions were in place. Participants completed an online survey hosted on Qualtrics. Once the questionnaires were completed, all participants received an online debriefing form and were compensated with course credit.

In total, 159 individuals consented to the use of their Twitter data, enabling the generation of SAIPH scores (mean age 19.32, SD 1.85 y; female to male ratio=67.1%). SI metrics were derived from question 9 on the Beck Depression Inventory (BDI), which was temporally nested in the past 7 days:

- 0=I don’t have any thoughts of harming myself.
- 1=I have thoughts of harming myself, but I would not carry them out.
- 2=(A) I feel I would be better off dead, (B) I have definite plans about committing suicide, (C) I feel my family would be better off if I were dead.
- 3=I would kill myself if I could.

### COVID-19 Survey Cohort

A longitudinal online survey assessing the financial and psychological correlates of the COVID-19 pandemic was distributed via websites, social media, and multiple organizations and hospitals across Canada (please see the full list in the *Acknowledgments* section) [14,15]. The sole inclusion criterion was to be aged 12 years or older. Data included in this report were collected between April 2020 and December 2020. All individuals included in the final sample were from Canada. The survey included the Perceived Stress Scale, the Generalized Anxiety Disorder-7 scale, and the Self-Report version of the Quick Inventory of Depressive Symptomatology (QIDS-SR16).

From the parent study containing 4298 consenting individuals, a total of 307 had Twitter data and consented to their use to enable the generation of SAIPH scores (mean age 49.04, SD 16.53 y; female to male ratio=64.2%). SI was derived using question 12 on the QIDS-SR16, which was also temporally nested in the past 7 days:

- 0=I do not think of suicide or death.
- 1=I feel that life is empty or wonder if it’s worth living.
- 2=I think of suicide or death several times a week for several minutes.
- 3=I think of suicide or death several times a day in some detail, or I have made specific plans for suicide or have actually tried to take my life.

For SI prediction, we modeled SAIPH score data over a range of time frames before the survey completion days to predict SI scores greater than or equal to 1, 2, and 3 in both cohorts and to discriminate between individuals above and below these thresholds. As a subset of 193 individuals consented to longitudinal follow-up and completed surveys at multiple time points, we modeled SI prediction using data from each survey completion period in addition to the baseline survey completion in a manner commensurate with the suicidal mention “event” modeling in our original publication [5].

For proxy SAIPH score analysis (below), 240 individuals from the total cohort with 3 or more longitudinal survey entries and no Twitter accounts were selected.

### Algorithmic Methods

#### Overview

Algorithmic methods for SAIPH, including hyperparameter optimization and performance evaluation, are detailed in our original publication by Roy et al [5], where a higher SAIPH score is indicative of a higher risk of SI. Novel algorithms used in this paper involve the generation of personalized, future SAIPH score–predicting models and the generation of regional proxies for individuals not on Twitter.

#### Personalized, Future SAIPH Score–Predicting Model

For a given individual, an ensemble of linear kernel support vector regression (SVR) models is generated using the *LinearSVR* function in *scikit-learn* (French national Institute for Research in Computer Science and Automation, version 0.21.3) using a stacked generalization approach, trained on mean daily SAIPH data per individual with the outcome being the next day’s mean daily SAIPH value. Training data windows are created separately per individual based on the following. Twitter timeline data are downloaded and processed with SAIPH, after which the number of days for all available past SAIPH data is calculated and divided by 2 to generate a maximum range of possible windows. SVRs are then trained with a minimum window of 14 days and every 7 days prior until the maximum window value is reached or exceeded. Future SAIPH scores are generated through the averaging of all model predictions derived from the most recent data within the model input data range. Linear SVRs were selected over other machine learning methods due to their training speed and success in generating scores correlated with actual future SAIPH scores at the algorithm-building stage (data not shown). A validation strategy comparing predicted values to true metrics was selected over internal k-fold cross-validation strategies and hyperparameter optimization due to the algorithmic generation of multiple machine learning models per individual.

#### Personalized Models to Score Responses to Suicidal Mention Per Individual

We sought to gather and score responses to suicidal mentions on Twitter. Leveraging our existing corpus of suicidal mentions collected between 2016 and 2019 published previously [5], we designed a web interface program using *Selenium* in Python (Selenium Project, GitHub) to pull up the original Tweet ID for the suicidal mention from individuals in the independent testing (not training) set and scrape any direct responses occurring in the thread of that tweet. This method was necessary as the Twitter API allows only for the gathering of response data within the API usage window of 7 days. We limited responses to within 72 hours of the original tweet. From the corpus of 291 suicidal mentions and after eliminating any mentions that were retweets and those for which an account no longer existed, we generated a set of 125 individuals. Each suicidal mention was treated as a separate event, resulting in a total of 471 suicidal mentions, after which the mean SAIPH score per day for 21 days was calculated to act as an indicator of changing SI trajectory over time. We also collected a replication dataset in real time using the Twitter API over several periods during 2019, 2020, 2021, and 2022. Individuals with SI were found by querying for the term “I am suicide thinking, planning.” Tweets were read for those believed to be credible SI by 2 independent raters, after which a custom Python (Python Software Foundation) script was designed to pull responses to that tweet using the Twitter API. Retweets were excluded, while in both cohorts, responses to the original tweet from the original individual who mentioned SI were excluded.

Next, we generated a method to score responses with a custom NLP algorithm designed to assess the personalized relevance of the response for the individual. Briefly, for each individual with a suicidal mention, this personalized model is generated by using SAIPH to score available historical tweets before the SI mention, from which the top and bottom first percentile of tweets were binarily coded using bag-of-words and used to train a personalized SVR model. We can then apply that person’s specific model to the responses they received after their suicidal mention, where messages with low and high scores represent user model–specific positive (favorable) and negative (unfavorable) messages, respectively.

#### Ethical Considerations

For SI mention and response cohorts on Twitter and other randomly selected individuals from Twitter, no participant consent was obtained. According to Canada’s *Tri-Council Policy Statement: Ethical Conduct for Research Involving Humans—*Tri-Council Policy Statement 2 Article 2.5, research on data “in the public domain and the individuals to whom the information refers have no reasonable expectation of privacy (nonintrusive, does not involve direct interaction between the researcher and individuals through the internet)” is exempt from institutional review board or research ethics board (REB) review. This policy is consistent with US regulations where according to US Department of Health and Human Services Policy 45 CFR 46.104 under the heading “exempt research, Secondary research for which consent is not required,” secondary research uses of identifiable private information or identifiable biospecimens are exempt if the identifiable private information or identifiable biospecimens are publicly available. Exemption for the need for REB review was confirmed through the Office of Research Ethics at the Royal Institute of Mental Health Research.

All participants in the COVID-19 survey cohort and student survey cohort gave informed consent. Study protocols for the COVID-19 survey cohort were approved by the Clinical Trials Ontario—Qualified Research Ethics Board via the Ottawa Health Science Network (protocol #2131), and those for the student survey cohort were approved under REB #111775.

#### Statistical Analysis

All statistical tests were performed in R (R Foundation for Statistical Computing). Using an Anderson-Darling test from the *nortest* package, all distributions of data that rejected the null hypothesis of normality were subsequently evaluated with nonparametric tests or robust regression. All statistical tests performed were 2-tailed, and *P*<.05 was considered significant. No correction for multiple comparisons was performed. AUC metrics were generated using the *pROC* package in R. Significance of the AUC was performed using a Monte Carlo card sorting task, randomly permuting case and control status 10,000 times to generate a null distribution of the data for each AUC comparison reported. Where data are referred to as being corrected, this involved subtracting linearly modeled residuals from the dependent variable.

## Results

### Independent Association of SAIPH Scores With Quantitative SI Metrics

**Figure 1:**
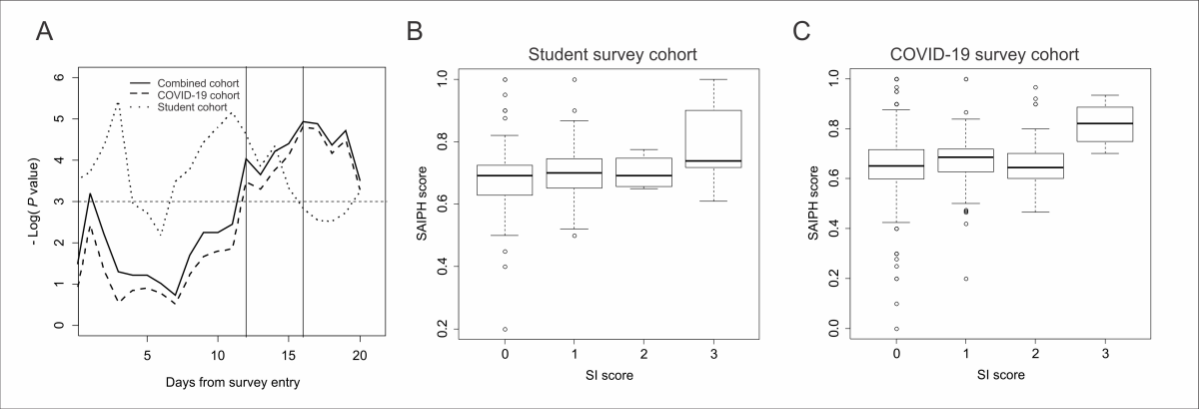
SI associations with SAIPH scores. (A) A plot of the negative natural log of the *P* value (y-axis) of an interaction of the mean daily SAIPH score and number of posts per day on quantitative SI scores as a function of the time in days from the day of survey completion (x-axis). Vertical lines set at 12 and 16 days before the survey completion day demonstrate the earliest period of overlapping significant associations between the student and COVID-19 survey cohorts. Box plots depicting the average 14-day SAIPH score from individuals scoring 0, 1, 2, or 3 on the SI question from the (B) student survey cohort (BDI question 9) and (C) the COVID-19 survey cohort (QIDS-SR16 question 12). BDI: Beck Depression Inventory; QIDS-SR16: Self-Report version of the Quick Inventory of Depressive Symptomatology; SAIPH: Suicide Artificial Intelligence Prediction Heuristic; SI: suicidal ideation.

We investigated SAIPH scores for association with continuous SI metrics from 2 independent mental health metric surveys, including one of Carleton University students in Ottawa, Ontario (student survey cohort; n=153), and one in a national sample of respondents (COVID-19 survey cohort; n=307). Using a sliding window approach and progressively integrating data from successive days before the survey completion day, we observed significant interactions of the mean SAIPH score during these periods and the number of social media posts on the quantitative SI score across both the student survey cohort and the COVID-19 survey cohort (Table S1 and Figure S1 in Multimedia Appendix 1). One day before survey completion, a significant association is observed in the student survey cohort with the quantitative BDI-9 SI score (interaction β=.026, SD 0.011; *F*_4,54_=1.93, *P*=.02), while a nonsignificant trend in the same direction is observed on this day for the COVID-19 survey cohort with the quantitative QIDS-SR score (interaction β=.0017, SD 0.001; *F*_4,353_=3.49, *P*=.09; Table S1 in Multimedia Appendix 1). Consistent overlapping of significant associations next occur at 12 days (student survey cohort interaction β=.038, SD 0.014; *F*_4,94_=3.3, *P*=.01; and COVID-19 survey cohort interaction β=.0035, SD 0.0016; *F*_4,493_=2.9, *P*=.03) and remain significant for up to 16 days (Figure 1A, and Table S1 and Figure S2 in Multimedia Appendix 1). In general, in the student survey cohort, all assessed windows exhibited either significant or nonsignificant trend level associations, while in the COVID-19 survey cohort, similar relationships were observed except for the period of 2 to 11 days after the survey completion day (Figure 1A, and Table S1 and Figure S1A in Multimedia Appendix 1). A post hoc analysis of the combined dataset demonstrated the most robust associations with a minimum of 3 tweets per person per day at 1, 13, and 40 days before the survey completion day (Figure S3 in Multimedia Appendix 1).

Assessment of AUC metrics for binary prediction was also performed on both raw and imputed SAIPH score data and demonstrated similar temporal patterns to those identified in the above interaction models (Sections S1-S3, Tables S2-S4, Figure S4, and Figure S5 in Multimedia Appendix 1; Figures 1B and1C). Cumulatively, the data demonstrate evidence for a moderate association of SAIPH scores with quantitative SI metrics in the initial days before survey completion but that these results become more robust on the integration of more data within the 12- to 16-day period. These results are consistent with our previous work that demonstrated that mean SAIPH score data from a multiple-day window performed better at predicting SI than that from smaller window periods [5]. Notably, SAIPH scores were associated with perceived stress and not depression or anxiety (Section S4 in Multimedia Appendix 1).

### Temporal Assessment of SI Risk

We assessed variations in SAIPH scores over time relative to changes in SI levels in longitudinal follow-up data available in 109 individuals from the COVID-19 survey cohort. Of these, a total of 9 individuals exhibited levels of SI of 1 or greater at a minimum of 1 time point and had tweet information available on the day of survey completion. For this analysis, data points were derived from each successive pair of survey entries. In these participants, we observed a nonsignificant trend in the slope of mean SAIPH scores derived from a survey completion day for each pair of survey completion days relative to the slope of SI levels between 2 data entries (modeled as a function of time between data entry points; n=79 data points; ρ=0.21, *P*=.06).

To increase the available data beyond those who tweeted on the survey response days, we assessed the slope of mean daily SAIPH scores of all days between survey response points relative to the slope of the SI scores as a function of the time in days between survey entries in 109 individuals, of which 35 had variation in SI scores. We observed a significant interaction of the slope of SAIPH scores with the time in days between survey entries on the between survey entry SI slope using a linear regression (β=.00462, SD 0.00085; *F*_4,282_=15.36, *P*<.001). This result remained significant when limiting the sample to 35 individuals demonstrating changes in SI levels (β=.0097, SD 0.0024; *F*_4,93_=15.9, *P*<.001), which remained significant when applying robust regression (β=.013, SD 0.0013; *P*<.001). We performed a sliding window post hoc analysis, limiting the interrogated sample to a range of data points occurring below a range of thresholds from 10 days to 100 days. Significant positive associations of the slope of SI scores and SAIPH score slopes occurred before 15 days (eg, 14 days ρ=0.27, *P*=.04; Figure S6 in Multimedia Appendix 1). Notably, a sliding window analysis evaluating the slope of SAIPH scores between only the 2 survey entry days exhibited nonsignificant trends toward positive associations at longer periods (Figure S6 in Multimedia Appendix 1), suggesting the possibility that variation in SI during longer periods between surveys may be detected by SAIPH and affect the measured slope over longer periods. Similar results were observed when evaluating the slope of perceived stress with the slope of SAIPH scores between periods (Figure S7 in Multimedia Appendix 1). Together, these data suggest that changes in mean SAIPH scores over time reflect temporal changes in SI and can be used as an indicator of changing suicidal distress. For example, the application of SAIPH scores to social media profiles before and after the beginning of the COVID-19 pandemic in 2020 demonstrates significant postpandemic deviations in SAIPH scores between individuals endorsing moderate SI as compared to those who do not (Section S5 and Figure S8 in Multimedia Appendix 1).

### Suicide Indicator Trajectory as a Function of Responses to Suicidal Mentions

**Figure 2:**
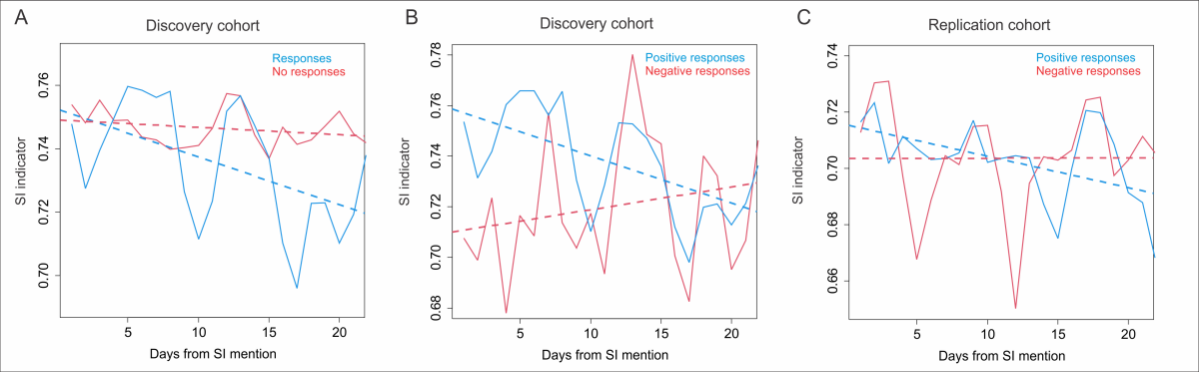
SAIPH trajectory as a function of responses to suicidal mentions. (A) Line graph depicting the mean daily SAIPH score (y-axis) as a function of time in days from suicidal mention (x-axis) in a cohort of individuals with responses to their suicidal mentions (blue) and those with no responses (red). (B) Line graph depicting the mean daily SAIPH score (y-axis) as a function of time in days from suicidal mention (x-axis) in a cohort of individuals with responses scoring as “personally positive” (favorable; blue) and “personally negative” (unfavorable; red) in our originally published corpus of suicidal mentioners from 2016 to 2019. (C) Line graph depicting the mean daily SAIPH score (y-axis) as a function of time in days from suicidal mention (x-axis) in a cohort of individuals with responses scoring as “personally positive” (favorable; blue) and “personally negative” (unfavorable; red) in a replication corpus of those with suicidal mentions collected from 2019 to 2022. SAIPH: Suicide Artificial Intelligence Prediction Heuristic; SI: suicidal ideation.

In light of data suggesting that we could use the SAIPH scoring of Twitter profiles as an indicator of changing suicidal distress over time, we sought to investigate factors that may influence the SAIPH score trajectory after a suicidal mention. To that end, we used 471 suicidal mentions from the corpus of individuals derived from the independent test cohort of our originally published SAIPH paper [5]. Of these, 63 had responses to the suicidal mention within 72 hours, while 408 did not. The mean SAIPH score following each mention was used as an indicator of changing suicidal distress over time. We observed a significant negative association of mean daily SAIPH score in those 63 individuals with responses (ρ=–0.52, *P*=.01), while the group with no responses exhibited no change over time (ρ=–0.27, *P*=.22; Figure 2A). Furthermore, the last 7 days of SAIPH scores in the response group were significantly lower than that of the nonresponse group (Student 2-tailed *t* test: response group, mean 0.72, SD 0.072 and no-response group, mean 0.74, SD 0.085; *P*<.001). These data suggest that individuals who received responses to their suicidal mentions were more likely to improve over 3 weeks (Figure 2A).

As some suicide intervention techniques such as ASIST (Applied Suicide Intervention Skills Training) focus on identifying concepts important to an individual as a key step in convincing them to focus on staying safe, we next reasoned that the quality of responses to suicidal mentions may further influence the trajectory of improvement. To that end, we designed an algorithm to score the importance of responses to suicidal mentions based on the social media profile of users with suicidal mentions. For each individual, all individual tweets were scored with SAIPH, and the 1st (highest scoring) and 99th percentile (lowest scoring) were used to train an SVR per individual. This person-specific model was then used to score those responses to their suicidal mentions and derive a personalized “positivity” score. Using the mean positivity score of 0.243 (SD 0.383) as a threshold, we split the group into 43 individuals with highly positive (favorable) versus 20 highly negative (unfavorable) responses. We observed a significant declining temporal direction in SAIPH scores in the 3 weeks following suicidal mention in those with positive (favorable; ρ=–0.60, *P*=.003) but not negative (unfavorable; ρ=0.22, *P*=.33) responses (Figure 2B). To validate this finding, we collected a replication cohort of those with suicidal mentions from 2019 to 2022 and generated personalized positivity scores for responses to suicidal mentions. Using the same threshold to segregate high and low responders, we observed a similar significant declining temporal direction of association of mean daily SAIPH scores with time over the following 3 weeks in the highly positive response group (N=27, ρ=–0.51, *P*=.02), while no association was observed in the highly negative response group (N=12, ρ=0.0038, *P*=.99; Figure 2C). Together, the data suggest that receiving responses to suicidal mentions on Twitter and specifically those personally scored as positive for an individual is associated with improving SAIPH scores over time following suicidal mentions. We next assessed for overrepresentation of word usage in responses above and below the personalized score threshold in the combined sample of responses from 2016 to 2022. As some individuals received multiple responses for their suicidal mentions, this resulted in a dataset of 99 negatively scored responses and 243 positively scored responses. Among these, no words were enriched in the positively (favorably) scored response corpus (Table S4 in Multimedia Appendix 1); however, in the negatively (unfavorably) scored corpus, several seemingly positive words such as “hope” were significantly overrepresented (Table S5 in Multimedia Appendix 1). Performing the same analysis for pairs of co-occurring words showed that several word pairs exhibited nonsignificant trends for enrichment in the negatively (unfavorably) scored corpus, including “everything will” and “be alright,” while none were significant in the positive (favorable) corpus (Table S4 in Multimedia Appendix 1). We next divided the corpus of responses into those where the user with a suicidal mention had a positive slope of SAIPH scores (becoming worse; n=135 responses) following their suicidal mention, or a negative slope (becoming better; n=191 responses). Again, no enrichment for words in those with improving trajectories was observed (Table S5 and Figure S9 in Multimedia Appendix 1); however, several word pairs were identified in those without improving trajectory such as “please don’t” and “do it,” and the most significant single word was “don’t” (Table S5 in Multimedia Appendix 1).

## Discussion

### Principle Findings

We performed a series of analyses toward the end goal of validating our SI prediction model, SAIPH [5], in 2 cohorts that provided online survey data based on psychometrically validated scales with suicide questions, and in random samples of Twitter users. Both cohorts represented different demographics of the population, with the student survey cohort deriving from an undergraduate university cohort and the COVID-19 survey cohort deriving from individuals with an expansive range of ages throughout the life span. We were limited in our ability to perform a prediction analysis like our original publication due to the small number of high SI scores in both cohorts, as well as the fact that many individuals with high SI scores did not have a large body of tweets in the initial days before survey completion. While our supplementary results identified elevated AUC values across both independent cohorts, these derive from small sample sizes and must be interpreted with caution. As such, we performed linear modeling against the quantitative SI scores to increase the statistical robustness of our analysis and identified significant interactions of SAIPH scores with the number of tweets per day in both cohorts. The interactions demonstrate that SAIPH scores derived from a higher number of tweets on average are more robust. While the associations from the cumulative data from 50 days before the survey completion were significant in both cohorts, we performed a sliding window analysis to identify periods of peak association at periods more temporally proximal to the survey completion day. We observed a general agreement across both cohorts by periods, with more robust associations at approximately 2 weeks before survey completion and, to a lesser degree, on the day before survey completion. The overlapping significance across both cohorts around 2 weeks is consistent with our previous work that showed a higher frequency of high-risk scoring SAIPH scores preceded death by suicide at approximately 20 days before death, while the elevated signal just before the survey completion day is consistent with our previous work that demonstrated the highest predictive accuracy of SI admissions on Twitter using data most temporally proximal to the SI mention. All associations derived from integrating over 42 days of SAIPH scores were significant across both cohorts, which is consistent with the observation that the association with SI is more robust when there is a higher amount of tweet data from which to derive the score. These findings are consistent with our previous work that demonstrated that mean SAIPH score data from a multiple-day window performed better at predicting SI than that from smaller window periods [5]. Cumulatively, both the temporal patterns and observed associations were largely consistent across both cohorts, adding confidence that SAIPH is detecting SI-associated signals.

The SAIPH algorithm is developed on established suicide theories, integrating scores of intermediate suicide-associated constructs such as loneliness, stress, depression, and perceived burdensomeness, among others. Recent work by Ophir et al [6] has demonstrated the success of comparative models using the integration of suicide theory over merely leveraging a bulk of social media posts, suggesting the integration of these constructs may be important for model performance.

It is our view that it is important to consider the application of suicide risk–detecting tools such as SAIPH and others in the context of the challenges of the suicidal state and in what area of suicide prevention these tools may generate the most benefit. One major challenge of suicide risk prediction is the so-called “low base-rate problem” [16], wherein even the best-performing predictors will generate large numbers of false positives. In light of this major problem, it is our view that risk prediction methods could best be applied in contexts where the interventions are tolerable to both false positives and the prevention systems in which they are deployed. For example, it may not be appropriate to deploy in a clinical setting where the response to a false positive will use vital resources in short supply. By contrast, with the presence of future temporal risk predicting tools, one possibility becomes providing a just-in-time adaptive intervention, such as those leveraging digital data sources to prompt the timing of intervention [17], in the community at a time before suicidal crisis that is tailored such that it is tolerable to false positives. One example for which we envision SAIPH might be developed and ultimately applied would be prompting peer support interventions from a list of confidants (such as friends) selected by the at-risk individual, who could be prompted digitally to reach out at an individually set threshold of risk representing a period before the crisis. Such an intervention, if delivered before a period of crisis, would leverage the protective nature of connectedness without the need for mental health intervention training, where the “intervention” might be an invitation to social interaction such as talking, shared activities such as sports, hiking, or going out socially, for example. This may provide protective benefits while also being tolerable to false positives, would be scalable, and could be evaluated for efficacy.

Our temporal analysis in individuals from the COVID-19 survey cohort demonstrated that variation of average daily SAIPH values within people was associated with variation in SI scores over time. This association was strongest when evaluating survey metrics repeated in shorter time frames. We posit that longer periods between surveys may have periods of higher or lower distress that might be marked by variations in SAIPH score that are not captured by survey time points. This supposition is supported by the observation that limiting measurements to those taken on the day of survey completion despite longer periods exhibits a trend toward positive association with the slope of SI changes over the period. Taken together, SAIPH scores may be capable of acting as an indicator of changing SI over time.

We evaluated the ability of SAIPH scores to mark changes in perceived stress, anxiety, and depression. Within participants, the strongest association we observed was with perceived stress. The ability of SAIPH to mark changing perceived stress may be linked with its association as an indicator of changing SI within participants. Notably and consistent with this hypothesis, we observed significant deviations of high SI endorsing individuals with low SI or control individuals as a function of the declaration of a national emergency and the beginning of social distancing due to the start of the COVID-19 pandemic in the middle of March 2020 (Section S5 in Multimedia Appendix 1). Based on the stress diathesis theory, our interpretation is that vulnerable individuals may have experienced higher distress as a function of this shared stressor relative to more resilient controls. This analysis demonstrates the potential efficacy of using SAIPH as an indicator of SI risk and perceived stress to study temporal trends relative to major events, both on an individual and a population basis. This work is consistent with our previous work that demonstrated that averaging of regionally and randomly sampled SAIPH scores from certain areas was associated with suicide death rates in those regions [5].

In light of our data demonstrating that we could use SAIPH as an indicator of changing SI, this allowed us the opportunity to evaluate the effect of responses to suicidal mentions in 2 cohorts. The strongest result we observed was that individuals who had responses to their suicidal mentions demonstrated a significant improvement in their SAIPH scores over the following 3 weeks, suggesting that responding to those in crisis may contribute to their improvement. This result is intuitive and helps corroborate the ability of SAIPH to act as a proxy of SI trajectory. We next attempted to look for a more nuanced scoring method for the responses to qualitatively score them relative to the individual. The impetus for this theory came from the ASIST suicide intervention that teaches that identification of factors important to the individual is a key step in getting the individual with SI to agree to be safe for the moment. As such, our algorithm effectively measures content in their historical posts and builds a new model relative to their most extreme high and low content. If a response to this person mentions something that was positively reflected in their timeline, it will receive a score denoting it is a “positive” response. By building machine learning models specific to the individual, we demonstrated that we could score the response to their suicidal mention and that those with low scores, denoting a response scored as “positive” to the user with a suicidal mention, were more likely to improve. In our analysis of words and 2-word combinations in responses to those with suicidal mentions, no words appeared overrepresented in those individuals who improved over the following 3 weeks as measured by SAIPH. This may be because, as hypothesized, the responses important for someone to improve contained more contextual and personalized content relevant to the individual and, as such, were not overrepresented across the group. By contrast, words such as “don’t” and phrases such as “please don’t,” “do it,” “everything will,” or “be alright” were overrepresented in responses to those with suicidal mentions that did not improve over the 3-week period. This finding suggests that more standard responses may lack the personal context important for an individual’s improvement. These findings, if corroborated, could lead to the development of a tool allowing for quick scanning and identification of topics of importance to a crisis line user who consents to social media scanning. In combination, we envision a possible application of both the SAIPH method and personalized importance metric that could be used by crisis line responders where, through the future integration of large language models and these scores, would allow responders to interrogate the user’s risk and protective factors preceding and following a user’s historical periods of highest risk to gain insight into the person in need. As individuals with SI in crisis can experience cognitive constriction [18-20] and are not always in a mindset for collaborative problem-solving with the crisis responder, these tools could augment suicide intervention efforts, safety planning, and problem-solving and should be further developed. Beyond prompting an ideal response, the data suggest that any response is better than no response. These findings, while interesting, are preliminary and should be further replicated in larger datasets to validate them and further explore their implications.

### Limitations

This study is not without limitations. The SI metrics, while collected as part of psychometrically validated surveys, represented answers to a single question and not, by contrast, the cumulative scores from comprehensive suicide risk scales. Despite this, these metrics are stronger than suicidal mentions on social media as a measure of suicide risk for external validation, as they represent data collections occurring independently from a participant’s social media use and are answers to a standardized set of questions. This fact partially addresses the legitimacy that suicidal mentions on social media are linked to actual suicidal intent as demonstrated in 2 studies from Japan [2,3], and by the fact that our model performs best for identifying BDI SI level 3, which states a desire to kill oneself if possible and thus suggests a level of intent. Despite this, the number of individuals endorsing high levels of SI in both survey cohorts was small and, in some cases, the amount of Twitter data available at the times closest to survey completion was lacking, necessitating the creation of our imputation algorithm. Future work should investigate the efficacy of data imputation using our future predicting SVR method to assess if this improves the robustness of SI prediction in individuals with less data. The primary strategy used involved looking for associations with quantitative SI scores using linear models. The use of the sliding window approach allowed us to investigate the times of most significant association; however, a limitation of this analysis is that we did not correct for multiple testing. Data gathered for this study occurred during the COVID-19 pandemic, which represented a rather anomalous year where in general, people’s usual routines, external influences, and environments were disrupted. In the context of the stress diathesis model of suicide risk, this may have caused a higher efficacy of our prediction methods and observed associations than would not have been observed in more normal times. Our analysis of SAIPH scores before and after this period indicated that the pandemic did seem to affect SAIPH scores in individuals at high suicide risk. Alternatively, it remains possible that the pandemic elevated SAIPH scores in individuals who are not suicidal and led to a weaker observed predictive accuracy than would have otherwise been observed in nonpandemic times. Despite this, the success of SAIPH in predicting SI during pandemic times suggests it is an efficacious tool for suicide risk prevention that is robust in the context of external stressors. Future work should investigate if more personalized approaches, leveraging an individual’s own data to prognosticate risk, may improve predictive performance.

### Conclusions

In conclusion, the above study expands upon the previously published validation efforts of the previous model, SAIPH, on its ability to use Twitter profile data to associate with SI derived from external scales in 2 independent cohorts. Additionally, this study expands on our previous work, demonstrating an association of SAIPH scores with longitudinal changes in perceived stress, which can be used as an indicator of population changes in response to global stressors such as the COVID-19 pandemic. Moreover, our work produced a method to generate future SAIPH score predictions in a personalized manner. Finally, our study demonstrates that analysis of responses to suicidal mentions on social media and the resulting SI trajectory has the potential not only to generate novel algorithms for understanding risk but may inform novel and personalized interventions leveraging an individual’s unique situation. Cumulatively, the work suggests that NLP techniques designed to infer mood from text-based social media content may be efficacious as indicators of individual suicide risk and periods of heightened stress. In the future, methods such as SAIPH may be integrated as tools to aid clinician decisions related to individual suicide risk. The psychiatry field does not have the extensive battery of objective diagnostic tests relative to other fields of medicine and will most likely benefit from the addition of more tools to aid in patient assessment. Longitudinal monitoring of suicide risk indicators on an individual scale will pave the way for the development of novel suicide interventions.

## Appendix

Multimedia Appendix 1 Supplementary results, figures, and tables.

## Abbreviations

API: application programming interface
ASIST: Applied Suicide Intervention Skills Training
AUC: area under the receiver operator characteristic curve
BDI: Beck Depression Inventory
NLP: natural language processing
QIDS-SR16: Self-Report version of the Quick Inventory of Depressive Symptomatology
REB: research ethics board
SAIPH: Suicide Artificial Intelligence Prediction Heuristic
SI: suicidal ideation
SVR: support vector regression

## Data Availability

In accordance with Twitter policies of data sharing, data used in the generation of the algorithm for this study will not be made publicly available. Due to the sensitive nature of study survey cohort data, the research ethics boards have not granted approval for these datasets to be for public dissemination. Due to the sensitive and potentially stigmatizing nature of this tool, code used for algorithm generation or implementation on individual Twitter profiles will not be made publicly available. All Python scripts were created in Python (version 3.6.8), and the machine learning algorithms used *scikit-learn* (version 0.21.3). Specific parameters used to generate, test, and process the current dataset are mentioned in detail in the *Methods* section. Access to Suicide Artificial Intelligence Prediction Heuristic (SAIPH) score generation for research purposes will be granted on reasonable request.
