# Supplementary material for: Machine Learning–Based Suicide Risk Prediction Model for Suicidal Trajectory on Social Media Following Suicidal Mentions: Independent Algorithm Validation
Source: J Med Internet Res. 2024 Dec 5;26:e49927. doi: 10.2196/49927 (PMC11659700; doi:10.2196/49927)
Supplement: Multimedia Appendix 1 [file jmir_v26i1e49927_app1.docx]

**Section S1.** Prediction of SI above various thresholds.

We assessed the ability of mean SAIPH scores derived from a range of periods prior to survey completion to predict SI scores greater than or equal to 1, 2, and 3 in both cohorts and to discriminate between individuals above and below these thresholds. Overall, the algorithm resulted in low predictive discrimination of SI scores below 2 (Table S2).

For both cohorts, the most robust predictive accuracy was obtained when incorporating the average of 2 weeks’ worth of Tweet derived SAIPH data prior to survey entry, achieving AUC values of 0.71 ( SI > 3 N=6, SI < 3 N=94; P=0.042) and 0.87 (SI > 3 N=4, SI < 3 N=506, P=0.004) in the Student and COVID-19 cohorts respectively (Figure S1, Figure S2, Table S1, Section S2). In looking at both cohorts together, the mean SAIPH score from 14 days prior to survey completion discriminated between a total of N=10 SI score entries > 3 from N=600 entries < 3 with an AUC of 0.8 ( P= 3.0x10^-4^) and were significantly associated with quantitative SI metrics (β=0.025 + 0.008, F=10.41, df=1/607, p=0.0013) (Figure 1, Figure S2, Table S1). Detailed tables presenting the predictive performance of SAIPH and imputed SAIPH scores generated to account for missing data are presented across all time points and SI levels in the supplementary material (Section S1, Figure S1, Figure S2, Figure S3,Table S1, Table S2).

**Section S2.** Future imputation of SAIPH scores.

Notably, many individuals had missing data due to not tweeting on the day of the survey (Table S2). We therefore derived a method to impute future SAIPH scores based on historical patterns per individual. We derived an algorithm as outlined in the methods to predict future SAIPH scores based on user specific historical data. We tested this method to predict actual SAIPH score data in N= 244 Twitter users randomly selected from the USA, taking data from the prior 30 days, using the method to generate future scores, integrating these future scores into the next day prediction, and evaluating the limit of the method to predict actual SAIPH scores from the 30 days of true data. A significant over-representation of positive correlations between actual and predicted daily SAIPH data was observed in predictions up to 8 days from the last integration of actual SAIPH data (Figure 3a). We applied the model to longitudinal data available in the COVID-19 cohort in all individuals with more than one longitudinal time point (N=85), in essence, predicting the next day’s SAIPH score, then retraining the models using all available data integrating the true SAIPH score for that day. In this way, we could generate data as if we were predicting future scores from a given day. Where there were gaps, we allowed our models to integrate the predicted score into model training until another day of real user input was available. After adjusting for between subject differences, this approach generates a significant association between the predicted SAIPH score and actual SAIPH scores (β= 0.057 + 0.014, F= 6.044, df= 85/2082, p= 6.01 x 10^-5^). Within individuals, we observed a significant over-representation of positive associations between predicted and actual scores (positive correlations observed =0.67, expected =0.5, p=0.005).

We next reasoned that deviations between the true and predicted scores over a period may indicate increasing or decreasing distress, depending on the direction of the difference. We therefore evaluated the average difference in actual minus predicted scores over the period between changing SI scores and observed a significant negative association after adjusting for between subject differences (β= -1.18 + 0.56, F= 1.05, df=85/140, p= 0.035). This suggests that as the predicted SAIPH score becomes elevated relative to the actual score, this prognosticates an impending increase in suicidality over the period. A similar association was observed when evaluating the change in perceived stress (β= -13.40 + 5.4, F=0.60, df= 76/138, p=0.014) (Figure 3b), controlling for between subject variation.

Using the imputation method, we observed consistent prediction of a greater number of individuals with SI > 3 using the imputed score from the survey day for the Student and COVID-19 cohorts, AUC= 0.72 (95% CI: 0.6-0.86; SI > 3 N=6, SI < 3 N=89) and 0.75 (95% CI: 0.6-9; SI > 3 N=4, SI < 3 N=476), respectively (Table S3, Figure S1). Notably, both the BDI and QIDS-SR-16 instruments ask about suicidal thoughts within the prior week, suggesting that SI outcomes may derive from any day during that period. Prediction accuracies were relatively consistent when assessing the mean imputed SAIPH score derived from the average of multiple days prior to the survey (Table S2), with the notable difference that imputed data performed better at classifying individuals with SI > 2 in the Student but not COVID-19 cohorts. Model performance was less consistent when assessing the mean of multiple days prior to the survey day in SAIPH scores derived from raw Tweet data (Table S2).

The imputation method is designed to allow for training personalized models in a way that is fast and thus scalable, as compared to using alternative ML approaches for temporal data such as long short term memory (LSTM) models, for example, that would take hours to train per person. By contrast, our method takes less than 10 seconds per individual and demonstrated strong association with actual future SAIPH scores. Implementation of the future score imputation method resulted in stronger predictions across both cohorts. It should be noted that data imputation has pros and cons. On the positive side it can help us generate missing data and thus predictions in those for whom it would not have otherwise been possible. On the negative side, imputation can introduce bias by assuming the future results will be similar to patterns observe previously. For example, our promising prediction result may have been driven by the instantiation of future prediction time points from two weeks prior to the survey time point, which was the time of maximal predictive accuracy for both cohorts. Our previous work with SAIPH demonstrated that while higher frequency peaks of high risk scoring SAIPH scores preceded death by suicide, these elevations occurred approximately 20 days prior to death, suggesting a period of lower scoring SAIPH scores or a period of less Tweeting may occur prior to increasing severity of suicidal risk. While important to validate this further, one interpretation is that this may represent a pulling back from social media and external expressions prior to increasing suicidal thought or behavior.

**Section S3.** Model performance in periods under 2 weeks.

Limited performance was observed when limiting data to prior to two weeks of raw Tweet based SAIPH scores, but not imputed SAIPH scores. To assess if this was due to the smaller sample size, we limited the data set to only those individuals with 1 week of data and evaluated the ability of mean 14-day SAIPH scores to predict SI scores of 3 or higher. Should the poor performance be related to smaller sample size and not time, we would expect a similarly poor performance; however, observed AUC values with 14 day data similar to that observed with the entire sample (Student Survey Cohort: N SI > 3 = 5, N SI <3 = 78, AUC= 0.71, 95% CI: 0.43-0.98, COVID-19 Survey cohort: N SI > 3 = 4, N SI <3 = 426, AUC= 0.87, 95% CI: 0.75-0.99). This suggests that time and not subject make up is responsible for lower model performance prior to incorporating approximately 14 days of data in the non-imputed data set.

**Section S4.** Association of SAIPH with perceived stress, anxiety and depression.

In the longitudinal subset of the COVID-19 Survey cohort, we evaluated the association of SAIPH scores on the day of survey response completion date with perceived stress (PSS), anxiety (GAD7), and depression (QIDS-SR16) symptoms. Twitter data and PSS were available for N=47 respondents. We observed a significant association between stress levels and mean SAIPH score using robust regression, adjusting for between- subjects variation as a covariate (β= 12.92 + 3.83, df=74, p=0.0012). No association was observed for depression symptoms (N=49 respondents, β= 4.41 + 13.04, df=58, p=0.73) nor anxiety symptoms (N=46 respondents, β= 2.39+ 13.97, df=73, p=0.86). We next performed a within subjects analysis by averaging SAIPH scores per day in the days between survey response and evaluated the slope of these values with the change in response. As before, anxiety and depression symptoms were not associated (data not shown). Similar to the observations in the within subjects analysis with longitudinal SI score, we observed a significant interaction of the slope of SAIPH scores with the time in days between survey entries on the between survey entry SI slope using robust regression (Interaction β= 0.0045 + 0.002, df=269, p=0.022). As above, a post hoc sliding window analysis demonstrated the strongest associations occurred when assessing slopes between periods less than 14 days; however, a number of positive associations were also observed over longer periods (Figure S3). Together, this data suggests SAIPH scores are indicative of longitudinal changes in perceived stress over time.

**Section S5.** Assessment of SAIPH scores as a function of social distancing mandates.

To demonstrate the utility of leveraging of longitudinal SAIPH metrics over time, we evaluated suicide risk scores with SAIPH for 30 days before and after March 12^th^ 2020, representing the period at which Canada declared the state of emergency in response to the COVID-19 pandemic and shut down businesses. SAIPH scores in the student survey and COVID-19 survey cohorts were significantly higher in the month following the state of emergency (Student Survey Mean Post-State of Emergency= 0.73 + 0.067, Mean Pre-State of Emergency= 0.67 + 0.025, P= 2.07x10^-7^, COVID-19 Survey Mean Post-State of Emergency= 0.67 + 0.016, Mean Pre-State of Emergency= 0.65 + 0.0059, P= 1.76x10^-18^). Post hoc analysis demonstrated that these results were driven by suicidal individuals, specifically those endorsing a score of 2 (“I would like to kill myself”) or higher on the BDI in the student cohort and 2 (“I think of suicide or death several times a week for several minutes.”) or higher on the QIDS-SR16 in the COVID-19 survey cohort (Figure 3) at their baseline survey time point. Specifically suicidal individuals had higher post-emergency SAIPH scores (SI levels at 2 or above: Mean Student Survey Post-State of Emergency= 0.79 + 0.029, Mean Pre-State of Emergency= 0.67 + 0.032, P= 1.3x10^-20^; "Mean COVID-19 Survey Post-State of Emergency= 0.67 + 0.0062, Mean Pre-State of Emergency= 0.66 + 0.098, P= 0.012). Taken together, the data demonstrate a divergence of SAIPH scores changes in response to a major disruption of normal living in individuals endorsing SI.

| **Table S1.** Interaction of SAIPH score and N Tweets as a function of days from survey. | | | | | | | | | | | | | | | | | | | | | | | | | | | | | | | | | | | | | | | | | | | | | | | | | | | | | | | | | | | | | |  |  |  |  |  |  |  |  |  |  |  |  |  |  |
| --- | --- | --- | --- | --- | --- | --- | --- | --- | --- | --- | --- | --- | --- | --- | --- | --- | --- | --- | --- | --- | --- | --- | --- | --- | --- | --- | --- | --- | --- | --- | --- | --- | --- | --- | --- | --- | --- | --- | --- | --- | --- | --- | --- | --- | --- | --- | --- | --- | --- | --- | --- | --- | --- | --- | --- | --- | --- | --- | --- | --- | --- | --- | --- | --- | --- | --- | --- | --- | --- | --- | --- | --- | --- | --- | --- |
| **Student Survey Cohort** | | | | | | | | | | | | | | | | | | | | | | | | | | | | | | | | | | | | | | | | | | | | | | | | | | | | | | | | | | | | | |  |  |  |  |  |  |  |  |  |  |  |  |  |  |
| **Day** | **N Con** | | | | **N SI=1** | | | | | | | | | | **N SI=2** | | | | | | | | **N SI=3** | | | | | **F stat** | | | | | | | **DF** | | | | | | | | **Beta** | | | | | | **SE** | | | | | | **P value** | | | | | | |  |  |  |  |  |  |  |  |  |  |  |  |  |  |
| 0 | 27 | | | | 19 | | | | | | | | | | 4 | | | | | | | | 3 | | | | | 1.73 | | | | | | | 4/49 | | | | | | | | 0.02 | | | | | | 0.0089 | | | | | | 0.0301 | | | | | | |  |  |  |  |  |  |  |  |  |  |  |  |  |  |
| 1 | 29 | | | | 20 | | | | | | | | | | 5 | | | | | | | | 4 | | | | | 1.93 | | | | | | | 4/54 | | | | | | | | 0.026 | | | | | | 0.011 | | | | | | 0.0245 | | | | | | |  |  |  |  |  |  |  |  |  |  |  |  |  |  |
| 2 | 31 | | | | 23 | | | | | | | | | | 5 | | | | | | | | 4 | | | | | 2.19 | | | | | | | 4/59 | | | | | | | | 0.028 | | | | | | 0.011 | | | | | | 0.0131 | | | | | | |  |  |  |  |  |  |  |  |  |  |  |  |  |  |
| 3 | 34 | | | | 23 | | | | | | | | | | 5 | | | | | | | | 4 | | | | | 2.94 | | | | | | | 4/62 | | | | | | | | 0.034 | | | | | | 0.011 | | | | | | 0.0044 | | | | | | |  |  |  |  |  |  |  |  |  |  |  |  |  |  |
| 4 | 36 | | | | 25 | | | | | | | | | | 6 | | | | | | | | 5 | | | | | 1.54 | | | | | | | 4/68 | | | | | | | | 0.027 | | | | | | 0.013 | | | | | | 0.0524 | | | | | | |  |  |  |  |  |  |  |  |  |  |  |  |  |  |
| 5 | 41 | | | | 28 | | | | | | | | | | 6 | | | | | | | | 5 | | | | | 1.31 | | | | | | | 4/76 | | | | | | | | 0.025 | | | | | | 0.013 | | | | | | 0.0647 | | | | | | |  |  |  |  |  |  |  |  |  |  |  |  |  |  |
| 6 | 43 | | | | 29 | | | | | | | | | | 6 | | | | | | | | 5 | | | | | 1.06 | | | | | | | 4/79 | | | | | | | | 0.023 | | | | | | 0.014 | | | | | | 0.112 | | | | | | |  |  |  |  |  |  |  |  |  |  |  |  |  |  |
| 7 | 44 | | | | 33 | | | | | | | | | | 8 | | | | | | | | 5 | | | | | 2.35 | | | | | | | 4/86 | | | | | | | | 0.03 | | | | | | 0.014 | | | | | | 0.0309 | | | | | | |  |  |  |  |  |  |  |  |  |  |  |  |  |  |
| 8 | 43 | | | | 34 | | | | | | | | | | 9 | | | | | | | | 5 | | | | | 1.99 | | | | | | | 4/87 | | | | | | | | 0.033 | | | | | | 0.014 | | | | | | 0.0228 | | | | | | |  |  |  |  |  |  |  |  |  |  |  |  |  |  |
| 9 | 44 | | | | 35 | | | | | | | | | | 9 | | | | | | | | 5 | | | | | 2.74 | | | | | | | 4/89 | | | | | | | | 0.036 | | | | | | 0.014 | | | | | | 0.012 | | | | | | |  |  |  |  |  |  |  |  |  |  |  |  |  |  |
| 10 | 46 | | | | 35 | | | | | | | | | | 9 | | | | | | | | 5 | | | | | 3.63 | | | | | | | 4/91 | | | | | | | | 0.041 | | | | | | 0.015 | | | | | | 0.00823 | | | | | | |  |  |  |  |  |  |  |  |  |  |  |  |  |  |
| 11 | 48 | | | | 35 | | | | | | | | | | 9 | | | | | | | | 5 | | | | | 3.47 | | | | | | | 4/93 | | | | | | | | 0.045 | | | | | | 0.016 | | | | | | 0.00572 | | | | | | |  |  |  |  |  |  |  |  |  |  |  |  |  |  |
| 12 | 49 | | | | 35 | | | | | | | | | | 9 | | | | | | | | 5 | | | | | 3.3 | | | | | | | 4/94 | | | | | | | | 0.038 | | | | | | 0.014 | | | | | | 0.00989 | | | | | | |  |  |  |  |  |  |  |  |  |  |  |  |  |  |
| 13 | 47 | | | | 37 | | | | | | | | | | 10 | | | | | | | | 6 | | | | | 3.05 | | | | | | | 4/96 | | | | | | | | 0.036 | | | | | | 0.015 | | | | | | 0.0213 | | | | | | |  |  |  |  |  |  |  |  |  |  |  |  |  |  |
| 14 | 47 | | | | 38 | | | | | | | | | | 11 | | | | | | | | 6 | | | | | 3.06 | | | | | | | 4/98 | | | | | | | | 0.04 | | | | | | 0.016 | | | | | | 0.013 | | | | | | |  |  |  |  |  |  |  |  |  |  |  |  |  |  |
| 15 | 48 | | | | 38 | | | | | | | | | | 11 | | | | | | | | 6 | | | | | 2.17 | | | | | | | 4/99 | | | | | | | | 0.035 | | | | | | 0.016 | | | | | | 0.0356 | | | | | | |  |  |  |  |  |  |  |  |  |  |  |  |  |  |
| 16 | 48 | | | | 38 | | | | | | | | | | 11 | | | | | | | | 6 | | | | | 2.12 | | | | | | | 4/99 | | | | | | | | 0.031 | | | | | | 0.016 | | | | | | 0.0585 | | | | | | |  |  |  |  |  |  |  |  |  |  |  |  |  |  |
| 17 | 49 | | | | 39 | | | | | | | | | | 11 | | | | | | | | 6 | | | | | 1.94 | | | | | | | 4/101 | | | | | | | | 0.029 | | | | | | 0.016 | | | | | | 0.0776 | | | | | | |  |  |  |  |  |  |  |  |  |  |  |  |  |  |
| 18 | 51 | | | | 39 | | | | | | | | | | 11 | | | | | | | | 6 | | | | | 1.91 | | | | | | | 4/103 | | | | | | | | 0.028 | | | | | | 0.016 | | | | | | 0.0812 | | | | | | |  |  |  |  |  |  |  |  |  |  |  |  |  |  |
| 19 | 54 | | | | 39 | | | | | | | | | | 11 | | | | | | | | 6 | | | | | 1.97 | | | | | | | 4/106 | | | | | | | | 0.031 | | | | | | 0.016 | | | | | | 0.0654 | | | | | | |  |  |  |  |  |  |  |  |  |  |  |  |  |  |
| 20 | 55 | | | | 40 | | | | | | | | | | 11 | | | | | | | | 6 | | | | | 1.93 | | | | | | | 4/108 | | | | | | | | 0.036 | | | | | | 0.017 | | | | | | 0.0388 | | | | | | |  |  |  |  |  |  |  |  |  |  |  |  |  |  |
| 21 | 55 | | | | 41 | | | | | | | | | | 11 | | | | | | | | 6 | | | | | 2.16 | | | | | | | 4/109 | | | | | | | | 0.037 | | | | | | 0.017 | | | | | | 0.032 | | | | | | |  |  |  |  |  |  |  |  |  |  |  |  |  |  |
| 22 | 56 | | | | 42 | | | | | | | | | | 11 | | | | | | | | 6 | | | | | 2.21 | | | | | | | 4/111 | | | | | | | | 0.037 | | | | | | 0.017 | | | | | | 0.033 | | | | | | |  |  |  |  |  |  |  |  |  |  |  |  |  |  |
| 23 | 55 | | | | 43 | | | | | | | | | | 12 | | | | | | | | 6 | | | | | 2.13 | | | | | | | 4/112 | | | | | | | | 0.044 | | | | | | 0.019 | | | | | | 0.0193 | | | | | | |  |  |  |  |  |  |  |  |  |  |  |  |  |  |
| 24 | 55 | | | | 43 | | | | | | | | | | 12 | | | | | | | | 6 | | | | | 2.15 | | | | | | | 4/112 | | | | | | | | 0.045 | | | | | | 0.019 | | | | | | 0.0201 | | | | | | |  |  |  |  |  |  |  |  |  |  |  |  |  |  |
| 25 | 57 | | | | 43 | | | | | | | | | | 12 | | | | | | | | 6 | | | | | 2.85 | | | | | | | 4/114 | | | | | | | | 0.047 | | | | | | 0.019 | | | | | | 0.0148 | | | | | | |  |  |  |  |  |  |  |  |  |  |  |  |  |  |
| 26 | 58 | | | | 44 | | | | | | | | | | 13 | | | | | | | | 6 | | | | | 3.13 | | | | | | | 4/117 | | | | | | | | 0.049 | | | | | | 0.02 | | | | | | 0.0137 | | | | | | |  |  |  |  |  |  |  |  |  |  |  |  |  |  |
| 27 | 58 | | | | 44 | | | | | | | | | | 13 | | | | | | | | 6 | | | | | 3.08 | | | | | | | 4/117 | | | | | | | | 0.047 | | | | | | 0.02 | | | | | | 0.0198 | | | | | | |  |  |  |  |  |  |  |  |  |  |  |  |  |  |
| 28 | 58 | | | | 44 | | | | | | | | | | 13 | | | | | | | | 6 | | | | | 2.66 | | | | | | | 4/117 | | | | | | | | 0.044 | | | | | | 0.02 | | | | | | 0.029 | | | | | | |  |  |  |  |  |  |  |  |  |  |  |  |  |  |
| 29 | 59 | | | | 44 | | | | | | | | | | 13 | | | | | | | | 6 | | | | | 1.8 | | | | | | | 4/118 | | | | | | | | 0.039 | | | | | | 0.021 | | | | | | 0.0665 | | | | | | |  |  |  |  |  |  |  |  |  |  |  |  |  |  |
| 30 | 59 | | | | 45 | | | | | | | | | | 13 | | | | | | | | 6 | | | | | 1.98 | | | | | | | 4/119 | | | | | | | | 0.042 | | | | | | 0.021 | | | | | | 0.0518 | | | | | | |  |  |  |  |  |  |  |  |  |  |  |  |  |  |
| 31 | 59 | | | | 45 | | | | | | | | | | 13 | | | | | | | | 6 | | | | | 1.73 | | | | | | | 4/119 | | | | | | | | 0.042 | | | | | | 0.022 | | | | | | 0.0594 | | | | | | |  |  |  |  |  |  |  |  |  |  |  |  |  |  |
| 32 | 59 | | | | 46 | | | | | | | | | | 13 | | | | | | | | 6 | | | | | 1.92 | | | | | | | 4/120 | | | | | | | | 0.042 | | | | | | 0.021 | | | | | | 0.0491 | | | | | | |  |  |  |  |  |  |  |  |  |  |  |  |  |  |
| 33 | 59 | | | | 47 | | | | | | | | | | 13 | | | | | | | | 6 | | | | | 2.12 | | | | | | | 4/121 | | | | | | | | 0.044 | | | | | | 0.021 | | | | | | 0.0402 | | | | | | |  |  |  |  |  |  |  |  |  |  |  |  |  |  |
| 34 | 60 | | | | 47 | | | | | | | | | | 13 | | | | | | | | 6 | | | | | 2.34 | | | | | | | 4/122 | | | | | | | | 0.045 | | | | | | 0.021 | | | | | | 0.0352 | | | | | | |  |  |  |  |  |  |  |  |  |  |  |  |  |  |
| 35 | 61 | | | | 47 | | | | | | | | | | 13 | | | | | | | | 6 | | | | | 2.19 | | | | | | | 4/123 | | | | | | | | 0.042 | | | | | | 0.021 | | | | | | 0.051 | | | | | | |  |  |  |  |  |  |  |  |  |  |  |  |  |  |
| 36 | 61 | | | | 47 | | | | | | | | | | 13 | | | | | | | | 6 | | | | | 2.39 | | | | | | | 4/123 | | | | | | | | 0.043 | | | | | | 0.021 | | | | | | 0.044 | | | | | | |  |  |  |  |  |  |  |  |  |  |  |  |  |  |
| 37 | 62 | | | | 47 | | | | | | | | | | 13 | | | | | | | | 6 | | | | | 2.64 | | | | | | | 4/124 | | | | | | | | 0.045 | | | | | | 0.021 | | | | | | 0.0332 | | | | | | |  |  |  |  |  |  |  |  |  |  |  |  |  |  |
| 38 | 62 | | | | 47 | | | | | | | | | | 13 | | | | | | | | 6 | | | | | 2.19 | | | | | | | 4/124 | | | | | | | | 0.041 | | | | | | 0.022 | | | | | | 0.0614 | | | | | | |  |  |  |  |  |  |  |  |  |  |  |  |  |  |
| 39 | 63 | | | | 47 | | | | | | | | | | 13 | | | | | | | | 6 | | | | | 2.3 | | | | | | | 4/125 | | | | | | | | 0.041 | | | | | | 0.022 | | | | | | 0.0616 | | | | | | |  |  |  |  |  |  |  |  |  |  |  |  |  |  |
| 40 | 63 | | | | 47 | | | | | | | | | | 13 | | | | | | | | 6 | | | | | 2.53 | | | | | | | 4/125 | | | | | | | | 0.044 | | | | | | 0.022 | | | | | | 0.0426 | | | | | | |  |  |  |  |  |  |  |  |  |  |  |  |  |  |
| 41 | 64 | | | | 47 | | | | | | | | | | 13 | | | | | | | | 6 | | | | | 2.5 | | | | | | | 4/126 | | | | | | | | 0.043 | | | | | | 0.022 | | | | | | 0.0546 | | | | | | |  |  |  |  |  |  |  |  |  |  |  |  |  |  |
| 42 | 66 | | | | 47 | | | | | | | | | | 13 | | | | | | | | 6 | | | | | 2.96 | | | | | | | 4/128 | | | | | | | | 0.047 | | | | | | 0.022 | | | | | | 0.0311 | | | | | | |  |  |  |  |  |  |  |  |  |  |  |  |  |  |
| 43 | 68 | | | | 47 | | | | | | | | | | 13 | | | | | | | | 6 | | | | | 3.38 | | | | | | | 4/130 | | | | | | | | 0.049 | | | | | | 0.022 | | | | | | 0.0241 | | | | | | |  |  |  |  |  |  |  |  |  |  |  |  |  |  |
| 44 | 70 | | | | 47 | | | | | | | | | | 13 | | | | | | | | 6 | | | | | 3.11 | | | | | | | 4/132 | | | | | | | | 0.047 | | | | | | 0.021 | | | | | | 0.031 | | | | | | |  |  |  |  |  |  |  |  |  |  |  |  |  |  |
| 45 | 70 | | | | 47 | | | | | | | | | | 13 | | | | | | | | 6 | | | | | 3.12 | | | | | | | 4/132 | | | | | | | | 0.045 | | | | | | 0.021 | | | | | | 0.0364 | | | | | | |  |  |  |  |  |  |  |  |  |  |  |  |  |  |
| 46 | 71 | | | | 47 | | | | | | | | | | 13 | | | | | | | | 6 | | | | | 2.88 | | | | | | | 4/133 | | | | | | | | 0.045 | | | | | | 0.022 | | | | | | 0.0393 | | | | | | |  |  |  |  |  |  |  |  |  |  |  |  |  |  |
| 47 | 73 | | | | 48 | | | | | | | | | | 13 | | | | | | | | 6 | | | | | 2.68 | | | | | | | 4/136 | | | | | | | | 0.046 | | | | | | 0.022 | | | | | | 0.0337 | | | | | | |  |  |  |  |  |  |  |  |  |  |  |  |  |  |
| 48 | 74 | | | | 48 | | | | | | | | | | 13 | | | | | | | | 6 | | | | | 2.66 | | | | | | | 4/137 | | | | | | | | 0.047 | | | | | | 0.021 | | | | | | 0.0279 | | | | | | |  |  |  |  |  |  |  |  |  |  |  |  |  |  |
| 49 | 74 | | | | 48 | | | | | | | | | | 13 | | | | | | | | 6 | | | | | 2.63 | | | | | | | 4/137 | | | | | | | | 0.048 | | | | | | 0.022 | | | | | | 0.0282 | | | | | | |  |  |  |  |  |  |  |  |  |  |  |  |  |  |
| 50 | 74 | | | | 48 | | | | | | | | | | 13 | | | | | | | | 6 | | | | | 2.63 | | | | | | | 4/137 | | | | | | | | 0.048 | | | | | | 0.022 | | | | | | 0.0282 | | | | | | |  |  |  |  |  |  |  |  |  |  |  |  |  |  |
| **COVID-19 Survey Cohort** | | | | | | | | | | | | | | | | | | | | | | | | | | | | | | | | | | | | | | | | | | | | | | | | | | | | | | | | | | | | | |  |  |  |  |  |  |  |  |  |  |  |  |  |  |
| **Day** | **N Con** | | | | **N SI=1** | | | | | | | | | | **N SI=2** | | | | | | | | **N SI=3** | | | | | **F stat** | | | | | | | **DF** | | | | | | | | **Beta** | | | | | | **SE** | | | | | | **P value** | | | | | | |  |  |  |  |  |  |  |  |  |  |  |  |  |  |
| 0 | 232 | | | | 63 | | | | | | | | | | 15 | | | | | | | | 0 | | | | | 0.88 | | | | | | | 4/306 | | | | | | | | 5.00E-04 | | | | | | 0.00076 | | | | | | 0.511 | | | | | | |  |  |  |  |  |  |  |  |  |  |  |  |  |  |
| 1 | 268 | | | | 71 | | | | | | | | | | 17 | | | | | | | | 1 | | | | | 3.49 | | | | | | | 4/353 | | | | | | | | 0.0017 | | | | | | 0.001 | | | | | | 0.0884 | | | | | | |  |  |  |  |  |  |  |  |  |  |  |  |  |  |
| 2 | 277 | | | | 78 | | | | | | | | | | 21 | | | | | | | | 1 | | | | | 1.85 | | | | | | | 4/373 | | | | | | | | 0.0013 | | | | | | 0.0012 | | | | | | 0.261 | | | | | | |  |  |  |  |  |  |  |  |  |  |  |  |  |  |
| 3 | 287 | | | | 86 | | | | | | | | | | 26 | | | | | | | | 3 | | | | | 0.74 | | | | | | | 4/398 | | | | | | | | 0.00071 | | | | | | 0.0013 | | | | | | 0.572 | | | | | | |  |  |  |  |  |  |  |  |  |  |  |  |  |  |
| 4 | 296 | | | | 88 | | | | | | | | | | 28 | | | | | | | | 3 | | | | | 0.81 | | | | | | | 4/411 | | | | | | | | 0.001 | | | | | | 0.0013 | | | | | | 0.43 | | | | | | |  |  |  |  |  |  |  |  |  |  |  |  |  |  |
| 5 | 307 | | | | 90 | | | | | | | | | | 29 | | | | | | | | 3 | | | | | 0.71 | | | | | | | 4/425 | | | | | | | | 0.0011 | | | | | | 0.0013 | | | | | | 0.405 | | | | | | |  |  |  |  |  |  |  |  |  |  |  |  |  |  |
| 6 | 326 | | | | 90 | | | | | | | | | | 29 | | | | | | | | 3 | | | | | 0.23 | | | | | | | 4/444 | | | | | | | | 0.0011 | | | | | | 0.0014 | | | | | | 0.458 | | | | | | |  |  |  |  |  |  |  |  |  |  |  |  |  |  |
| 7 | 335 | | | | 90 | | | | | | | | | | 29 | | | | | | | | 3 | | | | | 0.13 | | | | | | | 4/453 | | | | | | | | 0.00079 | | | | | | 0.0015 | | | | | | 0.591 | | | | | | |  |  |  |  |  |  |  |  |  |  |  |  |  |  |
| 8 | 344 | | | | 93 | | | | | | | | | | 29 | | | | | | | | 3 | | | | | 0.68 | | | | | | | 4/465 | | | | | | | | 0.0016 | | | | | | 0.0015 | | | | | | 0.291 | | | | | | |  |  |  |  |  |  |  |  |  |  |  |  |  |  |
| 9 | 348 | | | | 95 | | | | | | | | | | 30 | | | | | | | | 3 | | | | | 0.76 | | | | | | | 4/472 | | | | | | | | 0.0021 | | | | | | 0.0016 | | | | | | 0.188 | | | | | | |  |  |  |  |  |  |  |  |  |  |  |  |  |  |
| 10 | 354 | | | | 95 | | | | | | | | | | 30 | | | | | | | | 3 | | | | | 0.83 | | | | | | | 4/478 | | | | | | | | 0.0022 | | | | | | 0.0016 | | | | | | 0.166 | | | | | | |  |  |  |  |  |  |  |  |  |  |  |  |  |  |
| 11 | 360 | | | | 98 | | | | | | | | | | 32 | | | | | | | | 3 | | | | | 1.46 | | | | | | | 4/489 | | | | | | | | 0.0024 | | | | | | 0.0017 | | | | | | 0.156 | | | | | | |  |  |  |  |  |  |  |  |  |  |  |  |  |  |
| 12 | 361 | | | | 99 | | | | | | | | | | 33 | | | | | | | | 4 | | | | | 2.9 | | | | | | | 4/493 | | | | | | | | 0.0035 | | | | | | 0.0016 | | | | | | 0.0309 | | | | | | |  |  |  |  |  |  |  |  |  |  |  |  |  |  |
| 13 | 368 | | | | 99 | | | | | | | | | | 33 | | | | | | | | 4 | | | | | 3.18 | | | | | | | 4/500 | | | | | | | | 0.0034 | | | | | | 0.0016 | | | | | | 0.037 | | | | | | |  |  |  |  |  |  |  |  |  |  |  |  |  |  |
| 14 | 375 | | | | 101 | | | | | | | | | | 33 | | | | | | | | 4 | | | | | 2.83 | | | | | | | 4/509 | | | | | | | | 0.0038 | | | | | | 0.0017 | | | | | | 0.0233 | | | | | | |  |  |  |  |  |  |  |  |  |  |  |  |  |  |
| 15 | 381 | | | | 102 | | | | | | | | | | 33 | | | | | | | | 4 | | | | | 2.56 | | | | | | | 4/516 | | | | | | | | 0.0041 | | | | | | 0.0017 | | | | | | 0.0164 | | | | | | |  |  |  |  |  |  |  |  |  |  |  |  |  |  |
| 16 | 384 | | | | 102 | | | | | | | | | | 33 | | | | | | | | 4 | | | | | 2.8 | | | | | | | 4/519 | | | | | | | | 0.0044 | | | | | | 0.0017 | | | | | | 0.00808 | | | | | | |  |  |  |  |  |  |  |  |  |  |  |  |  |  |
| 17 | 386 | | | | 103 | | | | | | | | | | 33 | | | | | | | | 4 | | | | | 2.66 | | | | | | | 4/522 | | | | | | | | 0.0045 | | | | | | 0.0017 | | | | | | 0.00867 | | | | | | |  |  |  |  |  |  |  |  |  |  |  |  |  |  |
| 18 | 391 | | | | 104 | | | | | | | | | | 33 | | | | | | | | 4 | | | | | 2.21 | | | | | | | 4/528 | | | | | | | | 0.004 | | | | | | 0.0016 | | | | | | 0.0155 | | | | | | |  |  |  |  |  |  |  |  |  |  |  |  |  |  |
| 19 | 395 | | | | 105 | | | | | | | | | | 33 | | | | | | | | 4 | | | | | 2.25 | | | | | | | 4/533 | | | | | | | | 0.0041 | | | | | | 0.0016 | | | | | | 0.0114 | | | | | | |  |  |  |  |  |  |  |  |  |  |  |  |  |  |
| 20 | 400 | | | | 105 | | | | | | | | | | 33 | | | | | | | | 4 | | | | | 1.56 | | | | | | | 4/538 | | | | | | | | 0.0031 | | | | | | 0.0015 | | | | | | 0.0378 | | | | | | |  |  |  |  |  |  |  |  |  |  |  |  |  |  |
| 21 | 401 | | | | 107 | | | | | | | | | | 34 | | | | | | | | 4 | | | | | 1.55 | | | | | | | 4/542 | | | | | | | | 0.0029 | | | | | | 0.0015 | | | | | | 0.0575 | | | | | | |  |  |  |  |  |  |  |  |  |  |  |  |  |  |
| 22 | 404 | | | | 108 | | | | | | | | | | 34 | | | | | | | | 4 | | | | | 1.37 | | | | | | | 4/546 | | | | | | | | 0.0027 | | | | | | 0.0015 | | | | | | 0.0823 | | | | | | |  |  |  |  |  |  |  |  |  |  |  |  |  |  |
| 23 | 406 | | | | 110 | | | | | | | | | | 36 | | | | | | | | 5 | | | | | 1.38 | | | | | | | 4/553 | | | | | | | | 0.0028 | | | | | | 0.0016 | | | | | | 0.0788 | | | | | | |  |  |  |  |  |  |  |  |  |  |  |  |  |  |
| 24 | 406 | | | | 111 | | | | | | | | | | 36 | | | | | | | | 5 | | | | | 2.06 | | | | | | | 4/554 | | | | | | | | 0.004 | | | | | | 0.0017 | | | | | | 0.0238 | | | | | | |  |  |  |  |  |  |  |  |  |  |  |  |  |  |
| 25 | 413 | | | | 111 | | | | | | | | | | 36 | | | | | | | | 5 | | | | | 1.8 | | | | | | | 4/561 | | | | | | | | 0.0038 | | | | | | 0.0018 | | | | | | 0.0344 | | | | | | |  |  |  |  |  |  |  |  |  |  |  |  |  |  |
| 26 | 414 | | | | 112 | | | | | | | | | | 37 | | | | | | | | 6 | | | | | 1.76 | | | | | | | 4/565 | | | | | | | | 0.0036 | | | | | | 0.0018 | | | | | | 0.0478 | | | | | | |  |  |  |  |  |  |  |  |  |  |  |  |  |  |
| 27 | 416 | | | | 112 | | | | | | | | | | 37 | | | | | | | | 6 | | | | | 1.95 | | | | | | | 4/567 | | | | | | | | 0.0038 | | | | | | 0.0018 | | | | | | 0.0376 | | | | | | |  |  |  |  |  |  |  |  |  |  |  |  |  |  |
| 28 | 422 | | | | 112 | | | | | | | | | | 37 | | | | | | | | 6 | | | | | 2.05 | | | | | | | 4/573 | | | | | | | | 0.004 | | | | | | 0.0018 | | | | | | 0.0299 | | | | | | |  |  |  |  |  |  |  |  |  |  |  |  |  |  |
| 29 | 424 | | | | 113 | | | | | | | | | | 37 | | | | | | | | 6 | | | | | 2.22 | | | | | | | 4/576 | | | | | | | | 0.0039 | | | | | | 0.0018 | | | | | | 0.0286 | | | | | | |  |  |  |  |  |  |  |  |  |  |  |  |  |  |
| 30 | 424 | | | | 113 | | | | | | | | | | 37 | | | | | | | | 6 | | | | | 2.21 | | | | | | | 4/576 | | | | | | | | 0.0039 | | | | | | 0.0018 | | | | | | 0.029 | | | | | | |  |  |  |  |  |  |  |  |  |  |  |  |  |  |
| 31 | 424 | | | | 113 | | | | | | | | | | 37 | | | | | | | | 6 | | | | | 2.29 | | | | | | | 4/576 | | | | | | | | 0.0041 | | | | | | 0.0018 | | | | | | 0.0261 | | | | | | |  |  |  |  |  |  |  |  |  |  |  |  |  |  |
| 32 | 426 | | | | 113 | | | | | | | | | | 37 | | | | | | | | 6 | | | | | 2.22 | | | | | | | 4/578 | | | | | | | | 0.0041 | | | | | | 0.0018 | | | | | | 0.0273 | | | | | | |  |  |  |  |  |  |  |  |  |  |  |  |  |  |
| 33 | 427 | | | | 113 | | | | | | | | | | 37 | | | | | | | | 6 | | | | | 2.1 | | | | | | | 4/579 | | | | | | | | 0.0039 | | | | | | 0.0019 | | | | | | 0.0332 | | | | | | |  |  |  |  |  |  |  |  |  |  |  |  |  |  |
| 34 | 429 | | | | 113 | | | | | | | | | | 37 | | | | | | | | 6 | | | | | 2.41 | | | | | | | 4/581 | | | | | | | | 0.0043 | | | | | | 0.0019 | | | | | | 0.0212 | | | | | | |  |  |  |  |  |  |  |  |  |  |  |  |  |  |
| 35 | 432 | | | | 113 | | | | | | | | | | 37 | | | | | | | | 6 | | | | | 2.65 | | | | | | | 4/584 | | | | | | | | 0.0044 | | | | | | 0.0018 | | | | | | 0.0177 | | | | | | |  |  |  |  |  |  |  |  |  |  |  |  |  |  |
| 36 | 433 | | | | 113 | | | | | | | | | | 37 | | | | | | | | 6 | | | | | 2.59 | | | | | | | 4/585 | | | | | | | | 0.0044 | | | | | | 0.0019 | | | | | | 0.0181 | | | | | | |  |  |  |  |  |  |  |  |  |  |  |  |  |  |
| 37 | 435 | | | | 113 | | | | | | | | | | 37 | | | | | | | | 6 | | | | | 2.61 | | | | | | | 4/587 | | | | | | | | 0.0044 | | | | | | 0.0018 | | | | | | 0.0165 | | | | | | |  |  |  |  |  |  |  |  |  |  |  |  |  |  |
| 38 | 435 | | | | 113 | | | | | | | | | | 37 | | | | | | | | 6 | | | | | 2.84 | | | | | | | 4/587 | | | | | | | | 0.0045 | | | | | | 0.0019 | | | | | | 0.0152 | | | | | | |  |  |  |  |  |  |  |  |  |  |  |  |  |  |
| 39 | 438 | | | | 113 | | | | | | | | | | 37 | | | | | | | | 6 | | | | | 2.83 | | | | | | | 4/590 | | | | | | | | 0.0045 | | | | | | 0.0019 | | | | | | 0.0167 | | | | | | |  |  |  |  |  |  |  |  |  |  |  |  |  |  |
| 40 | 440 | | | | 114 | | | | | | | | | | 38 | | | | | | | | 6 | | | | | 2.85 | | | | | | | 4/594 | | | | | | | | 0.0046 | | | | | | 0.0019 | | | | | | 0.0159 | | | | | | |  |  |  |  |  |  |  |  |  |  |  |  |  |  |
| 41 | 443 | | | | 115 | | | | | | | | | | 39 | | | | | | | | 6 | | | | | 2.66 | | | | | | | 4/599 | | | | | | | | 0.0048 | | | | | | 0.0019 | | | | | | 0.0139 | | | | | | |  |  |  |  |  |  |  |  |  |  |  |  |  |  |
| 42 | 445 | | | | 115 | | | | | | | | | | 39 | | | | | | | | 6 | | | | | 2.78 | | | | | | | 4/601 | | | | | | | | 0.0049 | | | | | | 0.0019 | | | | | | 0.0116 | | | | | | |  |  |  |  |  |  |  |  |  |  |  |  |  |  |
| 43 | 450 | | | | 115 | | | | | | | | | | 39 | | | | | | | | 6 | | | | | 2.87 | | | | | | | 4/606 | | | | | | | | 0.005 | | | | | | 0.0019 | | | | | | 0.011 | | | | | | |  |  |  |  |  |  |  |  |  |  |  |  |  |  |
| 44 | 451 | | | | 115 | | | | | | | | | | 39 | | | | | | | | 6 | | | | | 2.97 | | | | | | | 4/607 | | | | | | | | 0.0051 | | | | | | 0.002 | | | | | | 0.00954 | | | | | | |  |  |  |  |  |  |  |  |  |  |  |  |  |  |
| 45 | 453 | | | | 115 | | | | | | | | | | 39 | | | | | | | | 6 | | | | | 3.01 | | | | | | | 4/609 | | | | | | | | 0.005 | | | | | | 0.002 | | | | | | 0.0108 | | | | | | |  |  |  |  |  |  |  |  |  |  |  |  |  |  |
| 46 | 457 | | | | 115 | | | | | | | | | | 39 | | | | | | | | 6 | | | | | 2.96 | | | | | | | 4/613 | | | | | | | | 0.005 | | | | | | 0.002 | | | | | | 0.0117 | | | | | | |  |  |  |  |  |  |  |  |  |  |  |  |  |  |
| 47 | 458 | | | | 115 | | | | | | | | | | 39 | | | | | | | | 6 | | | | | 2.94 | | | | | | | 4/614 | | | | | | | | 0.0049 | | | | | | 0.002 | | | | | | 0.013 | | | | | | |  |  |  |  |  |  |  |  |  |  |  |  |  |  |
| 48 | 460 | | | | 115 | | | | | | | | | | 39 | | | | | | | | 6 | | | | | 3 | | | | | | | 4/616 | | | | | | | | 0.0049 | | | | | | 0.002 | | | | | | 0.0127 | | | | | | |  |  |  |  |  |  |  |  |  |  |  |  |  |  |
| 49 | 469 | | | | 116 | | | | | | | | | | 39 | | | | | | | | 6 | | | | | 2.85 | | | | | | | 4/626 | | | | | | | | 0.0049 | | | | | | 0.0019 | | | | | | 0.0111 | | | | | | |  |  |  |  |  |  |  |  |  |  |  |  |  |  |
| 50 | 469 | | | | 116 | | | | | | | | | | 39 | | | | | | | | 6 | | | | | 2.85 | | | | | | | 4/626 | | | | | | | | 0.0049 | | | | | | 0.0019 | | | | | | 0.0111 | | | | | | |  |  |  |  |  |  |  |  |  |  |  |  |  |  |
| **Combined** | | | | | | | | | | | | | | | | | | | | | | | | | | | | | | | | | | | | | | | | | | | | | | | | | | | | | | | | | | | | | |  |  |  |  |  |  |  |  |  |  |  |  |  |  |
| **Day** | **N Con** | | | | **N SI=1** | | | | | | | | | | **N SI=2** | | | | | | | | **N SI=3** | | | | | **F stat** | | | | | | | **DF** | | | | | | | | **Beta** | | | | | | **SE** | | | | | | **P value** | | | | | | |  |  |  |  |  |  |  |  |  |  |  |  |  |  |
| 0 | 259 | | | | 82 | | | | | | | | | | 19 | | | | | | | | 3 | | | | | 0.99 | | | | | | | 4/359 | | | | | | | | 0.00087 | | | | | | 0.00082 | | | | | | 0.29 | | | | | | |  |  |  |  |  |  |  |  |  |  |  |  |  |  |
| 1 | 297 | | | | 91 | | | | | | | | | | 22 | | | | | | | | 5 | | | | | 3.77 | | | | | | | 4/411 | | | | | | | | 0.0023 | | | | | | 0.0011 | | | | | | 0.041 | | | | | | |  |  |  |  |  |  |  |  |  |  |  |  |  |  |
| 2 | 308 | | | | 101 | | | | | | | | | | 26 | | | | | | | | 5 | | | | | 2.03 | | | | | | | 4/436 | | | | | | | | 0.002 | | | | | | 0.0012 | | | | | | 0.109 | | | | | | |  |  |  |  |  |  |  |  |  |  |  |  |  |  |
| 3 | 321 | | | | 109 | | | | | | | | | | 31 | | | | | | | | 7 | | | | | 0.97 | | | | | | | 4/464 | | | | | | | | 0.0015 | | | | | | 0.0013 | | | | | | 0.271 | | | | | | |  |  |  |  |  |  |  |  |  |  |  |  |  |  |
| 4 | 332 | | | | 113 | | | | | | | | | | 34 | | | | | | | | 8 | | | | | 1.27 | | | | | | | 4/483 | | | | | | | | 0.0015 | | | | | | 0.0014 | | | | | | 0.295 | | | | | | |  |  |  |  |  |  |  |  |  |  |  |  |  |  |
| 5 | 348 | | | | 118 | | | | | | | | | | 35 | | | | | | | | 8 | | | | | 1.11 | | | | | | | 4/505 | | | | | | | | 0.0015 | | | | | | 0.0014 | | | | | | 0.297 | | | | | | |  |  |  |  |  |  |  |  |  |  |  |  |  |  |
| 6 | 369 | | | | 119 | | | | | | | | | | 35 | | | | | | | | 8 | | | | | 0.47 | | | | | | | 4/527 | | | | | | | | 0.0014 | | | | | | 0.0015 | | | | | | 0.363 | | | | | | |  |  |  |  |  |  |  |  |  |  |  |  |  |  |
| 7 | 379 | | | | 123 | | | | | | | | | | 37 | | | | | | | | 8 | | | | | 0.64 | | | | | | | 4/543 | | | | | | | | 0.0011 | | | | | | 0.0016 | | | | | | 0.482 | | | | | | |  |  |  |  |  |  |  |  |  |  |  |  |  |  |
| 8 | 387 | | | | 127 | | | | | | | | | | 38 | | | | | | | | 8 | | | | | 1.22 | | | | | | | 4/556 | | | | | | | | 0.0022 | | | | | | 0.0016 | | | | | | 0.182 | | | | | | |  |  |  |  |  |  |  |  |  |  |  |  |  |  |
| 9 | 392 | | | | 130 | | | | | | | | | | 39 | | | | | | | | 8 | | | | | 1.63 | | | | | | | 4/565 | | | | | | | | 0.0027 | | | | | | 0.0017 | | | | | | 0.105 | | | | | | |  |  |  |  |  |  |  |  |  |  |  |  |  |  |
| 10 | 400 | | | | 130 | | | | | | | | | | 39 | | | | | | | | 8 | | | | | 2.02 | | | | | | | 4/573 | | | | | | | | 0.0028 | | | | | | 0.0017 | | | | | | 0.105 | | | | | | |  |  |  |  |  |  |  |  |  |  |  |  |  |  |
| 11 | 408 | | | | 133 | | | | | | | | | | 41 | | | | | | | | 8 | | | | | 2.87 | | | | | | | 4/586 | | | | | | | | 0.003 | | | | | | 0.0018 | | | | | | 0.0869 | | | | | | |  |  |  |  |  |  |  |  |  |  |  |  |  |  |
| 12 | 410 | | | | 134 | | | | | | | | | | 42 | | | | | | | | 9 | | | | | 4.54 | | | | | | | 4/591 | | | | | | | | 0.004 | | | | | | 0.0017 | | | | | | 0.0177 | | | | | | |  |  |  |  |  |  |  |  |  |  |  |  |  |  |
| 13 | 415 | | | | 136 | | | | | | | | | | 43 | | | | | | | | 10 | | | | | 5.18 | | | | | | | 4/600 | | | | | | | | 0.0039 | | | | | | 0.0018 | | | | | | 0.026 | | | | | | |  |  |  |  |  |  |  |  |  |  |  |  |  |  |
| 14 | 422 | | | | 139 | | | | | | | | | | 44 | | | | | | | | 10 | | | | | 4.42 | | | | | | | 4/611 | | | | | | | | 0.0044 | | | | | | 0.0018 | | | | | | 0.0148 | | | | | | |  |  |  |  |  |  |  |  |  |  |  |  |  |  |
| 15 | 429 | | | | 140 | | | | | | | | | | 44 | | | | | | | | 10 | | | | | 3.75 | | | | | | | 4/619 | | | | | | | | 0.0046 | | | | | | 0.0018 | | | | | | 0.0123 | | | | | | |  |  |  |  |  |  |  |  |  |  |  |  |  |  |
| 16 | 432 | | | | 140 | | | | | | | | | | 44 | | | | | | | | 10 | | | | | 3.84 | | | | | | | 4/622 | | | | | | | | 0.0048 | | | | | | 0.0018 | | | | | | 0.00718 | | | | | | |  |  |  |  |  |  |  |  |  |  |  |  |  |  |
| 17 | 435 | | | | 142 | | | | | | | | | | 44 | | | | | | | | 10 | | | | | 3.72 | | | | | | | 4/627 | | | | | | | | 0.0049 | | | | | | 0.0018 | | | | | | 0.00761 | | | | | | |  |  |  |  |  |  |  |  |  |  |  |  |  |  |
| 18 | 442 | | | | 143 | | | | | | | | | | 44 | | | | | | | | 10 | | | | | 3.2 | | | | | | | 4/635 | | | | | | | | 0.0044 | | | | | | 0.0018 | | | | | | 0.0128 | | | | | | |  |  |  |  |  |  |  |  |  |  |  |  |  |  |
| 19 | 449 | | | | 144 | | | | | | | | | | 44 | | | | | | | | 10 | | | | | 3.08 | | | | | | | 4/643 | | | | | | | | 0.0046 | | | | | | 0.0017 | | | | | | 0.00904 | | | | | | |  |  |  |  |  |  |  |  |  |  |  |  |  |  |
| 20 | 455 | | | | 145 | | | | | | | | | | 44 | | | | | | | | 10 | | | | | 2.18 | | | | | | | 4/650 | | | | | | | | 0.0035 | | | | | | 0.0016 | | | | | | 0.0304 | | | | | | |  |  |  |  |  |  |  |  |  |  |  |  |  |  |
| 21 | 456 | | | | 148 | | | | | | | | | | 45 | | | | | | | | 10 | | | | | 2.47 | | | | | | | 4/655 | | | | | | | | 0.0033 | | | | | | 0.0016 | | | | | | 0.0418 | | | | | | |  |  |  |  |  |  |  |  |  |  |  |  |  |  |
| 22 | 460 | | | | 150 | | | | | | | | | | 45 | | | | | | | | 10 | | | | | 2.31 | | | | | | | 4/661 | | | | | | | | 0.0031 | | | | | | 0.0016 | | | | | | 0.0594 | | | | | | |  |  |  |  |  |  |  |  |  |  |  |  |  |  |
| 23 | 461 | | | | 153 | | | | | | | | | | 48 | | | | | | | | 11 | | | | | 2.06 | | | | | | | 4/669 | | | | | | | | 0.0033 | | | | | | 0.0017 | | | | | | 0.0502 | | | | | | |  |  |  |  |  |  |  |  |  |  |  |  |  |  |
| 24 | 461 | | | | 154 | | | | | | | | | | 48 | | | | | | | | 11 | | | | | 2.88 | | | | | | | 4/670 | | | | | | | | 0.0046 | | | | | | 0.0019 | | | | | | 0.0133 | | | | | | |  |  |  |  |  |  |  |  |  |  |  |  |  |  |
| 25 | 470 | | | | 154 | | | | | | | | | | 48 | | | | | | | | 11 | | | | | 2.91 | | | | | | | 4/679 | | | | | | | | 0.0045 | | | | | | 0.0019 | | | | | | 0.0186 | | | | | | |  |  |  |  |  |  |  |  |  |  |  |  |  |  |
| 26 | 472 | | | | 156 | | | | | | | | | | 50 | | | | | | | | 12 | | | | | 3.09 | | | | | | | 4/686 | | | | | | | | 0.0043 | | | | | | 0.0019 | | | | | | 0.0274 | | | | | | |  |  |  |  |  |  |  |  |  |  |  |  |  |  |
| 27 | 474 | | | | 156 | | | | | | | | | | 50 | | | | | | | | 12 | | | | | 3.29 | | | | | | | 4/688 | | | | | | | | 0.0044 | | | | | | 0.0019 | | | | | | 0.0237 | | | | | | |  |  |  |  |  |  |  |  |  |  |  |  |  |  |
| 28 | 480 | | | | 156 | | | | | | | | | | 50 | | | | | | | | 12 | | | | | 3.28 | | | | | | | 4/694 | | | | | | | | 0.0045 | | | | | | 0.0019 | | | | | | 0.02 | | | | | | |  |  |  |  |  |  |  |  |  |  |  |  |  |  |
| 29 | 483 | | | | 157 | | | | | | | | | | 50 | | | | | | | | 12 | | | | | 3.24 | | | | | | | 4/698 | | | | | | | | 0.0043 | | | | | | 0.0019 | | | | | | 0.0223 | | | | | | |  |  |  |  |  |  |  |  |  |  |  |  |  |  |
| 30 | 483 | | | | 158 | | | | | | | | | | 50 | | | | | | | | 12 | | | | | 3.29 | | | | | | | 4/699 | | | | | | | | 0.0043 | | | | | | 0.0019 | | | | | | 0.0223 | | | | | | |  |  |  |  |  |  |  |  |  |  |  |  |  |  |
| 31 | 483 | | | | 158 | | | | | | | | | | 50 | | | | | | | | 12 | | | | | 3.27 | | | | | | | 4/699 | | | | | | | | 0.0045 | | | | | | 0.0019 | | | | | | 0.0203 | | | | | | |  |  |  |  |  |  |  |  |  |  |  |  |  |  |
| 32 | 485 | | | | 159 | | | | | | | | | | 50 | | | | | | | | 12 | | | | | 3.31 | | | | | | | 4/702 | | | | | | | | 0.0045 | | | | | | 0.002 | | | | | | 0.0203 | | | | | | |  |  |  |  |  |  |  |  |  |  |  |  |  |  |
| 33 | 486 | | | | 160 | | | | | | | | | | 50 | | | | | | | | 12 | | | | | 3.31 | | | | | | | 4/704 | | | | | | | | 0.0044 | | | | | | 0.002 | | | | | | 0.0243 | | | | | | |  |  |  |  |  |  |  |  |  |  |  |  |  |  |
| 34 | 489 | | | | 160 | | | | | | | | | | 50 | | | | | | | | 12 | | | | | 3.71 | | | | | | | 4/707 | | | | | | | | 0.0048 | | | | | | 0.002 | | | | | | 0.0153 | | | | | | |  |  |  |  |  |  |  |  |  |  |  |  |  |  |
| 35 | 493 | | | | 160 | | | | | | | | | | 50 | | | | | | | | 12 | | | | | 3.99 | | | | | | | 4/711 | | | | | | | | 0.0048 | | | | | | 0.0019 | | | | | | 0.0138 | | | | | | |  |  |  |  |  |  |  |  |  |  |  |  |  |  |
| 36 | 494 | | | | 160 | | | | | | | | | | 50 | | | | | | | | 12 | | | | | 4.02 | | | | | | | 4/712 | | | | | | | | 0.0048 | | | | | | 0.002 | | | | | | 0.0136 | | | | | | |  |  |  |  |  |  |  |  |  |  |  |  |  |  |
| 37 | 497 | | | | 160 | | | | | | | | | | 50 | | | | | | | | 12 | | | | | 4.08 | | | | | | | 4/715 | | | | | | | | 0.0049 | | | | | | 0.002 | | | | | | 0.0118 | | | | | | |  |  |  |  |  |  |  |  |  |  |  |  |  |  |
| 38 | 497 | | | | 160 | | | | | | | | | | 50 | | | | | | | | 12 | | | | | 4.27 | | | | | | | 4/715 | | | | | | | | 0.005 | | | | | | 0.002 | | | | | | 0.0121 | | | | | | |  |  |  |  |  |  |  |  |  |  |  |  |  |  |
| 39 | 501 | | | | 160 | | | | | | | | | | 50 | | | | | | | | 12 | | | | | 4.35 | | | | | | | 4/719 | | | | | | | | 0.0049 | | | | | | 0.002 | | | | | | 0.013 | | | | | | |  |  |  |  |  |  |  |  |  |  |  |  |  |  |
| 40 | 503 | | | | 161 | | | | | | | | | | 51 | | | | | | | | 12 | | | | | 4.42 | | | | | | | 4/723 | | | | | | | | 0.0051 | | | | | | 0.002 | | | | | | 0.0112 | | | | | | |  |  |  |  |  |  |  |  |  |  |  |  |  |  |
| 41 | 507 | | | | 162 | | | | | | | | | | 52 | | | | | | | | 12 | | | | | 4.13 | | | | | | | 4/729 | | | | | | | | 0.0052 | | | | | | 0.002 | | | | | | 0.0104 | | | | | | |  |  |  |  |  |  |  |  |  |  |  |  |  |  |
| 42 | 511 | | | | 162 | | | | | | | | | | 52 | | | | | | | | 12 | | | | | 4.32 | | | | | | | 4/733 | | | | | | | | 0.0054 | | | | | | 0.002 | | | | | | 0.00782 | | | | | | |  |  |  |  |  |  |  |  |  |  |  |  |  |  |
| 43 | 518 | | | | 162 | | | | | | | | | | 52 | | | | | | | | 12 | | | | | 4.55 | | | | | | | 4/740 | | | | | | | | 0.0055 | | | | | | 0.002 | | | | | | 0.00708 | | | | | | |  |  |  |  |  |  |  |  |  |  |  |  |  |  |
| 44 | 521 | | | | 162 | | | | | | | | | | 52 | | | | | | | | 12 | | | | | 4.52 | | | | | | | 4/743 | | | | | | | | 0.0056 | | | | | | 0.002 | | | | | | 0.00613 | | | | | | |  |  |  |  |  |  |  |  |  |  |  |  |  |  |
| 45 | 523 | | | | 162 | | | | | | | | | | 52 | | | | | | | | 12 | | | | | 4.62 | | | | | | | 4/745 | | | | | | | | 0.0055 | | | | | | 0.002 | | | | | | 0.00703 | | | | | | |  |  |  |  |  |  |  |  |  |  |  |  |  |  |
| 46 | 528 | | | | 162 | | | | | | | | | | 52 | | | | | | | | 12 | | | | | 4.5 | | | | | | | 4/750 | | | | | | | | 0.0055 | | | | | | 0.0021 | | | | | | 0.00752 | | | | | | |  |  |  |  |  |  |  |  |  |  |  |  |  |  |
| 47 | 531 | | | | 163 | | | | | | | | | | 52 | | | | | | | | 12 | | | | | 4.36 | | | | | | | 4/754 | | | | | | | | 0.0055 | | | | | | 0.0021 | | | | | | 0.00781 | | | | | | |  |  |  |  |  |  |  |  |  |  |  |  |  |  |
| 48 | 534 | | | | 163 | | | | | | | | | | 52 | | | | | | | | 12 | | | | | 4.4 | | | | | | | 4/757 | | | | | | | | 0.0055 | | | | | | 0.002 | | | | | | 0.00709 | | | | | | |  |  |  |  |  |  |  |  |  |  |  |  |  |  |
| 49 | 543 | | | | 164 | | | | | | | | | | 52 | | | | | | | | 12 | | | | | 4.11 | | | | | | | 4/767 | | | | | | | | 0.0056 | | | | | | 0.002 | | | | | | 0.00633 | | | | | | |  |  |  |  |  |  |  |  |  |  |  |  |  |  |
| 50 | 543 | | | | 164 | | | | | | | | | | 52 | | | | | | | | 12 | | | | | 4.11 | | | | | | | 4/767 | | | | | | | | 0.0056 | | | | | | 0.002 | | | | | | 0.00633 | | | | | | |  |  |  |  |  |  |  |  |  |  |  |  |  |  |
| **Table S2.** Predictive performance as a function of days from survey. | | | | | | | | | | | | | | | | | | | | | | | | | | | | | | | | | | | | | | | | | | | | | | | | | | | | | | | | | | | | | | | | | | | | |  |  |  |  |  |  |  |
| **Student Survey Cohort** | | | | | | | | | | | | | | | | | | | | | | | | | | | | | | | | | | | | | | | | | | | | | | | | | | | | | | | | | | | | | | | | | | | | |  |  |  |  |  |  |  |
| **Days Before Survey** | | | | | **N SI < 1** | | | | | | | **N SI > 1** | | | | **AUC** | | | | | | | **P value** | | | | | | **N SI < 2** | | | | | | **N SI > 2** | | | | | | | **AUC** | | | | | | **P value** | | | | **N SI < 3** | | | **N SI > 3** | | | **AUC** | | | | **P value** | | | | | | |  |  |  |  |  |  |  |
| 0 | | | | | 25 | | | | | | | 15 | | | | 0.58 | | | | | | | 0.204 | | | | | | 38 | | | | | | 2 | | | | | | | 0.75 | | | | | | 0.139 | | | | 39 | | | 1 | | | 0.72 | | | | 0.297 | | | | | | |  |  |  |  |  |  |  |
| 1 | | | | | 34 | | | | | | | 19 | | | | 0.6 | | | | | | | 0.111 | | | | | | 49 | | | | | | 4 | | | | | | | 0.58 | | | | | | 0.303 | | | | 50 | | | 3 | | | 0.52 | | | | 0.458 | | | | | | |  |  |  |  |  |  |  |
| 2 | | | | | 38 | | | | | | | 20 | | | | 0.58 | | | | | | | 0.165 | | | | | | 53 | | | | | | 5 | | | | | | | 0.63 | | | | | | 0.173 | | | | 54 | | | 4 | | | 0.58 | | | | 0.303 | | | | | | |  |  |  |  |  |  |  |
| 3 | | | | | 40 | | | | | | | 23 | | | | 0.53 | | | | | | | 0.344 | | | | | | 58 | | | | | | 5 | | | | | | | 0.52 | | | | | | 0.431 | | | | 59 | | | 4 | | | 0.44 | | | | 0.652 | | | | | | |  |  |  |  |  |  |  |
| 4 | | | | | 43 | | | | | | | 23 | | | | 0.53 | | | | | | | 0.366 | | | | | | 61 | | | | | | 5 | | | | | | | 0.52 | | | | | | 0.454 | | | | 62 | | | 4 | | | 0.49 | | | | 0.544 | | | | | | |  |  |  |  |  |  |  |
| 5 | | | | | 47 | | | | | | | 25 | | | | 0.54 | | | | | | | 0.292 | | | | | | 66 | | | | | | 6 | | | | | | | 0.53 | | | | | | 0.422 | | | | 67 | | | 5 | | | 0.56 | | | | 0.334 | | | | | | |  |  |  |  |  |  |  |
| 6 | | | | | 52 | | | | | | | 28 | | | | 0.54 | | | | | | | 0.309 | | | | | | 74 | | | | | | 6 | | | | | | | 0.5 | | | | | | 0.503 | | | | 75 | | | 5 | | | 0.54 | | | | 0.407 | | | | | | |  |  |  |  |  |  |  |
| 7 | | | | | 54 | | | | | | | 29 | | | | 0.52 | | | | | | | 0.388 | | | | | | 77 | | | | | | 6 | | | | | | | 0.52 | | | | | | 0.422 | | | | 78 | | | 5 | | | 0.57 | | | | 0.325 | | | | | | |  |  |  |  |  |  |  |
| 8 | | | | | 57 | | | | | | | 33 | | | | 0.57 | | | | | | | 0.147 | | | | | | 82 | | | | | | 8 | | | | | | | 0.69 | | | | | | 0.038 | | | | 85 | | | 5 | | | 0.64 | | | | 0.147 | | | | | | |  |  |  |  |  |  |  |
| 9 | | | | | 57 | | | | | | | 34 | | | | 0.52 | | | | | | | 0.381 | | | | | | 82 | | | | | | 9 | | | | | | | 0.57 | | | | | | 0.248 | | | | 86 | | | 5 | | | 0.65 | | | | 0.13 | | | | | | |  |  |  |  |  |  |  |
| 10 | | | | | 58 | | | | | | | 35 | | | | 0.56 | | | | | | | 0.176 | | | | | | 84 | | | | | | 9 | | | | | | | 0.57 | | | | | | 0.236 | | | | 88 | | | 5 | | | 0.67 | | | | 0.107 | | | | | | |  |  |  |  |  |  |  |
| 11 | | | | | 60 | | | | | | | 35 | | | | 0.62 | | | | | | | 0.025 | | | | | | 86 | | | | | | 9 | | | | | | | 0.63 | | | | | | 0.107 | | | | 90 | | | 5 | | | 0.68 | | | | 0.098 | | | | | | |  |  |  |  |  |  |  |
| 12 | | | | | 62 | | | | | | | 35 | | | | 0.59 | | | | | | | 0.07 | | | | | | 88 | | | | | | 9 | | | | | | | 0.6 | | | | | | 0.159 | | | | 92 | | | 5 | | | 0.67 | | | | 0.109 | | | | | | |  |  |  |  |  |  |  |
| 13 | | | | | 63 | | | | | | | 35 | | | | 0.6 | | | | | | | 0.053 | | | | | | 89 | | | | | | 9 | | | | | | | 0.62 | | | | | | 0.116 | | | | 93 | | | 5 | | | 0.67 | | | | 0.105 | | | | | | |  |  |  |  |  |  |  |
| 14 | | | | | 63 | | | | | | | 37 | | | | 0.58 | | | | | | | 0.088 | | | | | | 90 | | | | | | 10 | | | | | | | 0.65 | | | | | | 0.056 | | | | 94 | | | 6 | | | 0.71 | | | | 0.042 | | | | | | |  |  |  |  |  |  |  |
| 15 | | | | | 64 | | | | | | | 38 | | | | 0.58 | | | | | | | 0.102 | | | | | | 91 | | | | | | 11 | | | | | | | 0.6 | | | | | | 0.152 | | | | 96 | | | 6 | | | 0.72 | | | | 0.034 | | | | | | |  |  |  |  |  |  |  |
| 16 | | | | | 65 | | | | | | | 38 | | | | 0.57 | | | | | | | 0.112 | | | | | | 92 | | | | | | 11 | | | | | | | 0.58 | | | | | | 0.184 | | | | 97 | | | 6 | | | 0.7 | | | | 0.054 | | | | | | |  |  |  |  |  |  |  |
| 17 | | | | | 65 | | | | | | | 38 | | | | 0.59 | | | | | | | 0.072 | | | | | | 92 | | | | | | 11 | | | | | | | 0.61 | | | | | | 0.129 | | | | 97 | | | 6 | | | 0.73 | | | | 0.03 | | | | | | |  |  |  |  |  |  |  |
| 18 | | | | | 66 | | | | | | | 39 | | | | 0.59 | | | | | | | 0.062 | | | | | | 94 | | | | | | 11 | | | | | | | 0.59 | | | | | | 0.157 | | | | 99 | | | 6 | | | 0.7 | | | | 0.047 | | | | | | |  |  |  |  |  |  |  |
| 19 | | | | | 68 | | | | | | | 39 | | | | 0.59 | | | | | | | 0.052 | | | | | | 96 | | | | | | 11 | | | | | | | 0.58 | | | | | | 0.196 | | | | 101 | | | 6 | | | 0.68 | | | | 0.069 | | | | | | |  |  |  |  |  |  |  |
| 20 | | | | | 71 | | | | | | | 39 | | | | 0.58 | | | | | | | 0.097 | | | | | | 99 | | | | | | 11 | | | | | | | 0.57 | | | | | | 0.219 | | | | 104 | | | 6 | | | 0.68 | | | | 0.07 | | | | | | |  |  |  |  |  |  |  |
| 21 | | | | | 72 | | | | | | | 40 | | | | 0.54 | | | | | | | 0.24 | | | | | | 101 | | | | | | 11 | | | | | | | 0.57 | | | | | | 0.232 | | | | 106 | | | 6 | | | 0.67 | | | | 0.088 | | | | | | |  |  |  |  |  |  |  |
| 22 | | | | | 72 | | | | | | | 41 | | | | 0.55 | | | | | | | 0.199 | | | | | | 102 | | | | | | 11 | | | | | | | 0.57 | | | | | | 0.225 | | | | 107 | | | 6 | | | 0.67 | | | | 0.089 | | | | | | |  |  |  |  |  |  |  |
| 23 | | | | | 73 | | | | | | | 42 | | | | 0.56 | | | | | | | 0.155 | | | | | | 104 | | | | | | 11 | | | | | | | 0.58 | | | | | | 0.19 | | | | 109 | | | 6 | | | 0.68 | | | | 0.073 | | | | | | |  |  |  |  |  |  |  |
| 24 | | | | | 73 | | | | | | | 43 | | | | 0.53 | | | | | | | 0.27 | | | | | | 104 | | | | | | 12 | | | | | | | 0.55 | | | | | | 0.298 | | | | 110 | | | 6 | | | 0.69 | | | | 0.061 | | | | | | |  |  |  |  |  |  |  |
| 25 | | | | | 73 | | | | | | | 43 | | | | 0.55 | | | | | | | 0.204 | | | | | | 104 | | | | | | 12 | | | | | | | 0.55 | | | | | | 0.28 | | | | 110 | | | 6 | | | 0.68 | | | | 0.071 | | | | | | |  |  |  |  |  |  |  |
| 26 | | | | | 75 | | | | | | | 43 | | | | 0.56 | | | | | | | 0.124 | | | | | | 106 | | | | | | 12 | | | | | | | 0.58 | | | | | | 0.173 | | | | 112 | | | 6 | | | 0.71 | | | | 0.038 | | | | | | |  |  |  |  |  |  |  |
| 27 | | | | | 77 | | | | | | | 44 | | | | 0.58 | | | | | | | 0.064 | | | | | | 108 | | | | | | 13 | | | | | | | 0.61 | | | | | | 0.093 | | | | 115 | | | 6 | | | 0.71 | | | | 0.043 | | | | | | |  |  |  |  |  |  |  |
| 28 | | | | | 77 | | | | | | | 44 | | | | 0.59 | | | | | | | 0.046 | | | | | | 108 | | | | | | 13 | | | | | | | 0.62 | | | | | | 0.077 | | | | 115 | | | 6 | | | 0.72 | | | | 0.033 | | | | | | |  |  |  |  |  |  |  |
| 29 | | | | | 77 | | | | | | | 44 | | | | 0.58 | | | | | | | 0.075 | | | | | | 108 | | | | | | 13 | | | | | | | 0.62 | | | | | | 0.088 | | | | 115 | | | 6 | | | 0.72 | | | | 0.036 | | | | | | |  |  |  |  |  |  |  |
| 30 | | | | | 78 | | | | | | | 44 | | | | 0.56 | | | | | | | 0.124 | | | | | | 109 | | | | | | 13 | | | | | | | 0.59 | | | | | | 0.134 | | | | 116 | | | 6 | | | 0.68 | | | | 0.065 | | | | | | |  |  |  |  |  |  |  |
| 31 | | | | | 78 | | | | | | | 45 | | | | 0.56 | | | | | | | 0.117 | | | | | | 110 | | | | | | 13 | | | | | | | 0.59 | | | | | | 0.145 | | | | 117 | | | 6 | | | 0.68 | | | | 0.066 | | | | | | |  |  |  |  |  |  |  |
| 32 | | | | | 78 | | | | | | | 45 | | | | 0.55 | | | | | | | 0.158 | | | | | | 110 | | | | | | 13 | | | | | | | 0.59 | | | | | | 0.158 | | | | 117 | | | 6 | | | 0.67 | | | | 0.08 | | | | | | |  |  |  |  |  |  |  |
| 33 | | | | | 78 | | | | | | | 46 | | | | 0.56 | | | | | | | 0.149 | | | | | | 111 | | | | | | 13 | | | | | | | 0.59 | | | | | | 0.137 | | | | 118 | | | 6 | | | 0.68 | | | | 0.073 | | | | | | |  |  |  |  |  |  |  |
| 34 | | | | | 78 | | | | | | | 47 | | | | 0.57 | | | | | | | 0.112 | | | | | | 112 | | | | | | 13 | | | | | | | 0.59 | | | | | | 0.143 | | | | 119 | | | 6 | | | 0.68 | | | | 0.068 | | | | | | |  |  |  |  |  |  |  |
| 35 | | | | | 79 | | | | | | | 47 | | | | 0.58 | | | | | | | 0.074 | | | | | | 113 | | | | | | 13 | | | | | | | 0.6 | | | | | | 0.117 | | | | 120 | | | 6 | | | 0.7 | | | | 0.051 | | | | | | |  |  |  |  |  |  |  |
| 36 | | | | | 80 | | | | | | | 47 | | | | 0.58 | | | | | | | 0.059 | | | | | | 114 | | | | | | 13 | | | | | | | 0.59 | | | | | | 0.145 | | | | 121 | | | 6 | | | 0.69 | | | | 0.056 | | | | | | |  |  |  |  |  |  |  |
| 37 | | | | | 80 | | | | | | | 47 | | | | 0.59 | | | | | | | 0.042 | | | | | | 114 | | | | | | 13 | | | | | | | 0.59 | | | | | | 0.133 | | | | 121 | | | 6 | | | 0.7 | | | | 0.055 | | | | | | |  |  |  |  |  |  |  |
| 38 | | | | | 81 | | | | | | | 47 | | | | 0.6 | | | | | | | 0.03 | | | | | | 115 | | | | | | 13 | | | | | | | 0.6 | | | | | | 0.119 | | | | 122 | | | 6 | | | 0.7 | | | | 0.043 | | | | | | |  |  |  |  |  |  |  |
| 39 | | | | | 81 | | | | | | | 47 | | | | 0.6 | | | | | | | 0.033 | | | | | | 115 | | | | | | 13 | | | | | | | 0.59 | | | | | | 0.149 | | | | 122 | | | 6 | | | 0.68 | | | | 0.066 | | | | | | |  |  |  |  |  |  |  |
| 40 | | | | | 82 | | | | | | | 47 | | | | 0.6 | | | | | | | 0.034 | | | | | | 116 | | | | | | 13 | | | | | | | 0.59 | | | | | | 0.146 | | | | 123 | | | 6 | | | 0.68 | | | | 0.069 | | | | | | |  |  |  |  |  |  |  |
| 41 | | | | | 82 | | | | | | | 47 | | | | 0.59 | | | | | | | 0.045 | | | | | | 116 | | | | | | 13 | | | | | | | 0.6 | | | | | | 0.131 | | | | 123 | | | 6 | | | 0.69 | | | | 0.062 | | | | | | |  |  |  |  |  |  |  |
| 42 | | | | | 83 | | | | | | | 47 | | | | 0.6 | | | | | | | 0.029 | | | | | | 117 | | | | | | 13 | | | | | | | 0.6 | | | | | | 0.118 | | | | 124 | | | 6 | | | 0.69 | | | | 0.056 | | | | | | |  |  |  |  |  |  |  |
| 43 | | | | | 85 | | | | | | | 47 | | | | 0.62 | | | | | | | 0.013 | | | | | | 119 | | | | | | 13 | | | | | | | 0.61 | | | | | | 0.104 | | | | 126 | | | 6 | | | 0.7 | | | | 0.045 | | | | | | |  |  |  |  |  |  |  |
| 44 | | | | | 87 | | | | | | | 47 | | | | 0.63 | | | | | | | 0.008 | | | | | | 121 | | | | | | 13 | | | | | | | 0.61 | | | | | | 0.093 | | | | 128 | | | 6 | | | 0.7 | | | | 0.051 | | | | | | |  |  |  |  |  |  |  |
| 45 | | | | | 89 | | | | | | | 47 | | | | 0.62 | | | | | | | 0.012 | | | | | | 123 | | | | | | 13 | | | | | | | 0.6 | | | | | | 0.124 | | | | 130 | | | 6 | | | 0.67 | | | | 0.078 | | | | | | |  |  |  |  |  |  |  |
| 46 | | | | | 89 | | | | | | | 47 | | | | 0.62 | | | | | | | 0.01 | | | | | | 123 | | | | | | 13 | | | | | | | 0.61 | | | | | | 0.099 | | | | 130 | | | 6 | | | 0.68 | | | | 0.073 | | | | | | |  |  |  |  |  |  |  |
| 47 | | | | | 90 | | | | | | | 47 | | | | 0.61 | | | | | | | 0.017 | | | | | | 124 | | | | | | 13 | | | | | | | 0.61 | | | | | | 0.097 | | | | 131 | | | 6 | | | 0.69 | | | | 0.06 | | | | | | |  |  |  |  |  |  |  |
| 48 | | | | | 92 | | | | | | | 48 | | | | 0.6 | | | | | | | 0.033 | | | | | | 127 | | | | | | 13 | | | | | | | 0.59 | | | | | | 0.128 | | | | 134 | | | 6 | | | 0.68 | | | | 0.075 | | | | | | |  |  |  |  |  |  |  |
| 49 | | | | | 93 | | | | | | | 48 | | | | 0.59 | | | | | | | 0.037 | | | | | | 128 | | | | | | 13 | | | | | | | 0.59 | | | | | | 0.151 | | | | 135 | | | 6 | | | 0.68 | | | | 0.074 | | | | | | |  |  |  |  |  |  |  |
| 50 | | | | | 93 | | | | | | | 48 | | | | 0.59 | | | | | | | 0.038 | | | | | | 128 | | | | | | 13 | | | | | | | 0.59 | | | | | | 0.16 | | | | 135 | | | 6 | | | 0.67 | | | | 0.084 | | | | | | |  |  |  |  |  |  |  |
| **COVID-19 Survey Cohort** | | | | | | | | | | | | | | | | | | | | | | | | | | | | | | | | | | | | | | | | | | | | | | | | | | | | | | | | | | | | | | | | | | | | |  |  |  |  |  |  |  |
| **Days Before Survey** | | | | | **N SI < 1** | | | | | | | | | **N SI > 1** | | | | | | | **AUC** | | | | | **P value** | | | **N SI < 2** | | | | | | **N SI > 2** | | | | | | | **AUC** | | | | | | **P value** | | | | **N SI < 3** | | | **N SI > 3** | | | **AUC** | | | | **P value** | | | | | | |  |  |  |  |  |  |  |
| 2 | | | | | 287 | | | | | | | | | 71 | | | | | | | 0.65 | | | | | 0 | | | 341 | | | | | | 17 | | | | | | | 0.53 | | | | | | 0.335 | | | | 357 | | | 1 | | | 0.98 | | | | 0.048 | | | | | | |  |  |  |  |  |  |  |
| 3 | | | | | 301 | | | | | | | | | 78 | | | | | | | 0.59 | | | | | 0.005 | | | 358 | | | | | | 21 | | | | | | | 0.54 | | | | | | 0.254 | | | | 378 | | | 1 | | | 0.98 | | | | 0.033 | | | | | | |  |  |  |  |  |  |  |
| 4 | | | | | 318 | | | | | | | | | 86 | | | | | | | 0.56 | | | | | 0.042 | | | 378 | | | | | | 26 | | | | | | | 0.51 | | | | | | 0.412 | | | | 401 | | | 3 | | | 0.69 | | | | 0.143 | | | | | | |  |  |  |  |  |  |  |
| 5 | | | | | 330 | | | | | | | | | 88 | | | | | | | 0.55 | | | | | 0.067 | | | 390 | | | | | | 28 | | | | | | | 0.52 | | | | | | 0.394 | | | | 415 | | | 3 | | | 0.61 | | | | 0.266 | | | | | | |  |  |  |  |  |  |  |
| 6 | | | | | 342 | | | | | | | | | 90 | | | | | | | 0.54 | | | | | 0.104 | | | 403 | | | | | | 29 | | | | | | | 0.51 | | | | | | 0.418 | | | | 429 | | | 3 | | | 0.59 | | | | 0.304 | | | | | | |  |  |  |  |  |  |  |
| 7 | | | | | 361 | | | | | | | | | 90 | | | | | | | 0.52 | | | | | 0.273 | | | 422 | | | | | | 29 | | | | | | | 0.46 | | | | | | 0.734 | | | | 448 | | | 3 | | | 0.59 | | | | 0.297 | | | | | | |  |  |  |  |  |  |  |
| 8 | | | | | 370 | | | | | | | | | 91 | | | | | | | 0.53 | | | | | 0.198 | | | 432 | | | | | | 29 | | | | | | | 0.45 | | | | | | 0.8 | | | | 458 | | | 3 | | | 0.6 | | | | 0.291 | | | | | | |  |  |  |  |  |  |  |
| 9 | | | | | 379 | | | | | | | | | 94 | | | | | | | 0.55 | | | | | 0.067 | | | 444 | | | | | | 29 | | | | | | | 0.47 | | | | | | 0.712 | | | | 470 | | | 3 | | | 0.66 | | | | 0.171 | | | | | | |  |  |  |  |  |  |  |
| 10 | | | | | 385 | | | | | | | | | 97 | | | | | | | 0.54 | | | | | 0.122 | | | 452 | | | | | | 30 | | | | | | | 0.46 | | | | | | 0.769 | | | | 479 | | | 3 | | | 0.67 | | | | 0.16 | | | | | | |  |  |  |  |  |  |  |
| 11 | | | | | 391 | | | | | | | | | 97 | | | | | | | 0.55 | | | | | 0.08 | | | 458 | | | | | | 30 | | | | | | | 0.47 | | | | | | 0.701 | | | | 485 | | | 3 | | | 0.84 | | | | 0.018 | | | | | | |  |  |  |  |  |  |  |
| 12 | | | | | 399 | | | | | | | | | 100 | | | | | | | 0.56 | | | | | 0.027 | | | 467 | | | | | | 32 | | | | | | | 0.5 | | | | | | 0.467 | | | | 496 | | | 3 | | | 0.84 | | | | 0.021 | | | | | | |  |  |  |  |  |  |  |
| 13 | | | | | 402 | | | | | | | | | 101 | | | | | | | 0.56 | | | | | 0.037 | | | 470 | | | | | | 33 | | | | | | | 0.52 | | | | | | 0.366 | | | | 499 | | | 4 | | | 0.88 | | | | 0.002 | | | | | | |  |  |  |  |  |  |  |
| 14 | | | | | 409 | | | | | | | | | 101 | | | | | | | 0.57 | | | | | 0.016 | | | 477 | | | | | | 33 | | | | | | | 0.55 | | | | | | 0.163 | | | | 506 | | | 4 | | | 0.87 | | | | 0.004 | | | | | | |  |  |  |  |  |  |  |
| 15 | | | | | 416 | | | | | | | | | 103 | | | | | | | 0.56 | | | | | 0.024 | | | 486 | | | | | | 33 | | | | | | | 0.53 | | | | | | 0.313 | | | | 515 | | | 4 | | | 0.66 | | | | 0.147 | | | | | | |  |  |  |  |  |  |  |
| 16 | | | | | 423 | | | | | | | | | 104 | | | | | | | 0.55 | | | | | 0.052 | | | 494 | | | | | | 33 | | | | | | | 0.53 | | | | | | 0.287 | | | | 523 | | | 4 | | | 0.71 | | | | 0.07 | | | | | | |  |  |  |  |  |  |  |
| 17 | | | | | 427 | | | | | | | | | 104 | | | | | | | 0.55 | | | | | 0.051 | | | 498 | | | | | | 33 | | | | | | | 0.52 | | | | | | 0.376 | | | | 527 | | | 4 | | | 0.63 | | | | 0.201 | | | | | | |  |  |  |  |  |  |  |
| 18 | | | | | 429 | | | | | | | | | 105 | | | | | | | 0.54 | | | | | 0.09 | | | 501 | | | | | | 33 | | | | | | | 0.51 | | | | | | 0.418 | | | | 530 | | | 4 | | | 0.63 | | | | 0.198 | | | | | | |  |  |  |  |  |  |  |
| 19 | | | | | 435 | | | | | | | | | 106 | | | | | | | 0.54 | | | | | 0.133 | | | 508 | | | | | | 33 | | | | | | | 0.51 | | | | | | 0.452 | | | | 537 | | | 4 | | | 0.55 | | | | 0.351 | | | | | | |  |  |  |  |  |  |  |
| 20 | | | | | 439 | | | | | | | | | 107 | | | | | | | 0.53 | | | | | 0.198 | | | 513 | | | | | | 33 | | | | | | | 0.5 | | | | | | 0.529 | | | | 542 | | | 4 | | | 0.55 | | | | 0.367 | | | | | | |  |  |  |  |  |  |  |
| 21 | | | | | 444 | | | | | | | | | 107 | | | | | | | 0.53 | | | | | 0.167 | | | 518 | | | | | | 33 | | | | | | | 0.5 | | | | | | 0.477 | | | | 547 | | | 4 | | | 0.55 | | | | 0.355 | | | | | | |  |  |  |  |  |  |  |
| 22 | | | | | 447 | | | | | | | | | 109 | | | | | | | 0.54 | | | | | 0.084 | | | 522 | | | | | | 34 | | | | | | | 0.52 | | | | | | 0.385 | | | | 552 | | | 4 | | | 0.53 | | | | 0.42 | | | | | | |  |  |  |  |  |  |  |
| 23 | | | | | 450 | | | | | | | | | 110 | | | | | | | 0.54 | | | | | 0.077 | | | 526 | | | | | | 34 | | | | | | | 0.51 | | | | | | 0.447 | | | | 556 | | | 4 | | | 0.5 | | | | 0.509 | | | | | | |  |  |  |  |  |  |  |
| 24 | | | | | 456 | | | | | | | | | 112 | | | | | | | 0.54 | | | | | 0.093 | | | 532 | | | | | | 36 | | | | | | | 0.52 | | | | | | 0.333 | | | | 563 | | | 5 | | | 0.66 | | | | 0.124 | | | | | | |  |  |  |  |  |  |  |
| 25 | | | | | 457 | | | | | | | | | 113 | | | | | | | 0.54 | | | | | 0.11 | | | 534 | | | | | | 36 | | | | | | | 0.52 | | | | | | 0.377 | | | | 565 | | | 5 | | | 0.69 | | | | 0.072 | | | | | | |  |  |  |  |  |  |  |
| 26 | | | | | 465 | | | | | | | | | 114 | | | | | | | 0.53 | | | | | 0.128 | | | 542 | | | | | | 37 | | | | | | | 0.51 | | | | | | 0.45 | | | | 574 | | | 5 | | | 0.68 | | | | 0.084 | | | | | | |  |  |  |  |  |  |  |
| 27 | | | | | 469 | | | | | | | | | 115 | | | | | | | 0.54 | | | | | 0.112 | | | 546 | | | | | | 38 | | | | | | | 0.51 | | | | | | 0.391 | | | | 578 | | | 6 | | | 0.71 | | | | 0.032 | | | | | | |  |  |  |  |  |  |  |
| 28 | | | | | 472 | | | | | | | | | 115 | | | | | | | 0.54 | | | | | 0.084 | | | 549 | | | | | | 38 | | | | | | | 0.53 | | | | | | 0.3 | | | | 581 | | | 6 | | | 0.72 | | | | 0.033 | | | | | | |  |  |  |  |  |  |  |
| 29 | | | | | 478 | | | | | | | | | 115 | | | | | | | 0.55 | | | | | 0.063 | | | 555 | | | | | | 38 | | | | | | | 0.53 | | | | | | 0.298 | | | | 587 | | | 6 | | | 0.71 | | | | 0.042 | | | | | | |  |  |  |  |  |  |  |
| 30 | | | | | 480 | | | | | | | | | 117 | | | | | | | 0.55 | | | | | 0.049 | | | 559 | | | | | | 38 | | | | | | | 0.52 | | | | | | 0.348 | | | | 591 | | | 6 | | | 0.72 | | | | 0.03 | | | | | | |  |  |  |  |  |  |  |
| 31 | | | | | 480 | | | | | | | | | 117 | | | | | | | 0.55 | | | | | 0.053 | | | 559 | | | | | | 38 | | | | | | | 0.51 | | | | | | 0.398 | | | | 591 | | | 6 | | | 0.72 | | | | 0.028 | | | | | | |  |  |  |  |  |  |  |
| 32 | | | | | 480 | | | | | | | | | 117 | | | | | | | 0.55 | | | | | 0.043 | | | 559 | | | | | | 38 | | | | | | | 0.51 | | | | | | 0.404 | | | | 591 | | | 6 | | | 0.73 | | | | 0.028 | | | | | | |  |  |  |  |  |  |  |
| 33 | | | | | 482 | | | | | | | | | 117 | | | | | | | 0.55 | | | | | 0.054 | | | 561 | | | | | | 38 | | | | | | | 0.51 | | | | | | 0.404 | | | | 593 | | | 6 | | | 0.73 | | | | 0.026 | | | | | | |  |  |  |  |  |  |  |
| 34 | | | | | 484 | | | | | | | | | 118 | | | | | | | 0.55 | | | | | 0.059 | | | 563 | | | | | | 39 | | | | | | | 0.51 | | | | | | 0.444 | | | | 596 | | | 6 | | | 0.71 | | | | 0.036 | | | | | | |  |  |  |  |  |  |  |
| 35 | | | | | 486 | | | | | | | | | 118 | | | | | | | 0.55 | | | | | 0.063 | | | 565 | | | | | | 39 | | | | | | | 0.52 | | | | | | 0.361 | | | | 598 | | | 6 | | | 0.71 | | | | 0.034 | | | | | | |  |  |  |  |  |  |  |
| 36 | | | | | 489 | | | | | | | | | 118 | | | | | | | 0.55 | | | | | 0.043 | | | 568 | | | | | | 39 | | | | | | | 0.53 | | | | | | 0.285 | | | | 601 | | | 6 | | | 0.72 | | | | 0.032 | | | | | | |  |  |  |  |  |  |  |
| 37 | | | | | 491 | | | | | | | | | 118 | | | | | | | 0.55 | | | | | 0.051 | | | 570 | | | | | | 39 | | | | | | | 0.52 | | | | | | 0.317 | | | | 603 | | | 6 | | | 0.71 | | | | 0.04 | | | | | | |  |  |  |  |  |  |  |
| 38 | | | | | 494 | | | | | | | | | 118 | | | | | | | 0.55 | | | | | 0.038 | | | 573 | | | | | | 39 | | | | | | | 0.53 | | | | | | 0.291 | | | | 606 | | | 6 | | | 0.7 | | | | 0.042 | | | | | | |  |  |  |  |  |  |  |
| 39 | | | | | 494 | | | | | | | | | 118 | | | | | | | 0.56 | | | | | 0.023 | | | 573 | | | | | | 39 | | | | | | | 0.54 | | | | | | 0.208 | | | | 606 | | | 6 | | | 0.7 | | | | 0.042 | | | | | | |  |  |  |  |  |  |  |
| 40 | | | | | 498 | | | | | | | | | 118 | | | | | | | 0.56 | | | | | 0.029 | | | 577 | | | | | | 39 | | | | | | | 0.54 | | | | | | 0.201 | | | | 610 | | | 6 | | | 0.71 | | | | 0.04 | | | | | | |  |  |  |  |  |  |  |
| 41 | | | | | 501 | | | | | | | | | 119 | | | | | | | 0.55 | | | | | 0.031 | | | 580 | | | | | | 40 | | | | | | | 0.53 | | | | | | 0.248 | | | | 614 | | | 6 | | | 0.7 | | | | 0.045 | | | | | | |  |  |  |  |  |  |  |
| 42 | | | | | 505 | | | | | | | | | 120 | | | | | | | 0.55 | | | | | 0.041 | | | 584 | | | | | | 41 | | | | | | | 0.52 | | | | | | 0.312 | | | | 619 | | | 6 | | | 0.7 | | | | 0.045 | | | | | | |  |  |  |  |  |  |  |
| 43 | | | | | 508 | | | | | | | | | 120 | | | | | | | 0.56 | | | | | 0.024 | | | 587 | | | | | | 41 | | | | | | | 0.52 | | | | | | 0.325 | | | | 622 | | | 6 | | | 0.67 | | | | 0.069 | | | | | | |  |  |  |  |  |  |  |
| 44 | | | | | 513 | | | | | | | | | 121 | | | | | | | 0.56 | | | | | 0.017 | | | 593 | | | | | | 41 | | | | | | | 0.52 | | | | | | 0.308 | | | | 628 | | | 6 | | | 0.67 | | | | 0.074 | | | | | | |  |  |  |  |  |  |  |
| 45 | | | | | 514 | | | | | | | | | 121 | | | | | | | 0.56 | | | | | 0.013 | | | 594 | | | | | | 41 | | | | | | | 0.52 | | | | | | 0.295 | | | | 629 | | | 6 | | | 0.7 | | | | 0.054 | | | | | | |  |  |  |  |  |  |  |
| 46 | | | | | 516 | | | | | | | | | 121 | | | | | | | 0.57 | | | | | 0.012 | | | 596 | | | | | | 41 | | | | | | | 0.52 | | | | | | 0.35 | | | | 631 | | | 6 | | | 0.67 | | | | 0.078 | | | | | | |  |  |  |  |  |  |  |
| 47 | | | | | 520 | | | | | | | | | 121 | | | | | | | 0.56 | | | | | 0.013 | | | 600 | | | | | | 41 | | | | | | | 0.52 | | | | | | 0.349 | | | | 635 | | | 6 | | | 0.67 | | | | 0.074 | | | | | | |  |  |  |  |  |  |  |
| 48 | | | | | 521 | | | | | | | | | 121 | | | | | | | 0.57 | | | | | 0.009 | | | 601 | | | | | | 41 | | | | | | | 0.52 | | | | | | 0.317 | | | | 636 | | | 6 | | | 0.67 | | | | 0.081 | | | | | | |  |  |  |  |  |  |  |
| 49 | | | | | 523 | | | | | | | | | 121 | | | | | | | 0.57 | | | | | 0.009 | | | 603 | | | | | | 41 | | | | | | | 0.53 | | | | | | 0.276 | | | | 638 | | | 6 | | | 0.69 | | | | 0.06 | | | | | | |  |  |  |  |  |  |  |
| 50 | | | | | 532 | | | | | | | | | 122 | | | | | | | 0.56 | | | | | 0.018 | | | 613 | | | | | | 41 | | | | | | | 0.52 | | | | | | 0.336 | | | | 648 | | | 6 | | | 0.65 | | | | 0.103 | | | | | | |  |  |  |  |  |  |  |
| **Combined Cohort** | | | | | | | | | | | | | | | | | | | | | | | | | | | | | | | | | | | | | | | | | | | | | | | | | | | | | | | | | | | | | | | | | | | | |  |  |  |  |  |  |  |
| **Days Before Survey** | | | | | | **N SI < 1** | | | | | | | | **N SI > 1** | | | | | | | **AUC** | | | | | **P value** | | | | | **N SI < 2** | | | | | | | | **N SI > 2** | | | **AUC** | | | | | | | **P value** | | | **N SI < 3** | | | **N SI > 3** | | | **AUC** | | | | **P value** | | | | | | |  |  |  |  |  |  |  |
| 0 | | | | | | 212 | | | | | | | | 65 | | | | | | | 0.63 | | | | | 6E-04 | | | | | 264 | | | | | | | | 13 | | | 0.61 | | | | | | | 0.104 | | | 276 | | | 1 | | | 0.74 | | | | 0.263 | | | | | | |  |  |  |  |  |  |  |
| 1 | | | | | | 282 | | | | | | | | 82 | | | | | | | 0.61 | | | | | 8E-04 | | | | | 345 | | | | | | | | 19 | | | 0.51 | | | | | | | 0.476 | | | 361 | | | 3 | | | 0.54 | | | | 0.414 | | | | | | |  |  |  |  |  |  |  |
| 2 | | | | | | 325 | | | | | | | | 91 | | | | | | | 0.63 | | | | | 0 | | | | | 394 | | | | | | | | 22 | | | 0.56 | | | | | | | 0.178 | | | 411 | | | 5 | | | 0.67 | | | | 0.101 | | | | | | |  |  |  |  |  |  |  |
| 3 | | | | | | 341 | | | | | | | | 101 | | | | | | | 0.58 | | | | | 0.006 | | | | | 416 | | | | | | | | 26 | | | 0.55 | | | | | | | 0.222 | | | 437 | | | 5 | | | 0.57 | | | | 0.303 | | | | | | |  |  |  |  |  |  |  |
| 4 | | | | | | 361 | | | | | | | | 109 | | | | | | | 0.55 | | | | | 0.044 | | | | | 439 | | | | | | | | 31 | | | 0.51 | | | | | | | 0.4 | | | 463 | | | 7 | | | 0.57 | | | | 0.277 | | | | | | |  |  |  |  |  |  |  |
| 5 | | | | | | 377 | | | | | | | | 113 | | | | | | | 0.55 | | | | | 0.048 | | | | | 456 | | | | | | | | 34 | | | 0.52 | | | | | | | 0.372 | | | 482 | | | 8 | | | 0.58 | | | | 0.211 | | | | | | |  |  |  |  |  |  |  |
| 6 | | | | | | 394 | | | | | | | | 118 | | | | | | | 0.55 | | | | | 0.065 | | | | | 477 | | | | | | | | 35 | | | 0.51 | | | | | | | 0.401 | | | 504 | | | 8 | | | 0.58 | | | | 0.216 | | | | | | |  |  |  |  |  |  |  |
| 7 | | | | | | 415 | | | | | | | | 119 | | | | | | | 0.52 | | | | | 0.218 | | | | | 499 | | | | | | | | 35 | | | 0.48 | | | | | | | 0.68 | | | 526 | | | 8 | | | 0.59 | | | | 0.205 | | | | | | |  |  |  |  |  |  |  |
| 8 | | | | | | 427 | | | | | | | | 124 | | | | | | | 0.54 | | | | | 0.077 | | | | | 514 | | | | | | | | 37 | | | 0.51 | | | | | | | 0.445 | | | 543 | | | 8 | | | 0.65 | | | | 0.079 | | | | | | |  |  |  |  |  |  |  |
| 9 | | | | | | 436 | | | | | | | | 128 | | | | | | | 0.55 | | | | | 0.043 | | | | | 526 | | | | | | | | 38 | | | 0.5 | | | | | | | 0.545 | | | 556 | | | 8 | | | 0.67 | | | | 0.047 | | | | | | |  |  |  |  |  |  |  |
| 10 | | | | | | 443 | | | | | | | | 132 | | | | | | | 0.55 | | | | | 0.041 | | | | | 536 | | | | | | | | 39 | | | 0.49 | | | | | | | 0.59 | | | 567 | | | 8 | | | 0.69 | | | | 0.036 | | | | | | |  |  |  |  |  |  |  |
| 11 | | | | | | 451 | | | | | | | | 132 | | | | | | | 0.57 | | | | | 0.008 | | | | | 544 | | | | | | | | 39 | | | 0.51 | | | | | | | 0.409 | | | 575 | | | 8 | | | 0.76 | | | | 0.005 | | | | | | |  |  |  |  |  |  |  |
| 12 | | | | | | 461 | | | | | | | | 135 | | | | | | | 0.58 | | | | | 0.003 | | | | | 555 | | | | | | | | 41 | | | 0.53 | | | | | | | 0.269 | | | 588 | | | 8 | | | 0.76 | | | | 0.005 | | | | | | |  |  |  |  |  |  |  |
| 13 | | | | | | 465 | | | | | | | | 136 | | | | | | | 0.58 | | | | | 0.003 | | | | | 559 | | | | | | | | 42 | | | 0.55 | | | | | | | 0.159 | | | 592 | | | 9 | | | 0.79 | | | | 0.002 | | | | | | |  |  |  |  |  |  |  |
| 14 | | | | | | 472 | | | | | | | | 138 | | | | | | | 0.58 | | | | | 0.002 | | | | | 567 | | | | | | | | 43 | | | 0.58 | | | | | | | 0.042 | | | 600 | | | 10 | | | 0.8 | | | | 3E-04 | | | | | | |  |  |  |  |  |  |  |
| 15 | | | | | | 480 | | | | | | | | 141 | | | | | | | 0.58 | | | | | 0.003 | | | | | 577 | | | | | | | | 44 | | | 0.55 | | | | | | | 0.159 | | | 611 | | | 10 | | | 0.71 | | | | 0.01 | | | | | | |  |  |  |  |  |  |  |
| 16 | | | | | | 488 | | | | | | | | 142 | | | | | | | 0.57 | | | | | 0.008 | | | | | 586 | | | | | | | | 44 | | | 0.55 | | | | | | | 0.146 | | | 620 | | | 10 | | | 0.73 | | | | 0.005 | | | | | | |  |  |  |  |  |  |  |
| 17 | | | | | | 492 | | | | | | | | 142 | | | | | | | 0.57 | | | | | 0.007 | | | | | 590 | | | | | | | | 44 | | | 0.54 | | | | | | | 0.194 | | | 624 | | | 10 | | | 0.7 | | | | 0.014 | | | | | | |  |  |  |  |  |  |  |
| 18 | | | | | | 495 | | | | | | | | 144 | | | | | | | 0.56 | | | | | 0.013 | | | | | 595 | | | | | | | | 44 | | | 0.53 | | | | | | | 0.229 | | | 629 | | | 10 | | | 0.69 | | | | 0.019 | | | | | | |  |  |  |  |  |  |  |
| 19 | | | | | | 503 | | | | | | | | 145 | | | | | | | 0.56 | | | | | 0.016 | | | | | 604 | | | | | | | | 44 | | | 0.53 | | | | | | | 0.261 | | | 638 | | | 10 | | | 0.66 | | | | 0.044 | | | | | | |  |  |  |  |  |  |  |
| 20 | | | | | | 510 | | | | | | | | 146 | | | | | | | 0.55 | | | | | 0.043 | | | | | 612 | | | | | | | | 44 | | | 0.52 | | | | | | | 0.341 | | | 646 | | | 10 | | | 0.65 | | | | 0.053 | | | | | | |  |  |  |  |  |  |  |
| 21 | | | | | | 516 | | | | | | | | 147 | | | | | | | 0.54 | | | | | 0.054 | | | | | 619 | | | | | | | | 44 | | | 0.53 | | | | | | | 0.284 | | | 653 | | | 10 | | | 0.65 | | | | 0.054 | | | | | | |  |  |  |  |  |  |  |
| 22 | | | | | | 519 | | | | | | | | 150 | | | | | | | 0.55 | | | | | 0.02 | | | | | 624 | | | | | | | | 45 | | | 0.53 | | | | | | | 0.221 | | | 659 | | | 10 | | | 0.64 | | | | 0.064 | | | | | | |  |  |  |  |  |  |  |
| 23 | | | | | | 523 | | | | | | | | 152 | | | | | | | 0.56 | | | | | 0.019 | | | | | 630 | | | | | | | | 45 | | | 0.53 | | | | | | | 0.254 | | | 665 | | | 10 | | | 0.63 | | | | 0.077 | | | | | | |  |  |  |  |  |  |  |
| 24 | | | | | | 529 | | | | | | | | 155 | | | | | | | 0.55 | | | | | 0.037 | | | | | 636 | | | | | | | | 48 | | | 0.53 | | | | | | | 0.252 | | | 673 | | | 11 | | | 0.69 | | | | 0.014 | | | | | | |  |  |  |  |  |  |  |
| 25 | | | | | | 530 | | | | | | | | 156 | | | | | | | 0.55 | | | | | 0.033 | | | | | 638 | | | | | | | | 48 | | | 0.53 | | | | | | | 0.246 | | | 675 | | | 11 | | | 0.71 | | | | 0.01 | | | | | | |  |  |  |  |  |  |  |
| 26 | | | | | | 540 | | | | | | | | 157 | | | | | | | 0.55 | | | | | 0.029 | | | | | 648 | | | | | | | | 49 | | | 0.53 | | | | | | | 0.243 | | | 686 | | | 11 | | | 0.72 | | | | 0.005 | | | | | | |  |  |  |  |  |  |  |
| 27 | | | | | | 546 | | | | | | | | 159 | | | | | | | 0.56 | | | | | 0.017 | | | | | 654 | | | | | | | | 51 | | | 0.54 | | | | | | | 0.153 | | | 693 | | | 12 | | | 0.74 | | | | 0.002 | | | | | | |  |  |  |  |  |  |  |
| 28 | | | | | | 549 | | | | | | | | 159 | | | | | | | 0.56 | | | | | 0.01 | | | | | 657 | | | | | | | | 51 | | | 0.55 | | | | | | | 0.101 | | | 696 | | | 12 | | | 0.74 | | | | 0.002 | | | | | | |  |  |  |  |  |  |  |
| 29 | | | | | | 555 | | | | | | | | 159 | | | | | | | 0.56 | | | | | 0.009 | | | | | 663 | | | | | | | | 51 | | | 0.55 | | | | | | | 0.111 | | | 702 | | | 12 | | | 0.73 | | | | 0.003 | | | | | | |  |  |  |  |  |  |  |
| 30 | | | | | | 558 | | | | | | | | 161 | | | | | | | 0.56 | | | | | 0.01 | | | | | 668 | | | | | | | | 51 | | | 0.54 | | | | | | | 0.16 | | | 707 | | | 12 | | | 0.73 | | | | 0.003 | | | | | | |  |  |  |  |  |  |  |
| 31 | | | | | | 558 | | | | | | | | 162 | | | | | | | 0.56 | | | | | 0.009 | | | | | 669 | | | | | | | | 51 | | | 0.54 | | | | | | | 0.191 | | | 708 | | | 12 | | | 0.73 | | | | 0.002 | | | | | | |  |  |  |  |  |  |  |
| 32 | | | | | | 558 | | | | | | | | 162 | | | | | | | 0.56 | | | | | 0.008 | | | | | 669 | | | | | | | | 51 | | | 0.54 | | | | | | | 0.2 | | | 708 | | | 12 | | | 0.73 | | | | 0.002 | | | | | | |  |  |  |  |  |  |  |
| 33 | | | | | | 560 | | | | | | | | 163 | | | | | | | 0.56 | | | | | 0.008 | | | | | 672 | | | | | | | | 51 | | | 0.54 | | | | | | | 0.188 | | | 711 | | | 12 | | | 0.73 | | | | 0.004 | | | | | | |  |  |  |  |  |  |  |
| 34 | | | | | | 562 | | | | | | | | 165 | | | | | | | 0.56 | | | | | 0.009 | | | | | 675 | | | | | | | | 52 | | | 0.53 | | | | | | | 0.215 | | | 715 | | | 12 | | | 0.73 | | | | 0.003 | | | | | | |  |  |  |  |  |  |  |
| 35 | | | | | | 565 | | | | | | | | 165 | | | | | | | 0.56 | | | | | 0.004 | | | | | 678 | | | | | | | | 52 | | | 0.54 | | | | | | | 0.15 | | | 718 | | | 12 | | | 0.74 | | | | 0.001 | | | | | | |  |  |  |  |  |  |  |
| 36 | | | | | | 569 | | | | | | | | 165 | | | | | | | 0.57 | | | | | 0.004 | | | | | 682 | | | | | | | | 52 | | | 0.55 | | | | | | | 0.132 | | | 722 | | | 12 | | | 0.74 | | | | 0.001 | | | | | | |  |  |  |  |  |  |  |
| 37 | | | | | | 571 | | | | | | | | 165 | | | | | | | 0.57 | | | | | 0.003 | | | | | 684 | | | | | | | | 52 | | | 0.55 | | | | | | | 0.133 | | | 724 | | | 12 | | | 0.73 | | | | 0.003 | | | | | | |  |  |  |  |  |  |  |
| 38 | | | | | | 575 | | | | | | | | 165 | | | | | | | 0.57 | | | | | 0.001 | | | | | 688 | | | | | | | | 52 | | | 0.55 | | | | | | | 0.123 | | | 728 | | | 12 | | | 0.73 | | | | 0.003 | | | | | | |  |  |  |  |  |  |  |
| 39 | | | | | | 575 | | | | | | | | 165 | | | | | | | 0.58 | | | | | 7E-04 | | | | | 688 | | | | | | | | 52 | | | 0.56 | | | | | | | 0.081 | | | 728 | | | 12 | | | 0.72 | | | | 0.004 | | | | | | |  |  |  |  |  |  |  |
| 40 | | | | | | 580 | | | | | | | | 165 | | | | | | | 0.58 | | | | | 0.002 | | | | | 693 | | | | | | | | 52 | | | 0.56 | | | | | | | 0.085 | | | 733 | | | 12 | | | 0.72 | | | | 0.003 | | | | | | |  |  |  |  |  |  |  |
| 41 | | | | | | 583 | | | | | | | | 166 | | | | | | | 0.57 | | | | | 0.002 | | | | | 696 | | | | | | | | 53 | | | 0.55 | | | | | | | 0.1 | | | 737 | | | 12 | | | 0.73 | | | | 0.002 | | | | | | |  |  |  |  |  |  |  |
| 42 | | | | | | 588 | | | | | | | | 167 | | | | | | | 0.57 | | | | | 0.002 | | | | | 701 | | | | | | | | 54 | | | 0.55 | | | | | | | 0.134 | | | 743 | | | 12 | | | 0.73 | | | | 0.003 | | | | | | |  |  |  |  |  |  |  |
| 43 | | | | | | 593 | | | | | | | | 167 | | | | | | | 0.58 | | | | | 7E-04 | | | | | 706 | | | | | | | | 54 | | | 0.55 | | | | | | | 0.13 | | | 748 | | | 12 | | | 0.72 | | | | 0.005 | | | | | | |  |  |  |  |  |  |  |
| 44 | | | | | | 600 | | | | | | | | 168 | | | | | | | 0.59 | | | | | 5E-04 | | | | | 714 | | | | | | | | 54 | | | 0.55 | | | | | | | 0.115 | | | 756 | | | 12 | | | 0.71 | | | | 0.006 | | | | | | |  |  |  |  |  |  |  |
| 45 | | | | | | 603 | | | | | | | | 168 | | | | | | | 0.59 | | | | | 3E-04 | | | | | 717 | | | | | | | | 54 | | | 0.55 | | | | | | | 0.131 | | | 759 | | | 12 | | | 0.71 | | | | 0.005 | | | | | | |  |  |  |  |  |  |  |
| 46 | | | | | | 605 | | | | | | | | 168 | | | | | | | 0.59 | | | | | 5E-04 | | | | | 719 | | | | | | | | 54 | | | 0.54 | | | | | | | 0.134 | | | 761 | | | 12 | | | 0.7 | | | | 0.006 | | | | | | |  |  |  |  |  |  |  |
| 47 | | | | | | 610 | | | | | | | | 168 | | | | | | | 0.58 | | | | | 6E-04 | | | | | 724 | | | | | | | | 54 | | | 0.54 | | | | | | | 0.15 | | | 766 | | | 12 | | | 0.7 | | | | 0.008 | | | | | | |  |  |  |  |  |  |  |
| 48 | | | | | | 613 | | | | | | | | 169 | | | | | | | 0.58 | | | | | 3E-04 | | | | | 728 | | | | | | | | 54 | | | 0.54 | | | | | | | 0.137 | | | 770 | | | 12 | | | 0.7 | | | | 0.008 | | | | | | |  |  |  |  |  |  |  |
| 49 | | | | | | 616 | | | | | | | | 169 | | | | | | | 0.58 | | | | | 8E-04 | | | | | 731 | | | | | | | | 54 | | | 0.55 | | | | | | | 0.122 | | | 773 | | | 12 | | | 0.71 | | | | 0.006 | | | | | | |  |  |  |  |  |  |  |
| 50 | | | | | | 625 | | | | | | | | 170 | | | | | | | 0.58 | | | | | 8E-04 | | | | | 741 | | | | | | | | 54 | | | 0.54 | | | | | | | 0.168 | | | 783 | | | 12 | | | 0.69 | | | | 0.011 | | | | | | |  |  |  |  |  |  |  |
| **Table S3.** Predictive performance of future imputed data as a function of days from survey. | | | | | | | | | | | | | | | | | | | | | | | | | | | | | | | | | | | | | | | | | | | | | | | | | | | | | | | | | | | |  | | |  | | | | |  | | | |  |  |  |  |
| **Student Survey Cohort** | | | | | | | | | | | | | | | | | | | | | | | | | | | | | | | | | | | | | | | | | | | | | | | | | | | | | | | | | | | | | | | | | | | | | | | |  |  |  |  |
| **Days Before Survey** | | | | **N SI < 1** | | | | **N SI > 1** | | | | | | | **AUC** | | | | | | | **P value** | | | | | | | **N SI < 2** | | | **N SI > 2** | | | | | | | | | **AUC** | | | | | | **P value** | | | | | | | **N SI < 3** | | | | | | **N SI > 3** | | | **AUC** | | | | | **P value** | | | |  |  |  |  |
| 1 | | | | 60 | | | | 35 | | | | | | | 0.47 | | | | | | | 0.9631 | | | | | | | 85 | | | 10 | | | | | | | | | 0.66 | | | | | | 0.097 | | | | | | | 89 | | | | | | 6 | | | 0.69 | | | | | 0.125 | | | |  |  |  |  |
| 2 | | | | 60 | | | | 35 | | | | | | | 0.58 | | | | | | | 0.2164 | | | | | | | 85 | | | 10 | | | | | | | | | 0.72 | | | | | | 0.0218 | | | | | | | 89 | | | | | | 6 | | | 0.72 | | | | | 0.0657 | | | |  |  |  |  |
| 3 | | | | 60 | | | | 35 | | | | | | | 0.58 | | | | | | | 0.2045 | | | | | | | 85 | | | 10 | | | | | | | | | 0.71 | | | | | | 0.0316 | | | | | | | 89 | | | | | | 6 | | | 0.76 | | | | | 0.0341 | | | |  |  |  |  |
| 4 | | | | 60 | | | | 35 | | | | | | | 0.57 | | | | | | | 0.2277 | | | | | | | 85 | | | 10 | | | | | | | | | 0.7 | | | | | | 0.0436 | | | | | | | 89 | | | | | | 6 | | | 0.76 | | | | | 0.0301 | | | |  |  |  |  |
| 5 | | | | 60 | | | | 35 | | | | | | | 0.59 | | | | | | | 0.1272 | | | | | | | 85 | | | 10 | | | | | | | | | 0.72 | | | | | | 0.0245 | | | | | | | 89 | | | | | | 6 | | | 0.77 | | | | | 0.0236 | | | |  |  |  |  |
| 6 | | | | 60 | | | | 35 | | | | | | | 0.59 | | | | | | | 0.128 | | | | | | | 85 | | | 10 | | | | | | | | | 0.7 | | | | | | 0.038 | | | | | | | 89 | | | | | | 6 | | | 0.79 | | | | | 0.0146 | | | |  |  |  |  |
| 7 | | | | 60 | | | | 35 | | | | | | | 0.61 | | | | | | | 0.0856 | | | | | | | 85 | | | 10 | | | | | | | | | 0.71 | | | | | | 0.0267 | | | | | | | 89 | | | | | | 6 | | | 0.79 | | | | | 0.0166 | | | |  |  |  |  |
| 8 | | | | 60 | | | | 35 | | | | | | | 0.6 | | | | | | | 0.0993 | | | | | | | 85 | | | 10 | | | | | | | | | 0.7 | | | | | | 0.0407 | | | | | | | 89 | | | | | | 6 | | | 0.79 | | | | | 0.0161 | | | |  |  |  |  |
| 9 | | | | 60 | | | | 35 | | | | | | | 0.6 | | | | | | | 0.0998 | | | | | | | 85 | | | 10 | | | | | | | | | 0.72 | | | | | | 0.0243 | | | | | | | 89 | | | | | | 6 | | | 0.79 | | | | | 0.0139 | | | |  |  |  |  |
| 10 | | | | 60 | | | | 35 | | | | | | | 0.58 | | | | | | | 0.1732 | | | | | | | 85 | | | 10 | | | | | | | | | 0.7 | | | | | | 0.0387 | | | | | | | 89 | | | | | | 6 | | | 0.78 | | | | | 0.0169 | | | |  |  |  |  |
| 11 | | | | 60 | | | | 35 | | | | | | | 0.58 | | | | | | | 0.2048 | | | | | | | 85 | | | 10 | | | | | | | | | 0.69 | | | | | | 0.0478 | | | | | | | 89 | | | | | | 6 | | | 0.79 | | | | | 0.0137 | | | |  |  |  |  |
| 12 | | | | 60 | | | | 35 | | | | | | | 0.59 | | | | | | | 0.1419 | | | | | | | 85 | | | 10 | | | | | | | | | 0.7 | | | | | | 0.034 | | | | | | | 89 | | | | | | 6 | | | 0.8 | | | | | 0.0119 | | | |  |  |  |  |
| 13 | | | | 60 | | | | 35 | | | | | | | 0.58 | | | | | | | 0.1818 | | | | | | | 85 | | | 10 | | | | | | | | | 0.68 | | | | | | 0.061 | | | | | | | 89 | | | | | | 6 | | | 0.8 | | | | | 0.0126 | | | |  |  |  |  |
| 14 | | | | 60 | | | | 35 | | | | | | | 0.58 | | | | | | | 0.1794 | | | | | | | 85 | | | 10 | | | | | | | | | 0.67 | | | | | | 0.0907 | | | | | | | 89 | | | | | | 6 | | | 0.78 | | | | | 0.02 | | | |  |  |  |  |
| **COVID-19 Survey Cohort** | | | | | | | | | | | | | | | | | | | | | | | | | | | | | | | | | | | | | | | | | | | | | | | | | | | | | | | | | | | | | | | | | | | | | | | |  |  |  |  |
| **Days Before Survey** | | | | **N SI < 1** | | | | | | | **N SI > 1** | | | | | | | **AUC** | | | | | | **P value** | | | | | **N SI < 2** | | | | | | | | | | | **N SI > 2** | | | | | | **AUC** | | | | **P value** | | | | **N SI < 3** | | | | | | **N SI > 3** | | | **AUC** | | | | | **P value** | | | |  |  |  |  |
| 1 | | | | 383 | | | | | | | 97 | | | | | | | 0.57 | | | | | | 0.0291 | | | | | 448 | | | | | | | | | | | 32 | | | | | | 0.54 | | | | 0.4261 | | | | 476 | | | | | | 4 | | | 0.75 | | | | | 0.0815 | | | |  |  |  |  |
| 2 | | | | 383 | | | | | | | 97 | | | | | | | 0.59 | | | | | | 0.0062 | | | | | 448 | | | | | | | | | | | 32 | | | | | | 0.55 | | | | 0.3302 | | | | 476 | | | | | | 4 | | | 0.77 | | | | | 0.0596 | | | |  |  |  |  |
| 3 | | | | 383 | | | | | | | 97 | | | | | | | 0.6 | | | | | | 0.0014 | | | | | 448 | | | | | | | | | | | 32 | | | | | | 0.45 | | | | 0.9972 | | | | 476 | | | | | | 4 | | | 0.74 | | | | | 0.0991 | | | |  |  |  |  |
| 4 | | | | 383 | | | | | | | 97 | | | | | | | 0.61 | | | | | | 9.00E-04 | | | | | 448 | | | | | | | | | | | 32 | | | | | | 0.43 | | | | 0.9996 | | | | 476 | | | | | | 4 | | | 0.7 | | | | | 0.1809 | | | |  |  |  |  |
| 5 | | | | 383 | | | | | | | 97 | | | | | | | 0.61 | | | | | | 7.00E-04 | | | | | 448 | | | | | | | | | | | 32 | | | | | | 0.58 | | | | 0.1156 | | | | 476 | | | | | | 4 | | | 0.67 | | | | | 0.249 | | | |  |  |  |  |
| 6 | | | | 383 | | | | | | | 97 | | | | | | | 0.61 | | | | | | 0.001 | | | | | 448 | | | | | | | | | | | 32 | | | | | | 0.59 | | | | 0.0978 | | | | 476 | | | | | | 4 | | | 0.7 | | | | | 0.1788 | | | |  |  |  |  |
| 7 | | | | 383 | | | | | | | 97 | | | | | | | 0.61 | | | | | | 7.00E-04 | | | | | 448 | | | | | | | | | | | 32 | | | | | | 0.59 | | | | 0.0893 | | | | 476 | | | | | | 4 | | | 0.72 | | | | | 0.1307 | | | |  |  |  |  |
| 8 | | | | 383 | | | | | | | 97 | | | | | | | 0.61 | | | | | | 0.0022 | | | | | 448 | | | | | | | | | | | 32 | | | | | | 0.58 | | | | 0.1617 | | | | 476 | | | | | | 4 | | | 0.72 | | | | | 0.1314 | | | |  |  |  |  |
| 9 | | | | 383 | | | | | | | 97 | | | | | | | 0.61 | | | | | | 1.00E-04 | | | | | 448 | | | | | | | | | | | 32 | | | | | | 0.57 | | | | 0.1615 | | | | 476 | | | | | | 4 | | | 0.71 | | | | | 0.1563 | | | |  |  |  |  |
| 10 | | | | 383 | | | | | | | 97 | | | | | | | 0.62 | | | | | | 2.00E-04 | | | | | 448 | | | | | | | | | | | 32 | | | | | | 0.59 | | | | 0.0997 | | | | 476 | | | | | | 4 | | | 0.72 | | | | | 0.1251 | | | |  |  |  |  |
| 11 | | | | 383 | | | | | | | 97 | | | | | | | 0.63 | | | | | | 1.00E-04 | | | | | 448 | | | | | | | | | | | 32 | | | | | | 0.59 | | | | 0.0825 | | | | 476 | | | | | | 4 | | | 0.74 | | | | | 0.0991 | | | |  |  |  |  |
| 12 | | | | 383 | | | | | | | 97 | | | | | | | 0.63 | | | | | | 0 | | | | | 448 | | | | | | | | | | | 32 | | | | | | 0.59 | | | | 0.0939 | | | | 476 | | | | | | 4 | | | 0.77 | | | | | 0.0614 | | | |  |  |  |  |
| 13 | | | | 383 | | | | | | | 97 | | | | | | | 0.62 | | | | | | 2.00E-04 | | | | | 448 | | | | | | | | | | | 32 | | | | | | 0.59 | | | | 0.1046 | | | | 476 | | | | | | 4 | | | 0.79 | | | | | 0.0427 | | | |  |  |  |  |
| 14 | | | | 383 | | | | | | | 97 | | | | | | | 0.62 | | | | | | 3.00E-04 | | | | | 448 | | | | | | | | | | | 32 | | | | | | 0.59 | | | | 0.1054 | | | | 476 | | | | | | 4 | | | 0.78 | | | | | 0.0541 | | | |  |  |  |  |
| **Combined Cohort** | | | | | | | | | | | | | | | | | | | | | | | | | | | | | | | | | | | | | | | | | | | | | | | | | | | | | | | | | | | | | | | | | | | | | | | |  |  |  |  |
| **Days Before Survey** | | | | **N SI < 1** | | | | | | | | | **N SI > 1** | | | | | **AUC** | | | | | | **P value** | | | | | **N SI < 2** | | | | | | | | | | | **N SI > 2** | | | | | | **AUC** | | | | **P value** | | | | **N SI < 3** | | | | | | **N SI > 3** | | | **AUC** | | | | **P value** | | | | |  |  |  |  |
| 1 | | | | 443 | | | | | | | | | 132 | | | | | 0.57 | | | | | | 0.0102 | | | | | 533 | | | | | | | | | | | 42 | | | | | | 0.58 | | | | 0.0833 | | | | 565 | | | | | | 10 | | | 0.77 | | | | 0.0015 | | | | |  |  |  |  |
| 2 | | | | 443 | | | | | | | | | 132 | | | | | 0.6 | | | | | | 2.00E-04 | | | | | 533 | | | | | | | | | | | 42 | | | | | | 0.6 | | | | 0.0234 | | | | 565 | | | | | | 10 | | | 0.81 | | | | 4.00E-04 | | | | |  |  |  |  |
| 3 | | | | 443 | | | | | | | | | 132 | | | | | 0.61 | | | | | | 2.00E-04 | | | | | 533 | | | | | | | | | | | 42 | | | | | | 0.6 | | | | 0.0254 | | | | 565 | | | | | | 10 | | | 0.82 | | | | 2.00E-04 | | | | |  |  |  |  |
| 4 | | | | 443 | | | | | | | | | 132 | | | | | 0.62 | | | | | | 0 | | | | | 533 | | | | | | | | | | | 42 | | | | | | 0.62 | | | | 0.0112 | | | | 565 | | | | | | 10 | | | 0.81 | | | | 5.00E-04 | | | | |  |  |  |  |
| 5 | | | | 443 | | | | | | | | | 132 | | | | | 0.63 | | | | | | 0 | | | | | 533 | | | | | | | | | | | 42 | | | | | | 0.63 | | | | 0.0041 | | | | 565 | | | | | | 10 | | | 0.8 | | | | 4.00E-04 | | | | |  |  |  |  |
| 6 | | | | 443 | | | | | | | | | 132 | | | | | 0.62 | | | | | | 0 | | | | | 533 | | | | | | | | | | | 42 | | | | | | 0.63 | | | | 0.006 | | | | 565 | | | | | | 10 | | | 0.81 | | | | 4.00E-04 | | | | |  |  |  |  |
| 7 | | | | 443 | | | | | | | | | 132 | | | | | 0.63 | | | | | | 0 | | | | | 533 | | | | | | | | | | | 42 | | | | | | 0.63 | | | | 0.0047 | | | | 565 | | | | | | 10 | | | 0.82 | | | | 5.00E-04 | | | | |  |  |  |  |
| 8 | | | | 443 | | | | | | | | | 132 | | | | | 0.63 | | | | | | 0 | | | | | 533 | | | | | | | | | | | 42 | | | | | | 0.62 | | | | 0.0126 | | | | 565 | | | | | | 10 | | | 0.82 | | | | 7.00E-04 | | | | |  |  |  |  |
| 9 | | | | 443 | | | | | | | | | 132 | | | | | 0.63 | | | | | | 0 | | | | | 533 | | | | | | | | | | | 42 | | | | | | 0.62 | | | | 0.0119 | | | | 565 | | | | | | 10 | | | 0.81 | | | | 9.00E-04 | | | | |  |  |  |  |
| 10 | | | | 443 | | | | | | | | | 132 | | | | | 0.64 | | | | | | 0 | | | | | 533 | | | | | | | | | | | 42 | | | | | | 0.63 | | | | 0.0073 | | | | 565 | | | | | | 10 | | | 0.82 | | | | 2.00E-04 | | | | |  |  |  |  |
| 11 | | | | 443 | | | | | | | | | 132 | | | | | 0.64 | | | | | | 0 | | | | | 533 | | | | | | | | | | | 42 | | | | | | 0.63 | | | | 0.0057 | | | | 565 | | | | | | 10 | | | 0.83 | | | | 1.00E-04 | | | | |  |  |  |  |
| 12 | | | | 443 | | | | | | | | | 132 | | | | | 0.64 | | | | | | 0 | | | | | 533 | | | | | | | | | | | 42 | | | | | | 0.63 | | | | 0.0058 | | | | 565 | | | | | | 10 | | | 0.84 | | | | 0 | | | | |  |  |  |  |
| 13 | | | | 443 | | | | | | | | | 132 | | | | | 0.64 | | | | | | 0 | | | | | 533 | | | | | | | | | | | 42 | | | | | | 0.62 | | | | 0.0065 | | | | 565 | | | | | | 10 | | | 0.85 | | | | 0 | | | | |  |  |  |  |
| 14 | | | | 443 | | | | | | | | | 132 | | | | | 0.64 | | | | | | 0 | | | | | 533 | | | | | | | | | | | 42 | | | | | | 0.62 | | | | 0.009 | | | | 565 | | | | | | 10 | | | 0.84 | | | | 0 | | | | |  |  |  |  |
| **Table S4.** Top 20 over-represented terms in personal model scored responses to suicidal mentions. | | | | | | | | | | | | | | | | | | | | | | | | | | | | | | | | | | | | | | | | | | | | | | | | | | | | | | | | | | | | | | | | |  | | | | | |  | | | | |
| *Personally Positive Responses (Favorable)* | | | | | | | | | | | | | | | | | | | | | | | | | | | | | | | | | | | | | | | | | | | | | | | | | | | | | | | | | | | | | | | | | | | | | | | | | | | |
| **Single Term** | | | | | | | | | **Frequency** | | | | | | | | | | **Trials** | | | | | | | | **Probability** | | | | | | | | | | | **P value** | | | | | | **Two Word Pairs** | | | | | | | | | **Frequency** | | | | | | **Trials** | | | | | | **Probability** | | | | | | **P value** | | | | |
| ME | | | | | | | | | 21 | | | | | | | | | | 3373 | | | | | | | | 0.004801 | | | | | | | | | | | 0.14 | | | | | | FROM THE | | | | | | | | | 5 | | | | | | 2761 | | | | | | 0.0012 | | | | | | 0.24 | | | | |
| :( | | | | | | | | | 13 | | | | | | | | | | 3373 | | | | | | | | 0.002801 | | | | | | | | | | | 0.16 | | | | | | I WAS | | | | | | | | | 5 | | | | | | 2761 | | | | | | 0.0012 | | | | | | 0.24 | | | | |
| OUT | | | | | | | | | 13 | | | | | | | | | | 3373 | | | | | | | | 0.002801 | | | | | | | | | | | 0.16 | | | | | | PLEASE PLEASE | | | | | | | | | 5 | | | | | | 2761 | | | | | | 0.0012 | | | | | | 0.24 | | | | |
| ABOUT | | | | | | | | | 21 | | | | | | | | | | 3373 | | | | | | | | 0.005001 | | | | | | | | | | | 0.18 | | | | | | ABOUT IT | | | | | | | | | 4 | | | | | | 2761 | | | | | | 0.00096 | | | | | | 0.28 | | | | |
| FROM | | | | | | | | | 11 | | | | | | | | | | 3373 | | | | | | | | 0.0024 | | | | | | | | | | | 0.19 | | | | | | ARE SO | | | | | | | | | 4 | | | | | | 2761 | | | | | | 0.00096 | | | | | | 0.28 | | | | |
| MORE | | | | | | | | | 7 | | | | | | | | | | 3373 | | | | | | | | 0.0014 | | | | | | | | | | | 0.20 | | | | | | HOW YOU | | | | | | | | | 4 | | | | | | 2761 | | | | | | 0.00096 | | | | | | 0.28 | | | | |
| U | | | | | | | | | 17 | | | | | | | | | | 3373 | | | | | | | | 0.004001 | | | | | | | | | | | 0.20 | | | | | | LET US | | | | | | | | | 4 | | | | | | 2761 | | | | | | 0.00096 | | | | | | 0.28 | | | | |
| HAPPENED | | | | | | | | | 6 | | | | | | | | | | 3373 | | | | | | | | 0.0012 | | | | | | | | | | | 0.22 | | | | | | ME IF | | | | | | | | | 4 | | | | | | 2761 | | | | | | 0.00096 | | | | | | 0.28 | | | | |
| HAPPY | | | | | | | | | 6 | | | | | | | | | | 3373 | | | | | | | | 0.0012 | | | | | | | | | | | 0.22 | | | | | | TALK ABOUT | | | | | | | | | 4 | | | | | | 2761 | | | | | | 0.00096 | | | | | | 0.28 | | | | |
| I'M | | | | | | | | | 18 | | | | | | | | | | 3373 | | | | | | | | 0.004401 | | | | | | | | | | | 0.24 | | | | | | US KNOW | | | | | | | | | 4 | | | | | | 2761 | | | | | | 0.00096 | | | | | | 0.28 | | | | |
| GOING | | | | | | | | | 9 | | | | | | | | | | 3373 | | | | | | | | 0.002 | | | | | | | | | | | 0.24 | | | | | | WHAT HAPPENED | | | | | | | | | 4 | | | | | | 2761 | | | | | | 0.00096 | | | | | | 0.28 | | | | |
| MANY | | | | | | | | | 5 | | | | | | | | | | 3373 | | | | | | | | 0.001 | | | | | | | | | | | 0.25 | | | | | | #VITALSIGNS REPORT: | | | | | | | | | 3 | | | | | | 2761 | | | | | | 0.00072 | | | | | | 0.32 | | | | |
| THAN | | | | | | | | | 5 | | | | | | | | | | 3373 | | | | | | | | 0.001 | | | | | | | | | | | 0.25 | | | | | | ARE HERE | | | | | | | | | 3 | | | | | | 2761 | | | | | | 0.00072 | | | | | | 0.32 | | | | |
| PLEASE | | | | | | | | | 40 | | | | | | | | | | 3373 | | | | | | | | 0.010602 | | | | | | | | | | | 0.26 | | | | | | ARE INCREASING | | | | | | | | | 3 | | | | | | 2761 | | | | | | 0.00072 | | | | | | 0.32 | | | | |
| TO | | | | | | | | | 96 | | | | | | | | | | 3373 | | | | | | | | 0.026605 | | | | | | | | | | | 0.27 | | | | | | CAN TALK | | | | | | | | | 3 | | | | | | 2761 | | | | | | 0.00072 | | | | | | 0.32 | | | | |
| 4 | | | | | | | | | 4 | | | | | | | | | | 3373 | | | | | | | | 0.0008 | | | | | | | | | | | 0.29 | | | | | | COMMUNIT… X17J1HYEBL | | | | | | | | | 3 | | | | | | 2761 | | | | | | 0.00072 | | | | | | 0.32 | | | | |
| BILLION | | | | | | | | | 4 | | | | | | | | | | 3373 | | | | | | | | 0.0008 | | | | | | | | | | | 0.29 | | | | | | DEPARTMENT VISITS | | | | | | | | | 3 | | | | | | 2761 | | | | | | 0.00072 | | | | | | 0.32 | | | | |
| CAN'T | | | | | | | | | 4 | | | | | | | | | | 3373 | | | | | | | | 0.0008 | | | | | | | | | | | 0.29 | | | | | | DM ME | | | | | | | | | 3 | | | | | | 2761 | | | | | | 0.00072 | | | | | | 0.32 | | | | |
| DIE | | | | | | | | | 4 | | | | | | | | | | 3373 | | | | | | | | 0.0008 | | | | | | | | | | | 0.29 | | | | | | EMERGENCY DEPARTMENT | | | | | | | | | 3 | | | | | | 2761 | | | | | | 0.00072 | | | | | | 0.32 | | | | |
| *Personally Negative Responses (Unfavorable)* | | | | | | | | | | | | | | | | | | | | | | | | | | | | | | | | | | | | | | | | | | | | | | | | | | | | | | | | | | | | | | | | | | | | | | | | | | | |
| **Single Term** | | | | | | | | | **Frequency** | | | | | | | | | | **Trials** | | | | | | | | **Probability** | | | | | | **P value** | | | | | | | | | | | **Two Word Pairs** | | | | | | | | | **Frequency** | | | | | | **Trials** | | | | | | **Probability** | | | | | **P value** | | | | | |
| HOPE | | | | | | | | | 5 | | | | | | | | | | 1627 | | | | | | | | 0.001 | | | | | | 0.025 | | | | | | | | | | | EVERYTHING WILL | | | | | | | | | 3 | | | | | | 1405 | | | | | | 0.00072 | | | | | 0.082 | | | | | |
| EVERYTHING | | | | | | | | | 7 | | | | | | | | | | 1627 | | | | | | | | 0.0018 | | | | | | 0.030 | | | | | | | | | | | BE ALRIGHT | | | | | | | | | 3 | | | | | | 1405 | | | | | | 0.00072 | | | | | 0.082 | | | | | |
| GOES | | | | | | | | | 4 | | | | | | | | | | 1627 | | | | | | | | 0.0008 | | | | | | 0.043 | | | | | | | | | | | ARE NOT | | | | | | | | | 3 | | | | | | 1405 | | | | | | 0.00072 | | | | | 0.082 | | | | | |
| YOU'LL | | | | | | | | | 4 | | | | | | | | | | 1627 | | | | | | | | 0.0008 | | | | | | 0.043 | | | | | | | | | | | GET THROUGH | | | | | | | | | 3 | | | | | | 1405 | | | | | | 0.00072 | | | | | 0.082 | | | | | |
| YOU’RE | | | | | | | | | 7 | | | | | | | | | | 1627 | | | | | | | | 0.002 | | | | | | 0.048 | | | | | | | | | | | IT WAS | | | | | | | | | 3 | | | | | | 1405 | | | | | | 0.00072 | | | | | 0.082 | | | | | |
| WILL | | | | | | | | | 11 | | | | | | | | | | 1627 | | | | | | | | 0.003801 | | | | | | 0.050 | | | | | | | | | | | OF YOU | | | | | | | | | 3 | | | | | | 1405 | | | | | | 0.00072 | | | | | 0.082 | | | | | |
| AT | | | | | | | | | 9 | | | | | | | | | | 1627 | | | | | | | | 0.003001 | | | | | | 0.060 | | | | | | | | | | | WILL BE | | | | | | | | | 5 | | | | | | 1405 | | | | | | 0.00168 | | | | | 0.091 | | | | | |
| IT | | | | | | | | | 24 | | | | | | | | | | 1627 | | | | | | | | 0.010602 | | | | | | 0.071 | | | | | | | | | | | AT THE | | | | | | | | | 4 | | | | | | 1405 | | | | | | 0.0012 | | | | | 0.091 | | | | | |
| FEELING | | | | | | | | | 3 | | | | | | | | | | 1627 | | | | | | | | 0.0006 | | | | | | 0.076 | | | | | | | | | | | DON'T DO | | | | | | | | | 8 | | | | | | 1405 | | | | | | 0.003601 | | | | | 0.139 | | | | | |
| REASON | | | | | | | | | 3 | | | | | | | | | | 1627 | | | | | | | | 0.0006 | | | | | | 0.076 | | | | | | | | | | | A BETTER | | | | | | | | | 2 | | | | | | 1405 | | | | | | 0.00048 | | | | | 0.147 | | | | | |
| GONNA | | | | | | | | | 5 | | | | | | | | | | 1627 | | | | | | | | 0.0014 | | | | | | 0.081 | | | | | | | | | | | BACK TO | | | | | | | | | 2 | | | | | | 1405 | | | | | | 0.00048 | | | | | 0.147 | | | | | |
| ANY | | | | | | | | | 4 | | | | | | | | | | 1627 | | | | | | | | 0.001 | | | | | | 0.083 | | | | | | | | | | | BE FINE | | | | | | | | | 2 | | | | | | 1405 | | | | | | 0.00048 | | | | | 0.147 | | | | | |
| ME, | | | | | | | | | 4 | | | | | | | | | | 1627 | | | | | | | | 0.001 | | | | | | 0.083 | | | | | | | | | | | DON'T EVEN | | | | | | | | | 2 | | | | | | 1405 | | | | | | 0.00048 | | | | | 0.147 | | | | | |
| BUT | | | | | | | | | 17 | | | | | | | | | | 1627 | | | | | | | | 0.007201 | | | | | | 0.086 | | | | | | | | | | | EVERYTHING IS | | | | | | | | | 2 | | | | | | 1405 | | | | | | 0.00048 | | | | | 0.147 | | | | | |
| AND | | | | | | | | | 32 | | | | | | | | | | 1627 | | | | | | | | 0.015403 | | | | | | 0.101 | | | | | | | | | | | FEELING LIKE | | | | | | | | | 2 | | | | | | 1405 | | | | | | 0.00048 | | | | | 0.147 | | | | | |
| AN | | | | | | | | | 5 | | | | | | | | | | 1627 | | | | | | | | 0.0016 | | | | | | 0.123 | | | | | | | | | | | GONNA GET | | | | | | | | | 2 | | | | | | 1405 | | | | | | 0.00048 | | | | | 0.147 | | | | | |
| I’M | | | | | | | | | 10 | | | | | | | | | | 1627 | | | | | | | | 0.004001 | | | | | | 0.123 | | | | | | | | | | | HAVE OUR | | | | | | | | | 2 | | | | | | 1405 | | | | | | 0.00048 | | | | | 0.147 | | | | | |
| DON'T | | | | | | | | | 21 | | | | | | | | | | 1627 | | | | | | | | 0.009802 | | | | | | 0.128 | | | | | | | | | | | I HOPE | | | | | | | | | 2 | | | | | | 1405 | | | | | | 0.00048 | | | | | 0.147 | | | | | |
| ALRIGHT | | | | | | | | | 4 | | | | | | | | | | 1627 | | | | | | | | 0.0012 | | | | | | 0.134 | | | | | | | | | | | I REALLY | | | | | | | | | 2 | | | | | | 1405 | | | | | | 0.00048 | | | | | 0.147 | | | | | |
| **Table S5.** Top 20 over-represented terms in responses to suicidal mentions relative to SI trajectory direction. | | | | | | | | | | | | | | | | | | | | | | | | | | | | | | | | | | | | | | | | | | | | | | | | | | | | | | | | | | | | | | | | | | | | | | | | | |  | |
| *Improving SI Trajectory Over 3 Weeks* | | | | | | | | | | | | | | | | | | | | | | | | | | | | | | | | | | | | | | | | | | | | | | | | | | | | | | | | | | | | | | | | | | | | | | | | | | | |
| **Single Term** | | | | | | | | | **Frequency** | | | | | | | | | | **Trials** | | | | | | | | **Probability** | | | | | | | | **P value** | | | | | | | **Two Word Pairs** | | | | | | | | | | | | | **Frequency** | | | | | **Trials** | | | | | **Probability** | | | | | | | | | **P value** | |
| AS | | | | | | | | | 9 | | | | | | | | | | 2534 | | | | | | | | 0.002271 | | | | | | | | 0.13 | | | | | | | THANK YOU | | | | | | | | | | | | | 4 | | | | | 2096 | | | | | 0.001091 | | | | | | | | | 0.20 | |
| &AMP; | | | | | | | | | 5 | | | | | | | | | | 2534 | | | | | | | | 0.001136 | | | | | | | | 0.16 | | | | | | | US KNOW | | | | | | | | | | | | | 4 | | | | | 2096 | | | | | 0.001091 | | | | | | | | | 0.20 | |
| TIME | | | | | | | | | 5 | | | | | | | | | | 2534 | | | | | | | | 0.001136 | | | | | | | | 0.16 | | | | | | | YOU KNOW | | | | | | | | | | | | | 4 | | | | | 2096 | | | | | 0.001091 | | | | | | | | | 0.20 | |
| KNOW | | | | | | | | | 15 | | | | | | | | | | 2534 | | | | | | | | 0.004542 | | | | | | | | 0.19 | | | | | | | #VITALSIGNS REPORT: | | | | | | | | | | | | | 3 | | | | | 2096 | | | | | 0.000818 | | | | | | | | | 0.25 | |
| JUST | | | | | | | | | 13 | | | | | | | | | | 2534 | | | | | | | | 0.003861 | | | | | | | | 0.19 | | | | | | | ARE INCREASING | | | | | | | | | | | | | 3 | | | | | 2096 | | | | | 0.000818 | | | | | | | | | 0.25 | |
| BACK | | | | | | | | | 4 | | | | | | | | | | 2534 | | | | | | | | 0.000908 | | | | | | | | 0.20 | | | | | | | DEPARTMENT VISITS | | | | | | | | | | | | | 3 | | | | | 2096 | | | | | 0.000818 | | | | | | | | | 0.25 | |
| HATE | | | | | | | | | 4 | | | | | | | | | | 2534 | | | | | | | | 0.000908 | | | | | | | | 0.20 | | | | | | | EMERGENCY DEPARTMENT | | | | | | | | | | | | | 3 | | | | | 2096 | | | | | 0.000818 | | | | | | | | | 0.25 | |
| MAN | | | | | | | | | 4 | | | | | | | | | | 2534 | | | | | | | | 0.000908 | | | | | | | | 0.20 | | | | | | | FIND OUT | | | | | | | | | | | | | 3 | | | | | 2096 | | | | | 0.000818 | | | | | | | | | 0.25 | |
| ME, | | | | | | | | | 4 | | | | | | | | | | 2534 | | | | | | | | 0.000908 | | | | | | | | 0.20 | | | | | | | FOR OPIOID | | | | | | | | | | | | | 3 | | | | | 2096 | | | | | 0.000818 | | | | | | | | | 0.25 | |
| NEW | | | | | | | | | 4 | | | | | | | | | | 2534 | | | | | | | | 0.000908 | | | | | | | | 0.20 | | | | | | | HERE IF | | | | | | | | | | | | | 3 | | | | | 2096 | | | | | 0.000818 | | | | | | | | | 0.25 | |
| SAFE | | | | | | | | | 4 | | | | | | | | | | 2534 | | | | | | | | 0.000908 | | | | | | | | 0.20 | | | | | | | HOW YOUR | | | | | | | | | | | | | 3 | | | | | 2096 | | | | | 0.000818 | | | | | | | | | 0.25 | |
| THANK | | | | | | | | | 4 | | | | | | | | | | 2534 | | | | | | | | 0.000908 | | | | | | | | 0.20 | | | | | | | IN IT | | | | | | | | | | | | | 3 | | | | | 2096 | | | | | 0.000818 | | | | | | | | | 0.25 | |
| YOU, | | | | | | | | | 4 | | | | | | | | | | 2534 | | | | | | | | 0.000908 | | | | | | | | 0.20 | | | | | | | INCREASING FIND | | | | | | | | | | | | | 3 | | | | | 2096 | | | | | 0.000818 | | | | | | | | | 0.25 | |
| 😂 | | | | | | | | | 4 | | | | | | | | | | 2534 | | | | | | | | 0.000908 | | | | | | | | 0.20 | | | | | | | KNOW IF | | | | | | | | | | | | | 3 | | | | | 2096 | | | | | 0.000818 | | | | | | | | | 0.25 | |
| GLAD | | | | | | | | | 6 | | | | | | | | | | 2534 | | | | | | | | 0.00159 | | | | | | | | 0.22 | | | | | | | NEW #VITALSIGNS | | | | | | | | | | | | | 3 | | | | | 2096 | | | | | 0.000818 | | | | | | | | | 0.25 | |
| HELP | | | | | | | | | 6 | | | | | | | | | | 2534 | | | | | | | | 0.00159 | | | | | | | | 0.22 | | | | | | | OPIOID OVERDOSES | | | | | | | | | | | | | 3 | | | | | 2096 | | | | | 0.000818 | | | | | | | | | 0.25 | |
| BETTER | | | | | | | | | 8 | | | | | | | | | | 2534 | | | | | | | | 0.002271 | | | | | | | | 0.22 | | | | | | | OUT HOW | | | | | | | | | | | | | 3 | | | | | 2096 | | | | | 0.000818 | | | | | | | | | 0.25 | |
| THOSE | | | | | | | | | 8 | | | | | | | | | | 2534 | | | | | | | | 0.002271 | | | | | | | | 0.22 | | | | | | | OVERDOSES ARE | | | | | | | | | | | | | 3 | | | | | 2096 | | | | | 0.000818 | | | | | | | | | 0.25 | |
| WHEN | | | | | | | | | 8 | | | | | | | | | | 2534 | | | | | | | | 0.002271 | | | | | | | | 0.22 | | | | | | | REPORT: EMERGENCY | | | | | | | | | | | | | 3 | | | | | 2096 | | | | | 0.000818 | | | | | | | | | 0.25 | |
| *Not Improving SI Trajectory Over 3 Weeks* | | | | | | | | | | | | | | | | | | | | | | | | | | | | | | | | | | | | | | | | | | | | | | | | | | | | | | | | | | | | | | | | | | | | | | | | | | | |
| **Single Term** | | | | | | | | | **Frequency** | | | | | | | | | | **Trials** | | | | | | | | **Probability** | | | | | | **P value** | | | | | | | | | **Two Word Pairs** | | | | | | | | | | | | | **Frequency** | | | | | **Trials** | | | | | **Probability** | | | | | | | **P value** | | | |
| DON'T | | | | | | | | | 34 | | | | | | | | | | 1870 | | | | | | | | 0.011129 | | | | | | 0.005 | | | | | | | | | PLEASE DON'T | | | | | | | | | | | | | 13 | | | | | 1570 | | | | | 0.004088 | | | | | | | 0.014 | | | |
| DO | | | | | | | | | 23 | | | | | | | | | | 1870 | | | | | | | | 0.007949 | | | | | | 0.030 | | | | | | | | | DO IT | | | | | | | | | | | | | 9 | | | | | 1570 | | | | | 0.002453 | | | | | | | 0.017 | | | |
| :( | | | | | | | | | 10 | | | | | | | | | | 1870 | | | | | | | | 0.002725 | | | | | | 0.035 | | | | | | | | | PLEASE PLEASE | | | | | | | | | | | | | 5 | | | | | 1570 | | | | | 0.001363 | | | | | | | 0.066 | | | |
| EVERYTHING | | | | | | | | | 8 | | | | | | | | | | 1870 | | | | | | | | 0.002044 | | | | | | 0.041 | | | | | | | | | ARE YOU | | | | | | | | | | | | | 6 | | | | | 1570 | | | | | 0.001908 | | | | | | | 0.083 | | | |
| ALRIGHT | | | | | | | | | 6 | | | | | | | | | | 1870 | | | | | | | | 0.001363 | | | | | | 0.045 | | | | | | | | | WHAT HAPPENED | | | | | | | | | | | | | 4 | | | | | 1570 | | | | | 0.00109 | | | | | | | 0.095 | | | |
| EVER | | | | | | | | | 6 | | | | | | | | | | 1870 | | | | | | | | 0.00159 | | | | | | 0.081 | | | | | | | | | YOU PLEASE | | | | | | | | | | | | | 4 | | | | | 1570 | | | | | 0.00109 | | | | | | | 0.095 | | | |
| ALL | | | | | | | | | 13 | | | | | | | | | | 1870 | | | | | | | | 0.004542 | | | | | | 0.090 | | | | | | | | | ABOUT YOU | | | | | | | | | | | | | 5 | | | | | 1570 | | | | | 0.001635 | | | | | | | 0.118 | | | |
| ABOUT | | | | | | | | | 14 | | | | | | | | | | 1870 | | | | | | | | 0.004997 | | | | | | 0.092 | | | | | | | | | WILL BE | | | | | | | | | | | | | 5 | | | | | 1570 | | | | | 0.001635 | | | | | | | 0.118 | | | |
| NEO | | | | | | | | | 4 | | | | | | | | | | 1870 | | | | | | | | 0.000908 | | | | | | 0.093 | | | | | | | | | A LOT | | | | | | | | | | | | | 3 | | | | | 1570 | | | | | 0.000818 | | | | | | | 0.139 | | | |
| OF | | | | | | | | | 18 | | | | | | | | | | 1870 | | | | | | | | 0.006814 | | | | | | 0.095 | | | | | | | | | BE ALRIGHT | | | | | | | | | | | | | 3 | | | | | 1570 | | | | | 0.000818 | | | | | | | 0.139 | | | |
| HAPPENED | | | | | | | | | 5 | | | | | | | | | | 1870 | | | | | | | | 0.001363 | | | | | | 0.115 | | | | | | | | | EVERYTHING WILL | | | | | | | | | | | | | 3 | | | | | 1570 | | | | | 0.000818 | | | | | | | 0.139 | | | |
| OKAY | | | | | | | | | 6 | | | | | | | | | | 1870 | | | | | | | | 0.001817 | | | | | | 0.129 | | | | | | | | | I THINK | | | | | | | | | | | | | 3 | | | | | 1570 | | | | | 0.000818 | | | | | | | 0.139 | | | |
| STAY | | | | | | | | | 6 | | | | | | | | | | 1870 | | | | | | | | 0.001817 | | | | | | 0.129 | | | | | | | | | I WILL | | | | | | | | | | | | | 3 | | | | | 1570 | | | | | 0.000818 | | | | | | | 0.139 | | | |
| THINK | | | | | | | | | 6 | | | | | | | | | | 1870 | | | | | | | | 0.001817 | | | | | | 0.129 | | | | | | | | | IN THIS | | | | | | | | | | | | | 3 | | | | | 1570 | | | | | 0.000818 | | | | | | | 0.139 | | | |
| EVEN | | | | | | | | | 3 | | | | | | | | | | 1870 | | | | | | | | 0.000681 | | | | | | 0.137 | | | | | | | | | TALK ABOUT | | | | | | | | | | | | | 3 | | | | | 1570 | | | | | 0.000818 | | | | | | | 0.139 | | | |
| FRIEND | | | | | | | | | 3 | | | | | | | | | | 1870 | | | | | | | | 0.000681 | | | | | | 0.137 | | | | | | | | | TO US | | | | | | | | | | | | | 3 | | | | | 1570 | | | | | 0.000818 | | | | | | | 0.139 | | | |
| KEEP | | | | | | | | | 3 | | | | | | | | | | 1870 | | | | | | | | 0.000681 | | | | | | 0.137 | | | | | | | | | WANNA TALK | | | | | | | | | | | | | 3 | | | | | 1570 | | | | | 0.000818 | | | | | | | 0.139 | | | |
| LOT | | | | | | | | | 3 | | | | | | | | | | 1870 | | | | | | | | 0.000681 | | | | | | 0.137 | | | | | | | | | YOU DON'T | | | | | | | | | | | | | 3 | | | | | 1570 | | | | | 0.000818 | | | | | | | 0.139 | | | |
| WANNA | | | | | | | | | 3 | | | | | | | | | | 1870 | | | | | | | | 0.000681 | | | | | | 0.137 | | | | | | | | | YOU OKAY | | | | | | | | | | | | | 3 | | | | | 1570 | | | | | 0.000818 | | | | | | | 0.139 | | | |


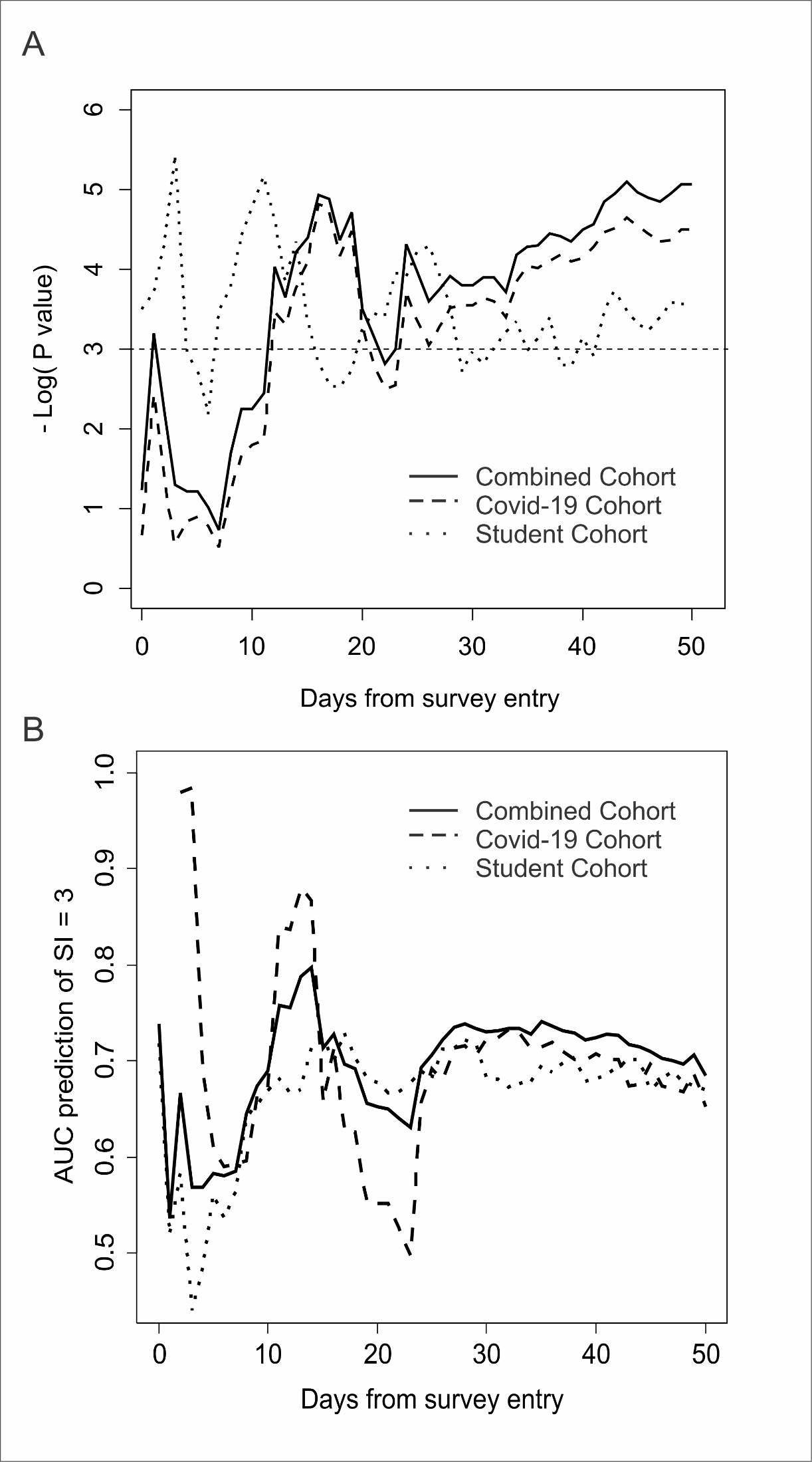


**Figure S1.** Association of SAIPH as a function of days from survey.

A.) A plot of the negative natural log of the p value (y axis) of an interaction of mean daily SAIPH score and number of posts per day on quantitative SI scores as a function of the time in days from the day of survey completion (x axis). B.)A plot of the AUC of prediction accuracy of SI scores of 3 (y axis) as a function of the number of days used to generate the average SAIPH score from the day of survey completion (x axis).


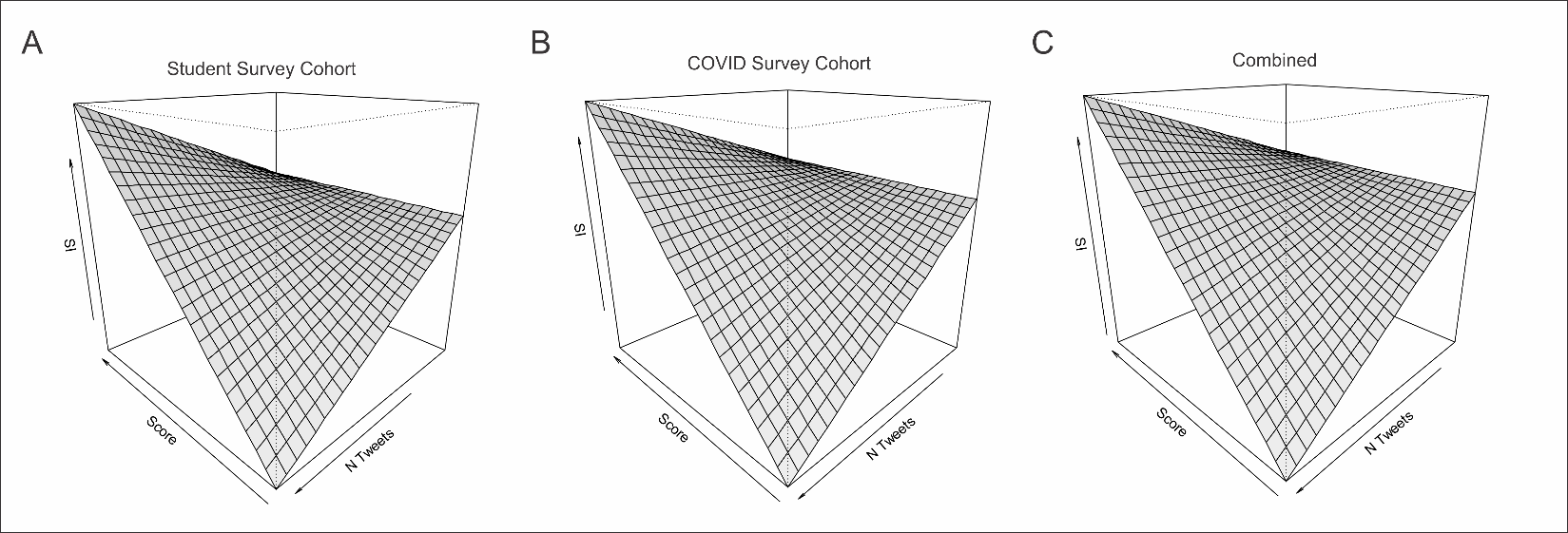


**Figure S2.** Interaction of SAIPH score and tweet number on SI.

A graphical representation of the significant interaction model observed in the Student Survey Cohort (A), the COVID Survey Cohort (B), and the combined cohort (C).


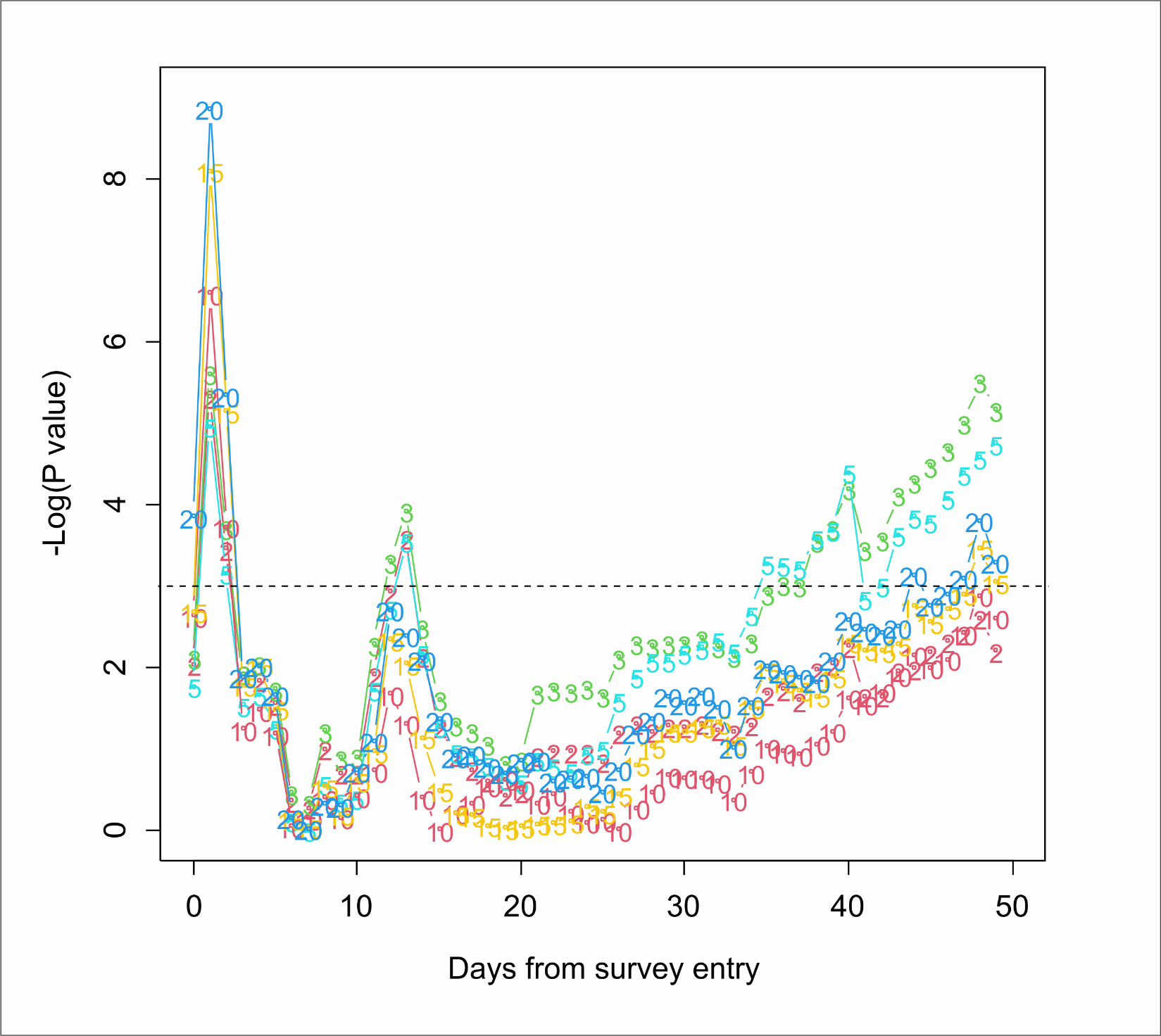


**Figure S3.** Post-hoc analysis of SAIPH score with SI as a function of minimum tweet number.

A plot depicting the negative natural log of the p value of the association of quantitative SI score with mean SAIPH score (y axis) as a function of the days from the survey entry day. The plotted numbers represent the minimum number of tweets an individual must have on that day to be included in the analysis.


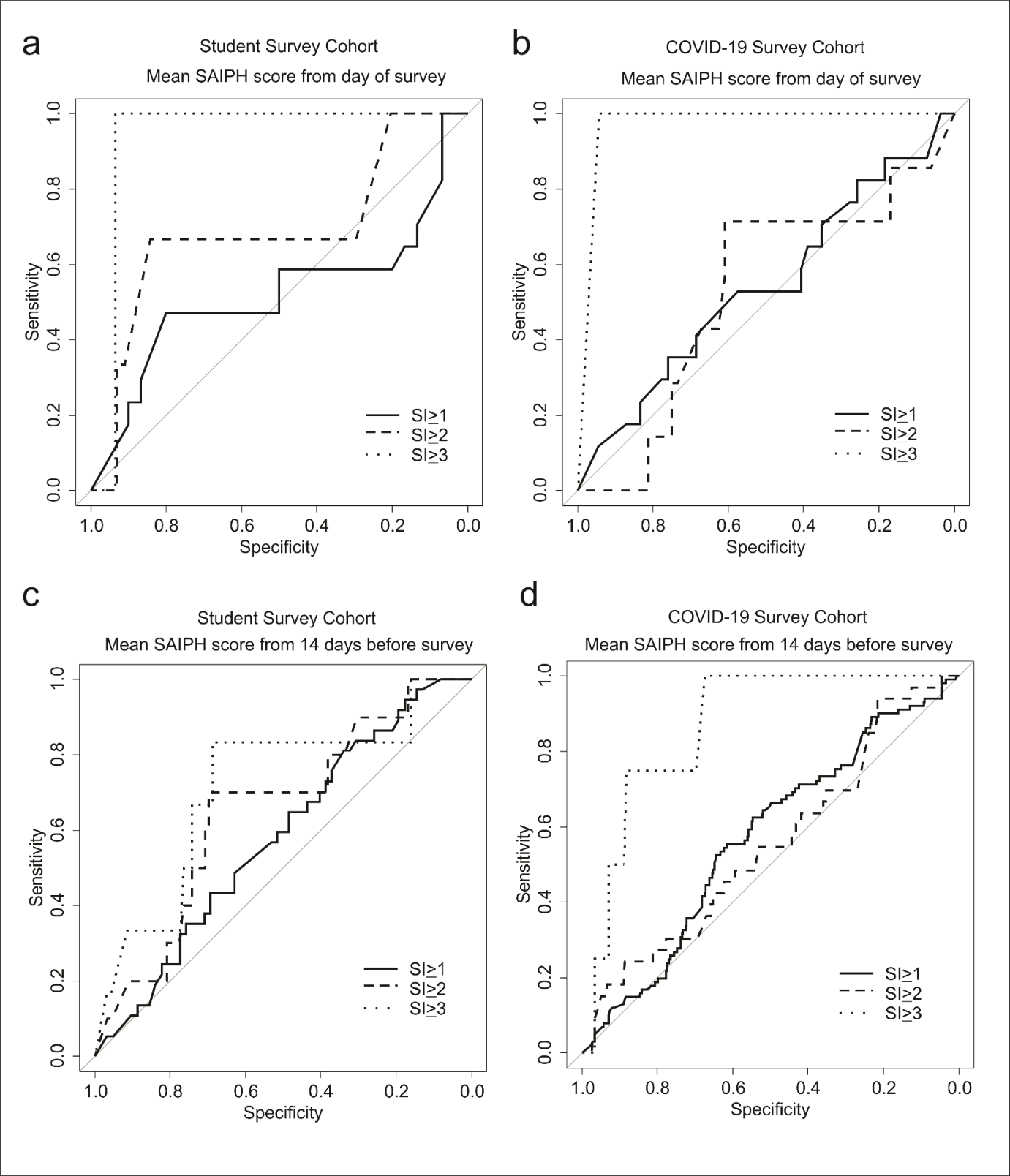


**Figure S4.** Prediction accuracy of SAIPH**.**

Receiver operator characteristic curves depicting the ability of SAIPH on the day of survey completion to predict SI scores in respondents greater than or equal to 1, 2, or 3 on the BDI and QIDS-SR16 suicide metrics in the Student Survey (a) and COVID-19 Survey (b) Cohorts and in two weeks of data preceding survey completion in the Student Survey (c) and COVID-19 Survey (d) Cohorts.

**
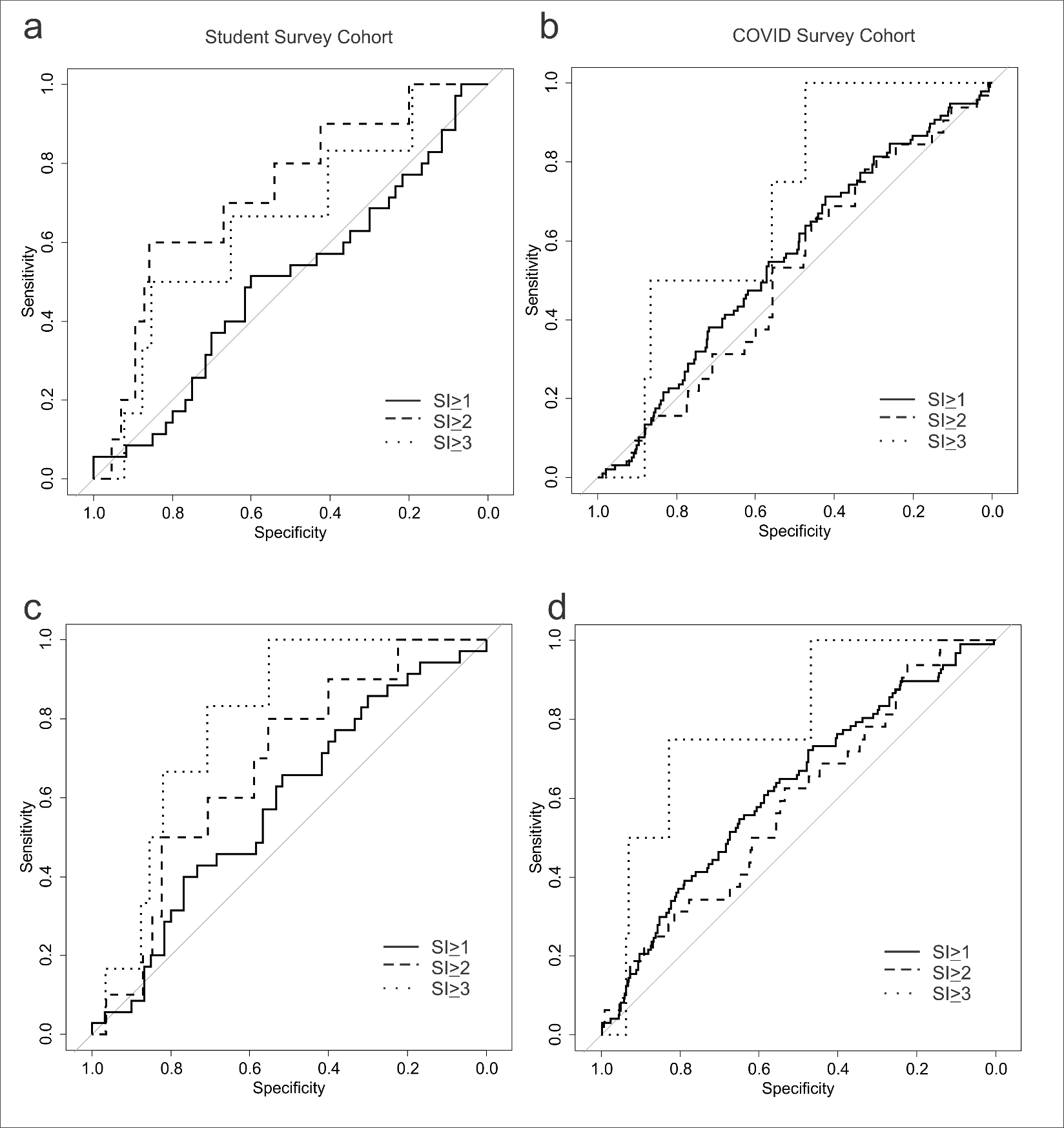
**

**Figure S5.** Prediction accuracy of future SAIPH score.

Receiver operator characteristic curves depicting the ability of future imputed SAIPH scores on the day of survey completion to predict SI scores in respondents greater than or equal to 1, 2, or 3 on the BDI and QIDS-SR16 suicide metrics in the Student Survey (a) and COVID-19 Survey (b) Cohorts and in two weeks of data preceding survey completion in the Student Survey (c) and COVID-19 Survey (d) Cohorts.


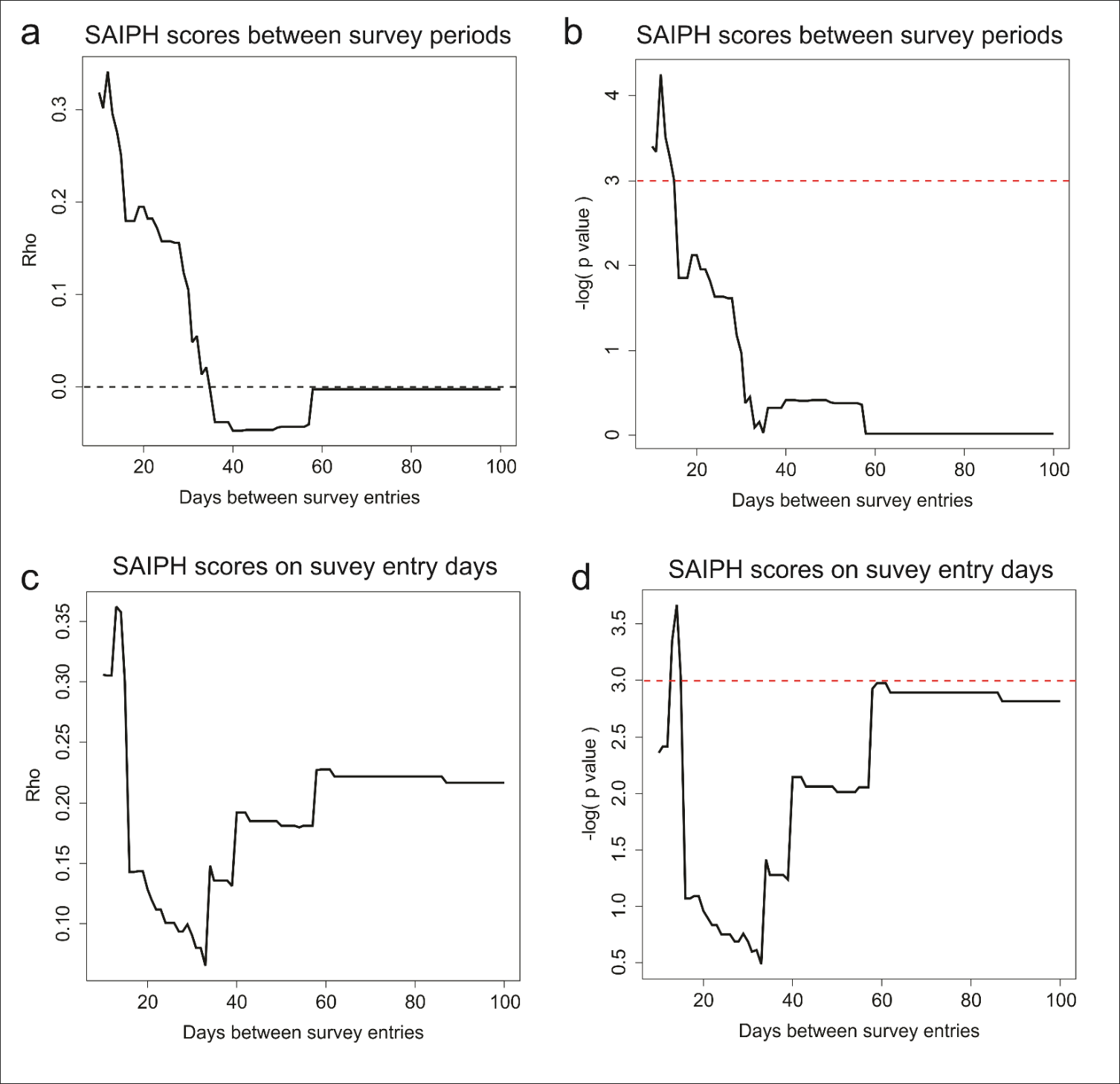


**Figure S6.** Association of slope of SAIPH with slope of changing SI over time.

A plot of the spearman’s rho (a) and negative natural log of the p value (b) derived from correlation of the slope of all SAIPH scores between survey entry dates with the slope of SI scores (y axis) as a function of the duration of time in days between survey entry points (x axis). A plot of the spearman’s rho (c) and negative natural log of the p value (d) derived from correlation of the slope of SAIPH scores on survey entry days with the slope of SI scores (y axis) as a function of the duration of time in days between survey entry points (x axis). The dashed horizontal black line (a) denotes a correlation of zero, while the dashed horizontal red lines (b, d) denotes the threshold of significance below an a of 0.05)


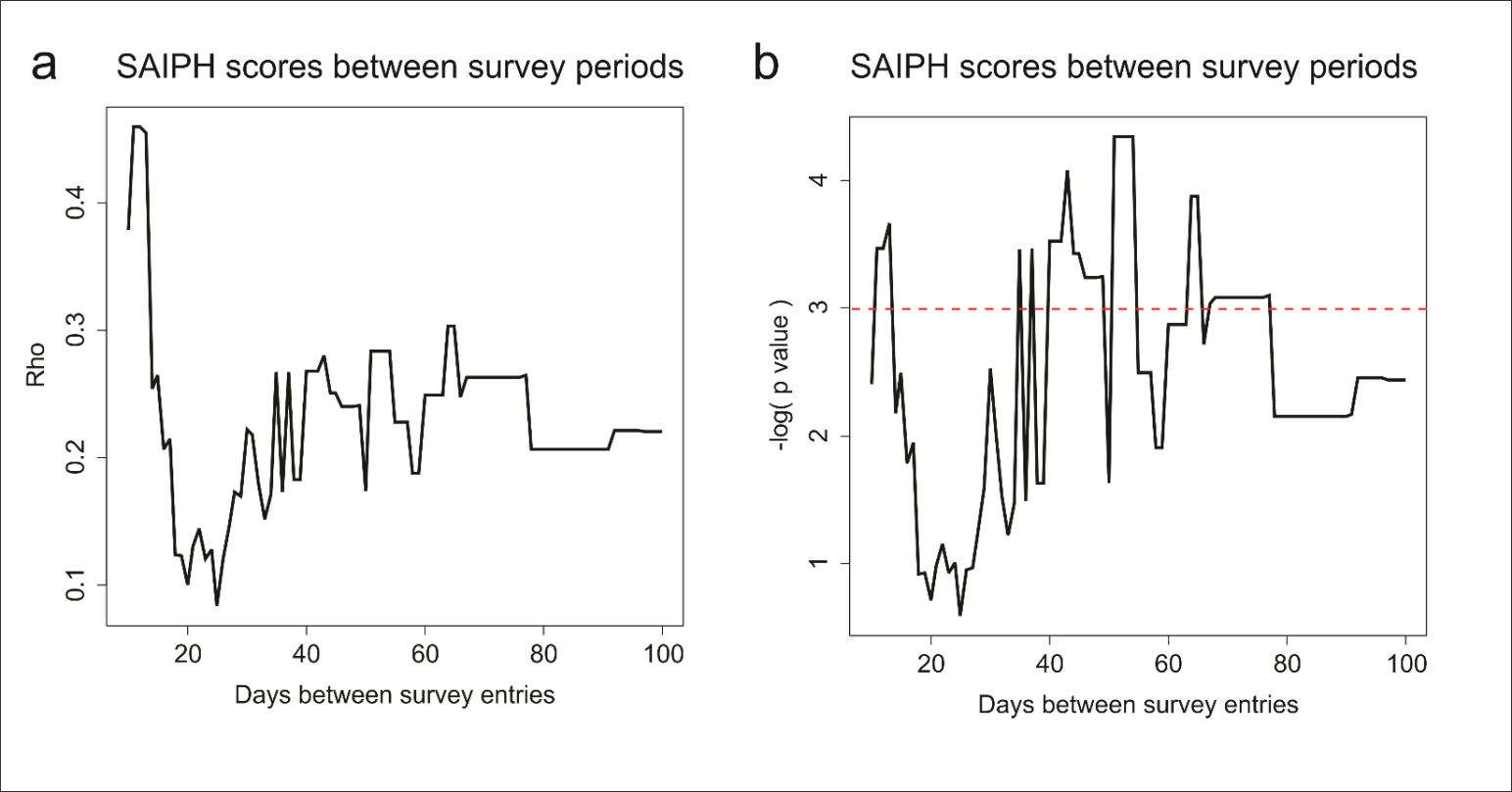


**Figure S7.** Association of slope of SAIPH with slope of changing perceived stress over time.

A plot of the spearman’s rho (a) and negative natural log of the p value (b) derived from correlation of the slope of all SAIPH scores between survey entry dates with the slope of PSS scores (y axis) as a function of the duration of time in days between survey entry points (x axis). Associations were performed on the log transformed Z score of stress slopes and score slopes to improve data normality. The dashed horizontal red line (b) denotes the threshold of significance below an a of 0.05.


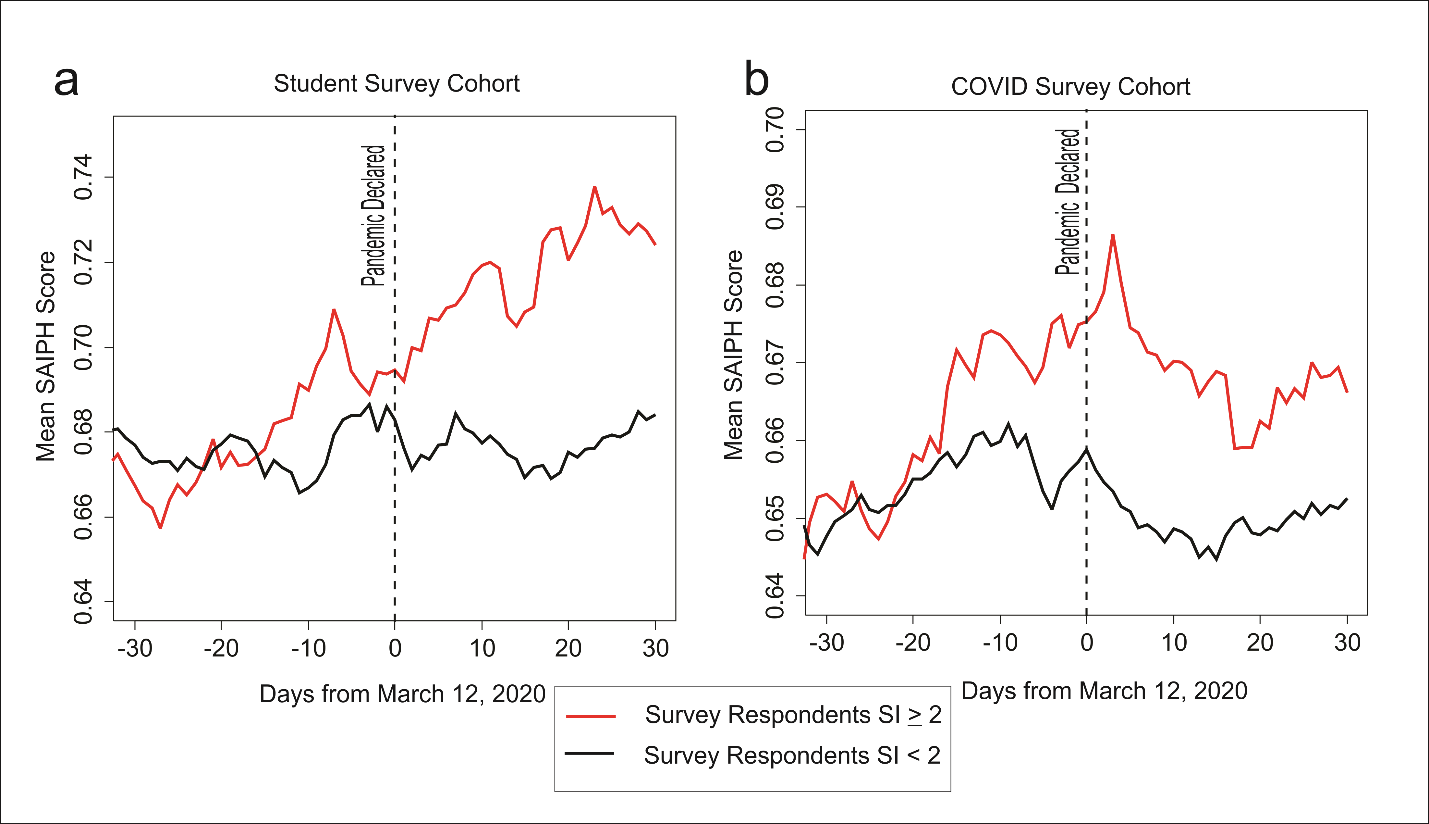


**Figure S8**. SAIPH profiles before and after COVID-19 state of emergency declaration.

Plots showing mean SAIPH score in survey respondents with SI metrics of 2 or higher (red) and below 2 (black) for the Student Survey (a) and COVID-19 Survey (b) cohorts. Vertical dashed black lines depict the date at which the state of emergency was declared, after which most social distancing and business shut downs began.


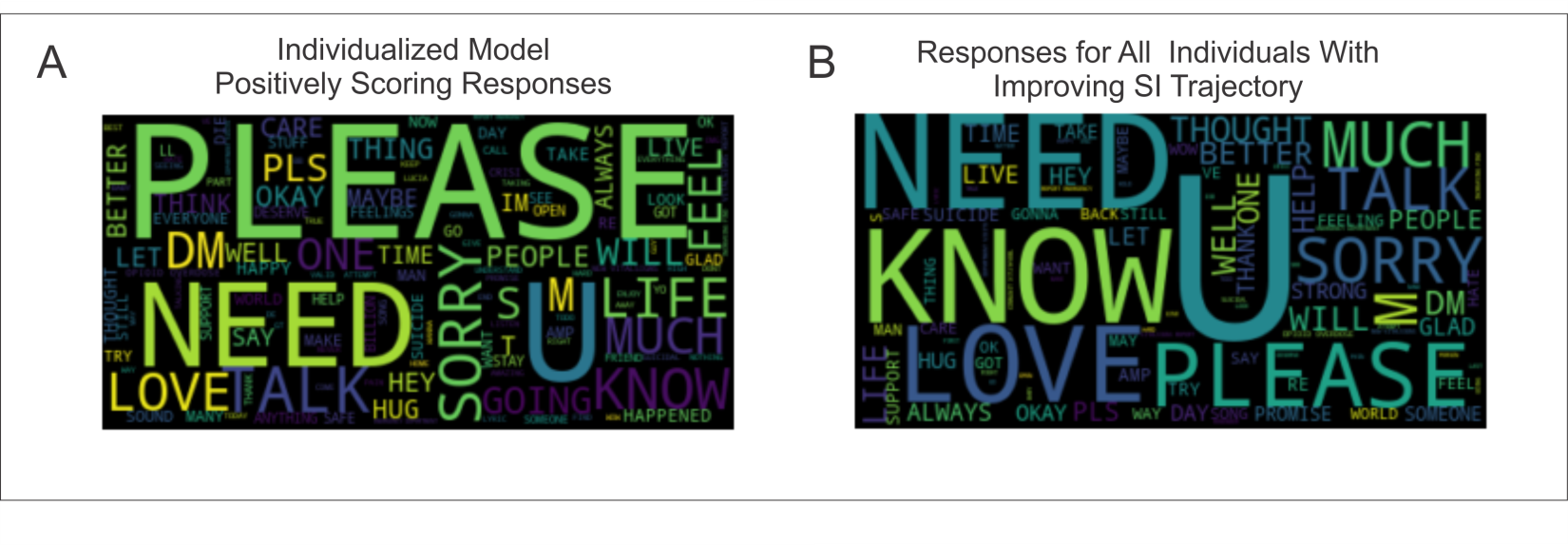


**Figure S9**. Word clouds of responses to suicidal mentions in individuals with improving SI trajectory.

A.)A word cloud generated from those responses to suicidal mentioners in the combined corpus from 2016-2022 in responses with a personalized score below a threshold of 0.24, denoting a positive response. B.)A word cloud generated from those responses to suicidal mentioners in the combined corpus from 2016-2022 in responses to people who had a negative slope of their mean daily SAIPH score as a function of time in the 21 days following suicidal mention. Word clouds were generated using the word cloud package in python.
